# Supplementary material for: Genome-wide association study identifies genetic loci for self-reported habitual sleep duration supported by accelerometer-derived estimates
Source: Nat Commun. 2019 Mar 7;10:1100. doi: 10.1038/s41467-019-08917-4 (PMC6405943; doi:10.1038/s41467-019-08917-4)
Supplement: Supplementary file 2 — Supplementary Information [file 41467_2019_8917_MOESM2_ESM.pdf]

Supplementary Information

Genome-wide association study identifies genetic loci for self-reported habitual sleep duration supported by accelerometer-derived estimates

Dashti et al.

Table of Contents

SUPPLEMENTARY METHODS.. 3

SUPPLEMENTARY FIGURES 5

SUPPLEMENTARY TABLES.. 17

Supplementary Method 1. Medication lists.

1. Sleep medications: oxazepam, meprobamate, medazepam, bromazepam, lorazepam, clobazam, chlormezanone, temazepam, nitrazepam, lormetazepam, diazepam, zopiclone, triclofos, methyprylone, prazepam, triazolam, ketazolam, dichloralphenazone, clomethiazole, zaleplon, butobarbital.

2. Antidepressants: amitriptyline, citalopram, fluoxetine, sertraline, venlafaxine, dosulepin, paroxetine, mirtazapine, escitalopram, trazodone, prozac, seroxat, cipralex, duloxetine, lofepramine, clomipramine, nortriptyline, imipramine, dothiepin, cipramil, amitriptyline, prothiaden, trimipramine, lustral, reboxetine, zispin, cymbalta, anafranil, doxepin, moclobemide, phenelzine, fluvoxamine, yentreve, triptafen, surmontil, tranylcypromine, allegron, edronax, molipaxin, mianserin, nardil, faverin, nefazodone, amitriptyline+chlordiazepoxide, isocarboxazid, manerix, maoi, sinequan, tranylcypromine+trifluoperazine, ludiomil, norval, tryptizol, and fluphenazine hydrochloride+nortriptyline.

3. Antipsychotics: prochlorperazine, olanzapine, quetiapine, risperidone, chlorpromazine, trifluoperazine, amisulpride, sulpiride, seroquel, haloperidol, aripiprazole, stelazine, depixol, flupentixol, clozapine, promazine, risperdal, modecate, fluanxol, flupenthixol, zyprexa, zuclopenthixol, clopixol, largactil, abilify, fluphenazine, haldol, serenace, clozaril, cpz, perphenazine, levomepromazine, pericyazine, dolmatil, fentazin, fluphenazine, benperidol, pimozide, zaponex, denzapine, neulactil, thioridazine, dozic, fluspirilene, panadeine, and sertindole.

4. Anxiolytics: zopiclone, diazepam, temazepam, zolpidem, nitrazepam, lorazepam, hydroxyzine, zimovane, phenergan, promethazine, buspirone, atarax, oxazepam, loprazolam, chlordiazepoxide, lormetazepam, ucerax, stilnoct, diazepam, buspar, alprazolam, librium, xanax, meprate, dalmane, clomethiazole, meprobamate, welldorm, amitriptyline+chlordiazepoxide, flurazepam, heminevrin, medazepam, neulactil, sinequan, almazine, atensine, carisoma, chloractil, chloral, dichloralphenazone, dormonoct, methyprylone, mogadon, rohypnol, tryptizol.

Supplementary Method 2. Criteria for identifying participants with psychiatric disorders (https://www.biorxiv.org/content/biorxiv/early/2017/08/01/168732.full.pdf).

Cases of psychiatric disorder was determined using any of the following definitions (derived from Howard et al.):

1) ICD-10 codes for major depressive disorder (F32, F33), bipolar disorder (F30, F31), schizophrenia (F20-F29), autism (F84.0, F84.3, F84.5), intellectual disability (F70.0, F70.1, F70.9), anxiety disorder (F40 - F43), multiple personality disorder (F44.8), or mood disorder (F30 - F39).

2) Self-reported antidepressant, antipsychotic, or anxiolytic use at nurse-led interview.

3) Self-reported depression, major depressive disorder, bipolar disorder, or schizophrenia at nurse-led interview.

4) “Broad depression”: responded yes to the question “Have you ever seen a general practitioner (GP) for nerves, anxiety, tension or depression?” and yes to either, “have you ever had a time when you were feeling depressed or down for at least a whole week,” or, “Have you ever had a time when you were uninterested in things or unable to enjoy the things you used to for at least a whole week?” lasting for more than 1 week.

5) Questionnaire-assessed bipolar disorder (Smith algorithm[1]): responded yes to the question, “Have you ever had a period of time lasting at least two days when you were feeling so good, "high", excited or "hyper" that other people thought you were not your normal self or you were so "hyper" that you got into trouble?” or “Have you ever had a period of time lasting at least two days when you were so irritable that you found yourself shouting at people or starting fights or arguments?” for a duration of at least one week and with at least 3 manic/hyper symptoms.

[1] *Smith, D.J. et al. Prevalence and characteristics of probable major depression and bipolar disorder within UK Biobank: cross-sectional study of 172,751 participants. PLoS ONE 8,e75362 (2013).*

Supplementary Figure 1. Q-Q plots for genome-wide association of sleep traits. Q-Q plots show the expected verses observed *P* values from our association analysis. Lambda inflation values (λ) were computed using GenABEL in R.

a. Sleep duration (λ = 1.432)

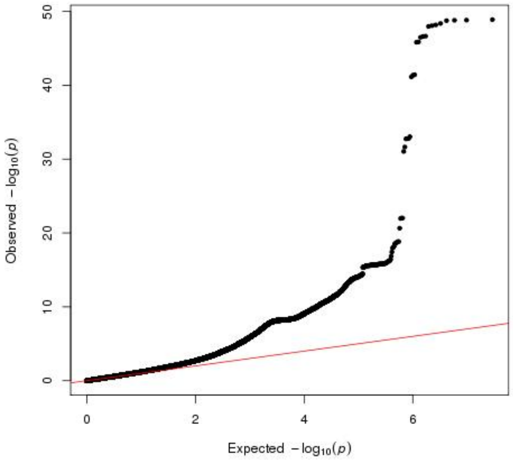

b. Sleep duration (add' exclusions) (λ = 1.344)

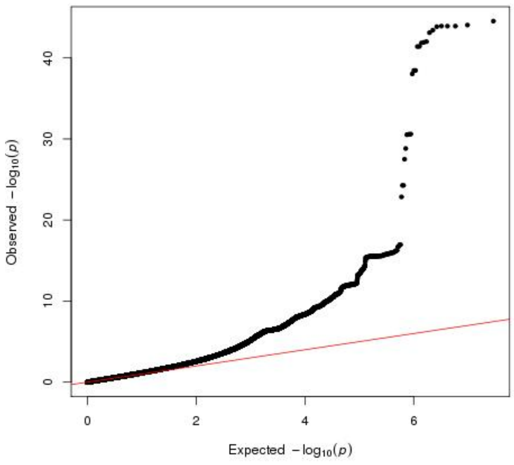

c. Sleep duration (men) (λ = 1.177)

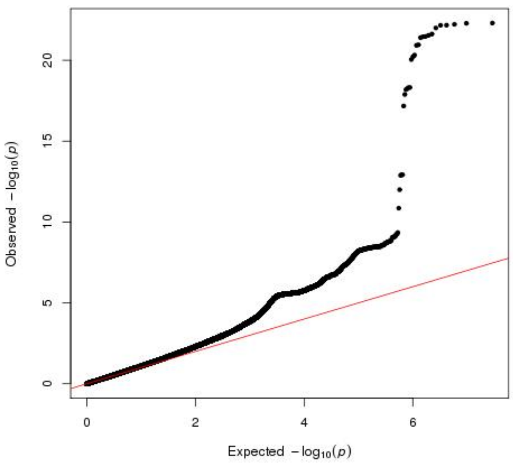

d. Sleep duration (women) (λ = 1.229)

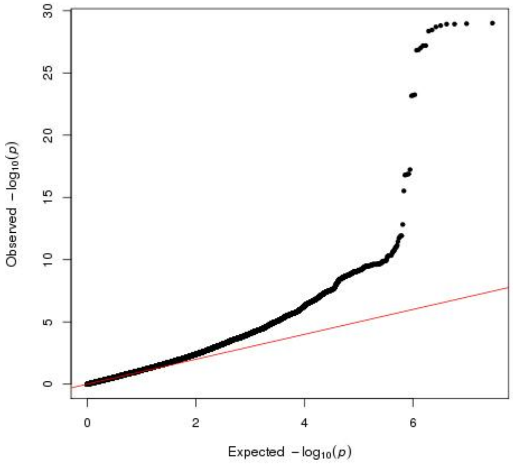

e. Short sleep (λ = 1.262)

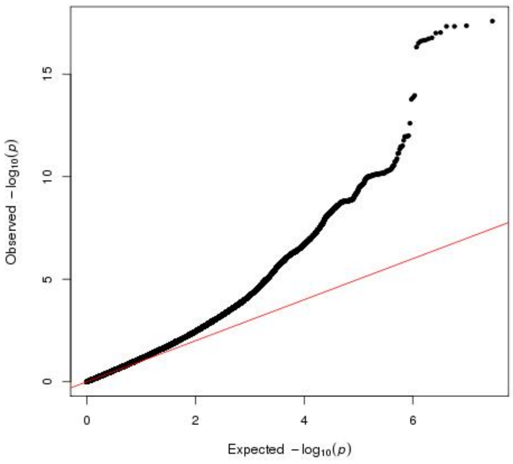

f. Long sleep (λ = 1.160)

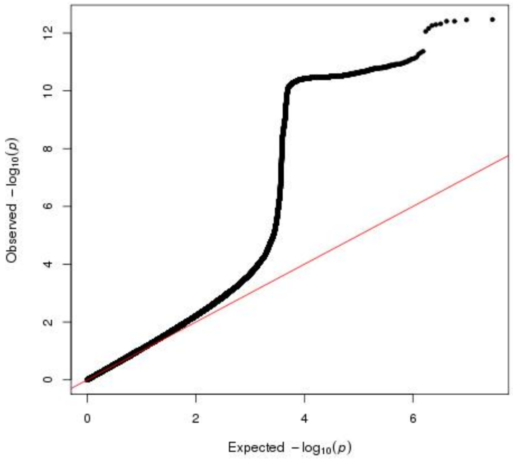

Supplementary Figure 2. Manhattan plot for genome-wide association analysis of sleep duration with additional exclusions (*n* =326,224). Manhattan plot shows the -log$_{10}$ *P* values (y-axis) for all genotyped and imputed SNPs passing quality control, plotted by chromosome (x-axis). Blue peaks represent genome-wide significant loci. Novel loci are annotated. Horizontal red line denotes genome-wide significance (5 x 10^{-8}).

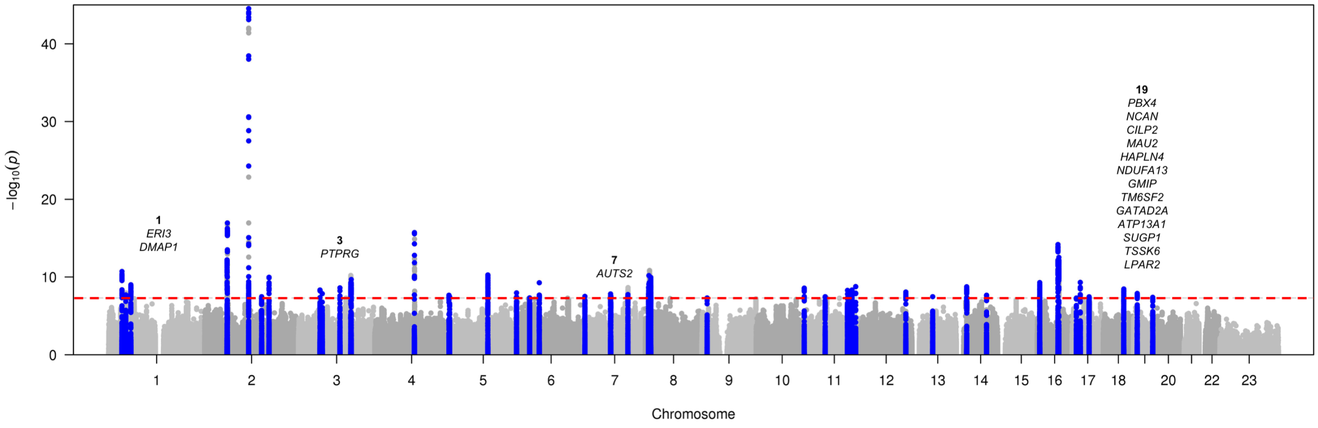

Supplementary Figure 3. Manhattan plots for genome-wide association analysis of sex-stratified sleep duration. Manhattan plots a and b show the -$\log_{10}$ *P* values (y-axis) for all genotyped and imputed SNPs passing quality control in each GWAS, plotted by chromosome (x-axis). Blue peaks represent genome-wide significant loci. Horizontal red line denotes genome-wide significance ($5x10^{-8}$).

a. Men

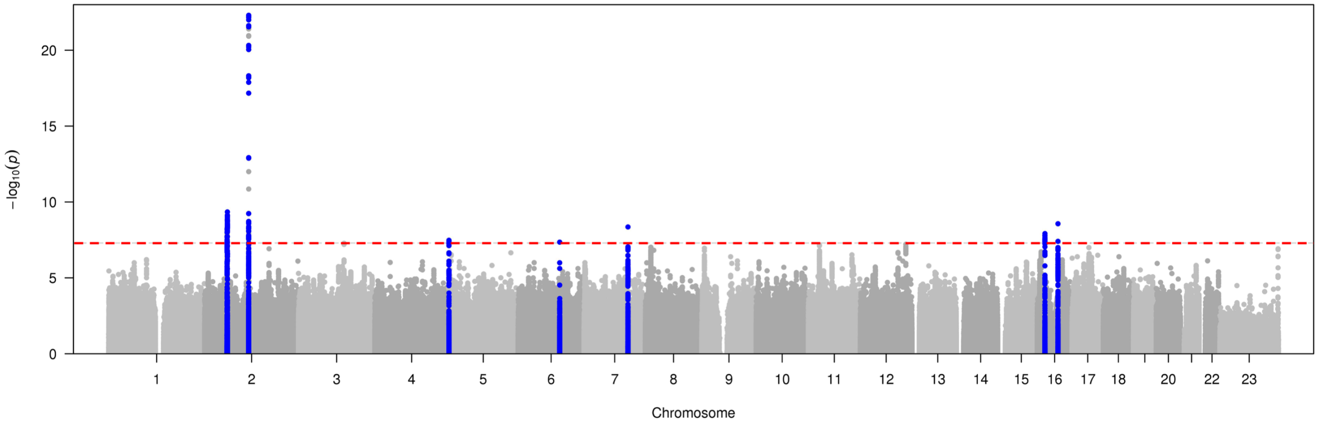

b. Women

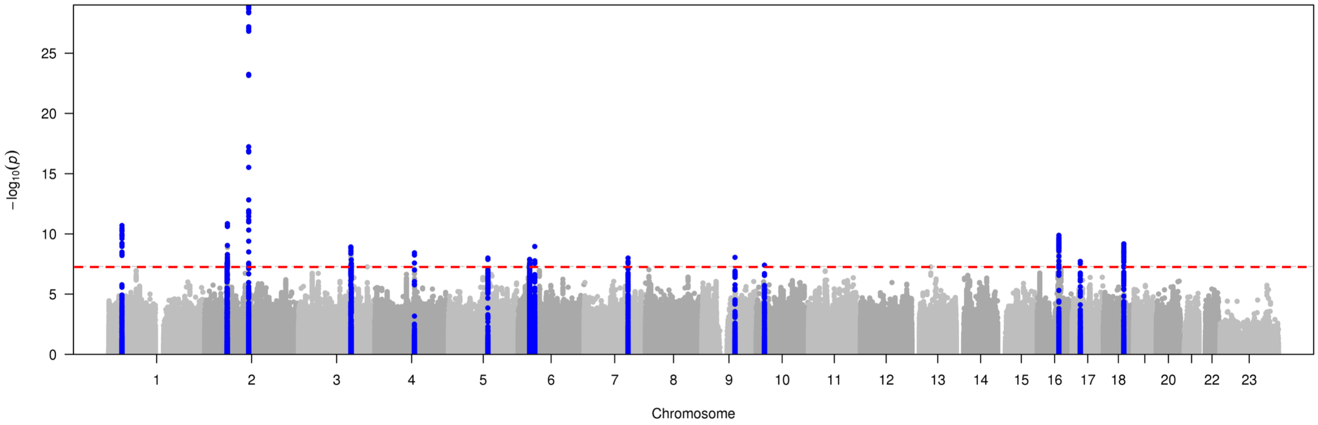

Supplementary Figure 4. Comparison of short and long sleep GWAS effect estimates of 8 long sleep signals. Odds Ratio (OR) reflects the odds of having short/long sleep per each additional effect allele, and error bars indicate 95% CI. Abbreviations: OR=odds ratio; CI=confidence interval. Association effect estimates in this figure can be found in tabular form in **Supplementary Table 5**.

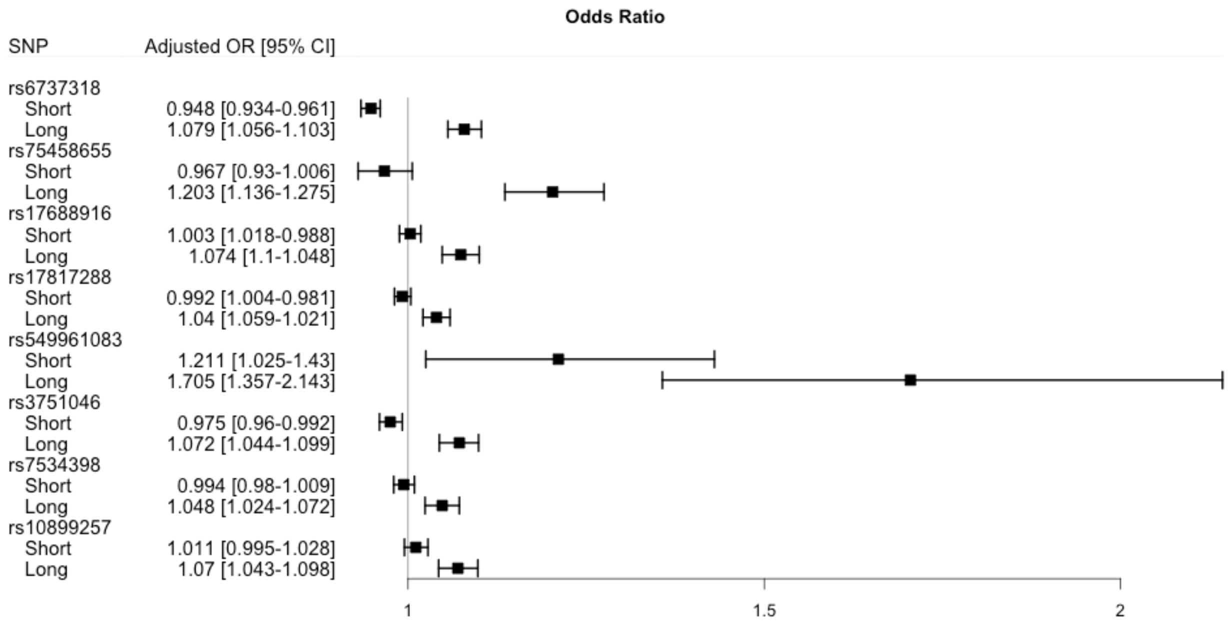

Supplementary Figure 5. Manhattan plots for genome-wide association meta-analyses. Manhattan plots show the $-\log_{10} P$ values (y-axis) for all genotyped and imputed SNPs passing quality control in each GWAS, plotted by chromosome (x-axis). Blue peaks represent additional novel genome-wide significant loci unraveled in meta-analysis. Horizontal red line denotes genome-wide significance (5×10^{-8}). SNPs with high heterogeneity (I^2 >=90) were excluded. Association effect estimates in this figure can be found in tabular form in **Supplementary Data 7**.

a. UK Biobank + CHARGE (adult) (*n* =493,298)

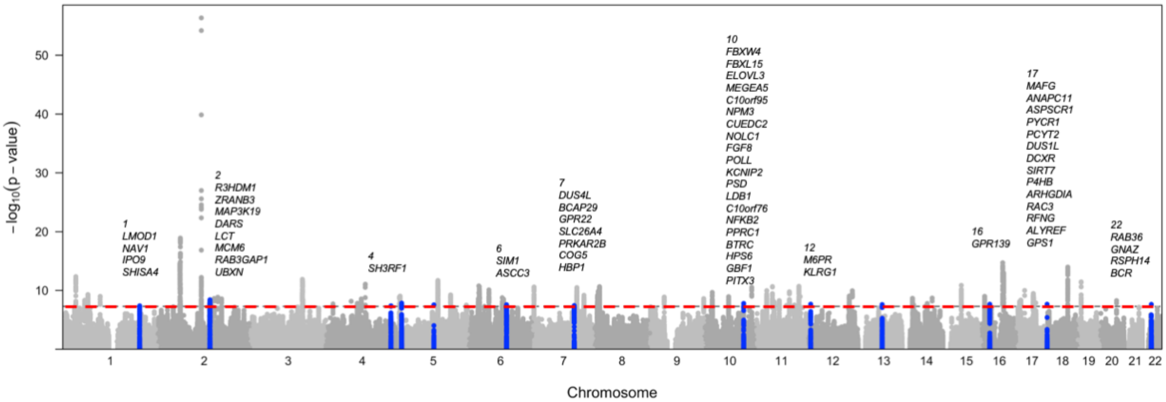

b. UK Biobank + EAGLE (childhood/adolescent) (*n* =456,672)

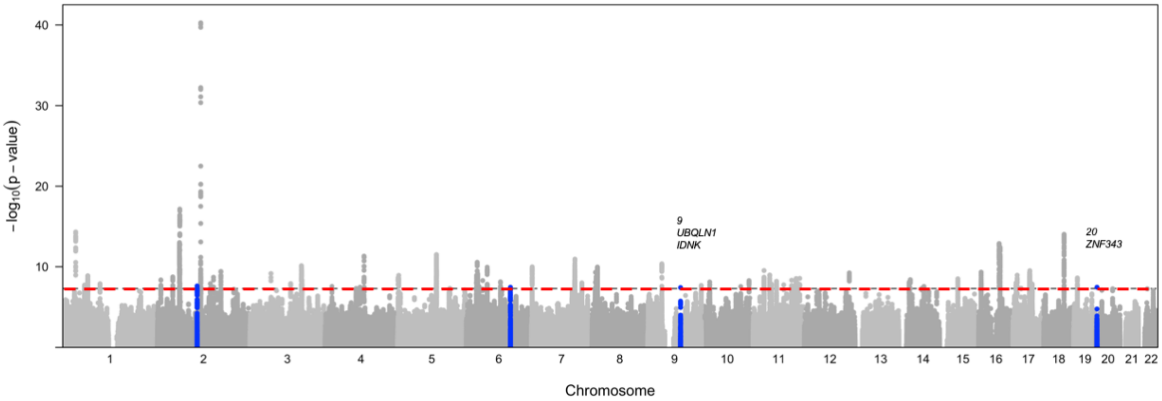

c. UK Biobank + CHARGE + EAGLE (*n* =503,852)

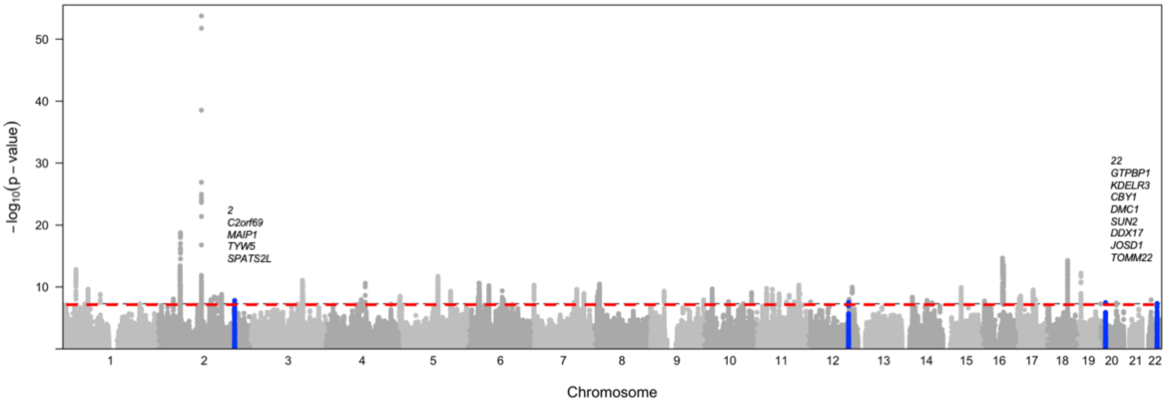

Supplementary Figure 6. Association of sleep loci (7 sleep duration loci, a–g; 5 short sleep loci, h–l; 3 long sleep loci, m–o) with brain imagining phenotypes in the UK Biobank (images from http://big.stats.ox.ac.uk/).

a. Sleep duration (rs7556815) – *PAX8*

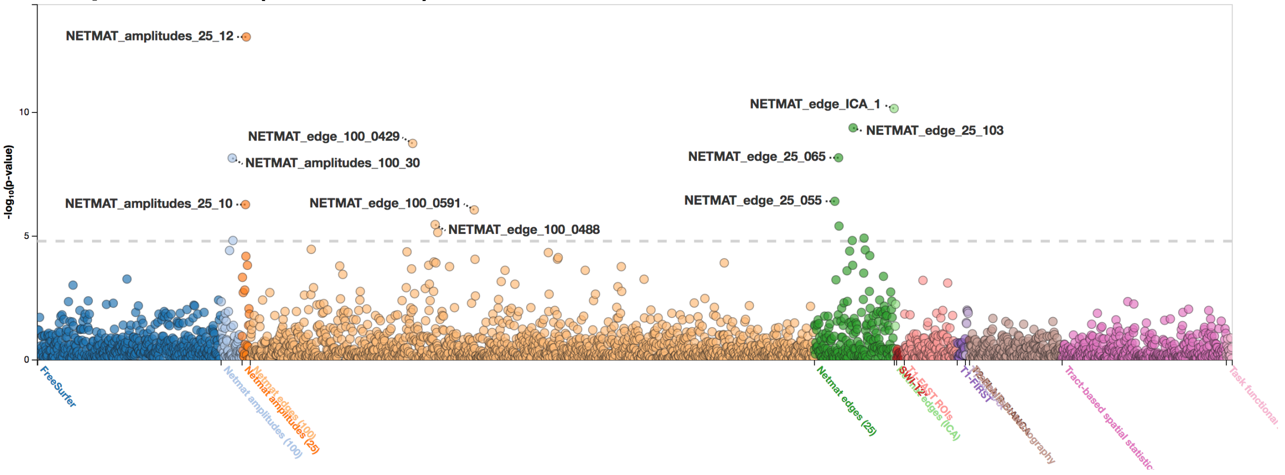

b. Sleep duration (rs13109404) – *BANK1*

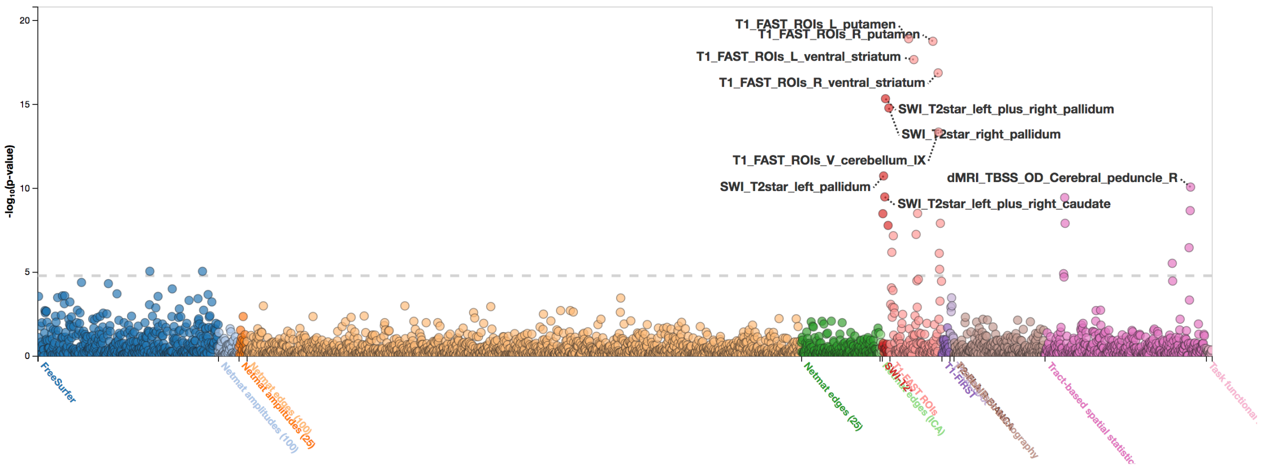

c. Sleep duration (rs330088) – *PPP1R3B*

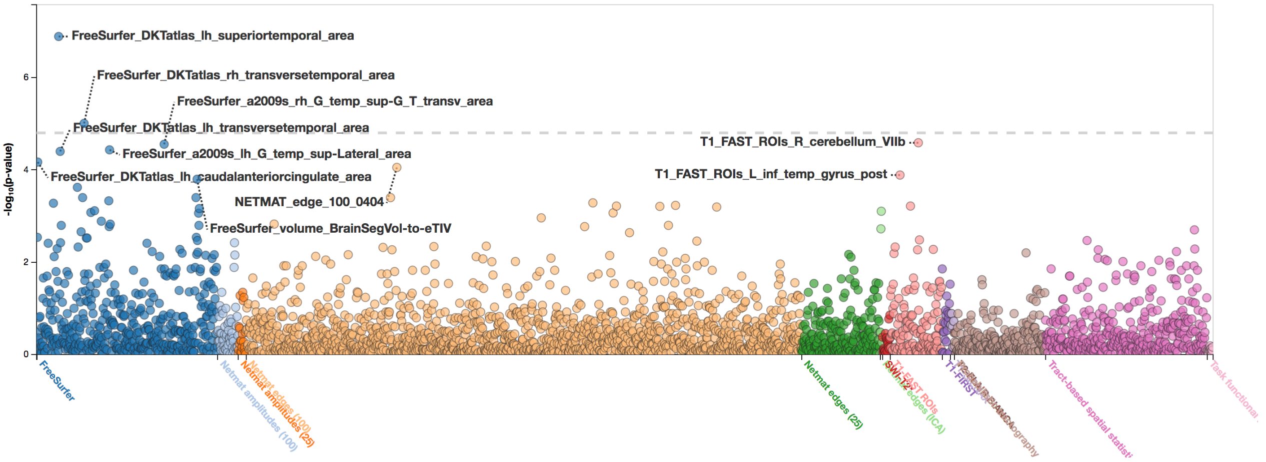

d. Sleep duration (rs11602180) – *PTPRJ*

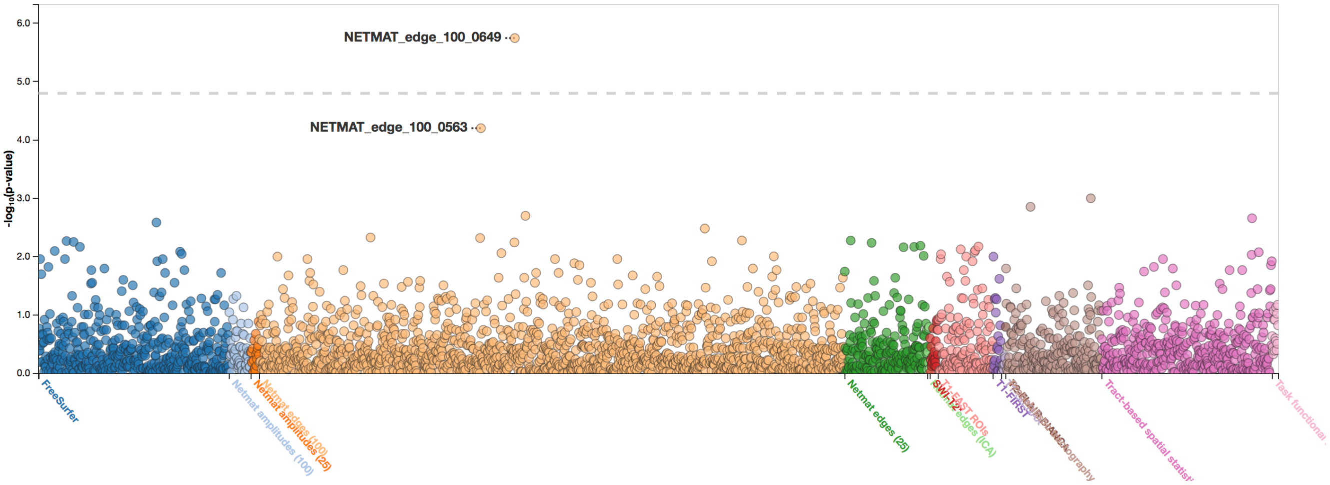

e. Sleep duration (rs9345234) – *EPHA7*

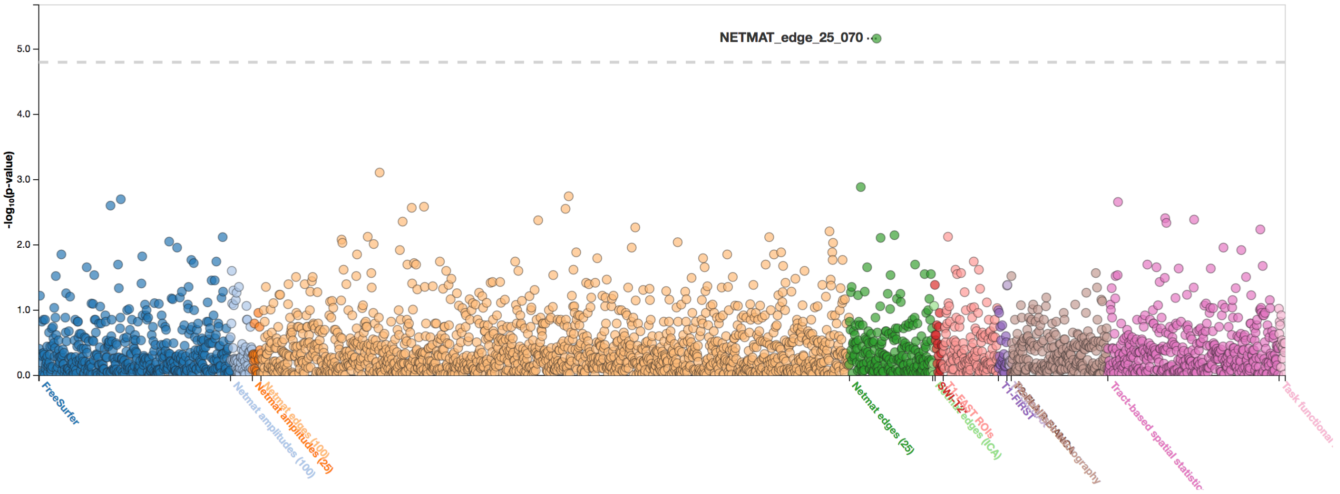

f. Sleep duration (rs174560) – *FADS1/FADS2*

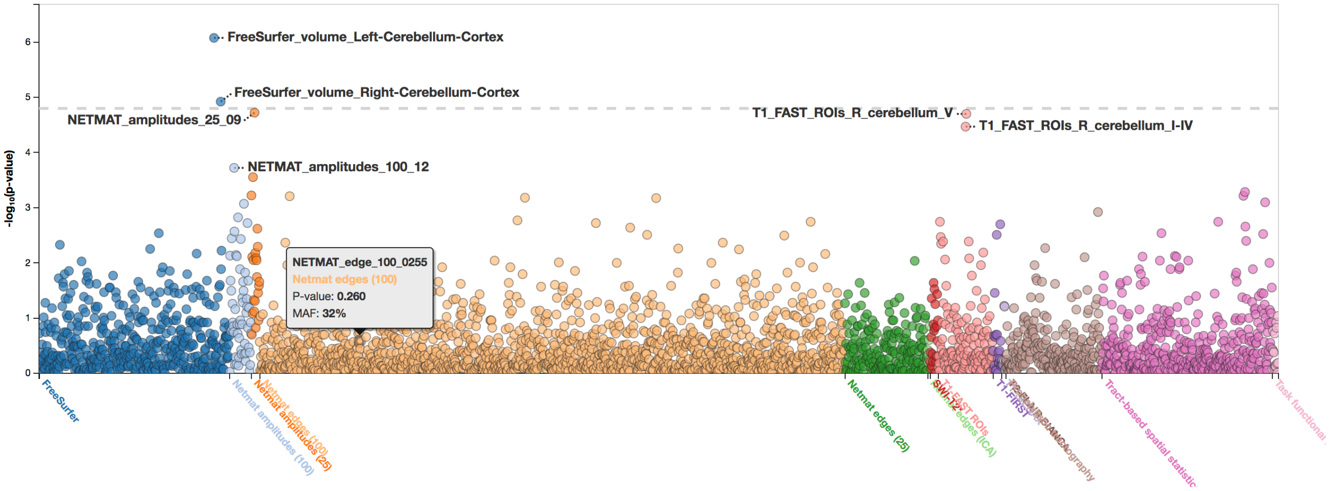

g. Sleep duration (rs12611523) – *SPOPL*

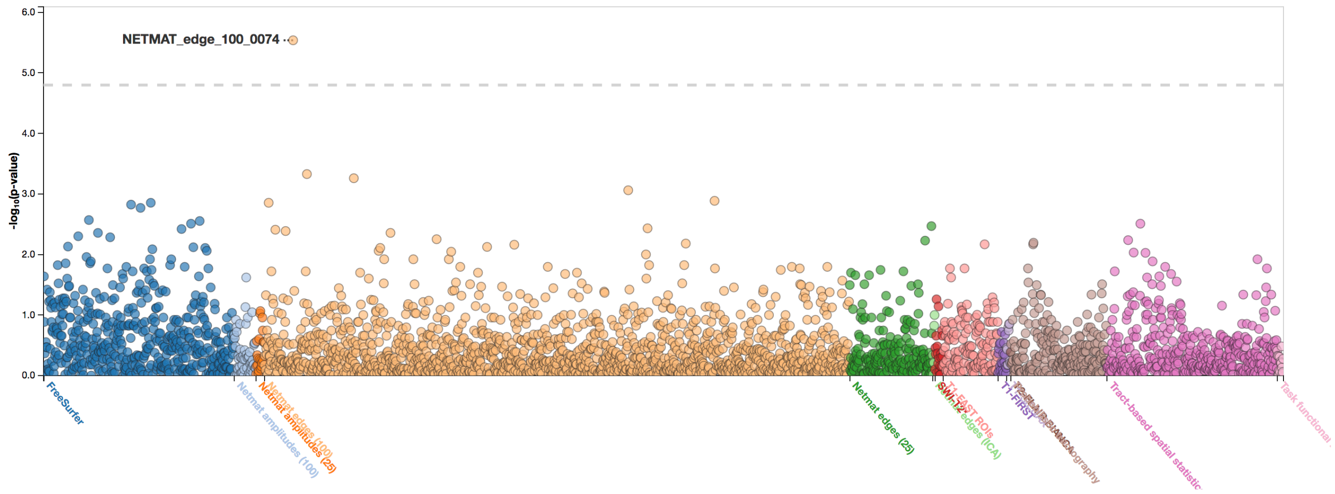

h. Short sleep (rs2863957) – *PAX8*

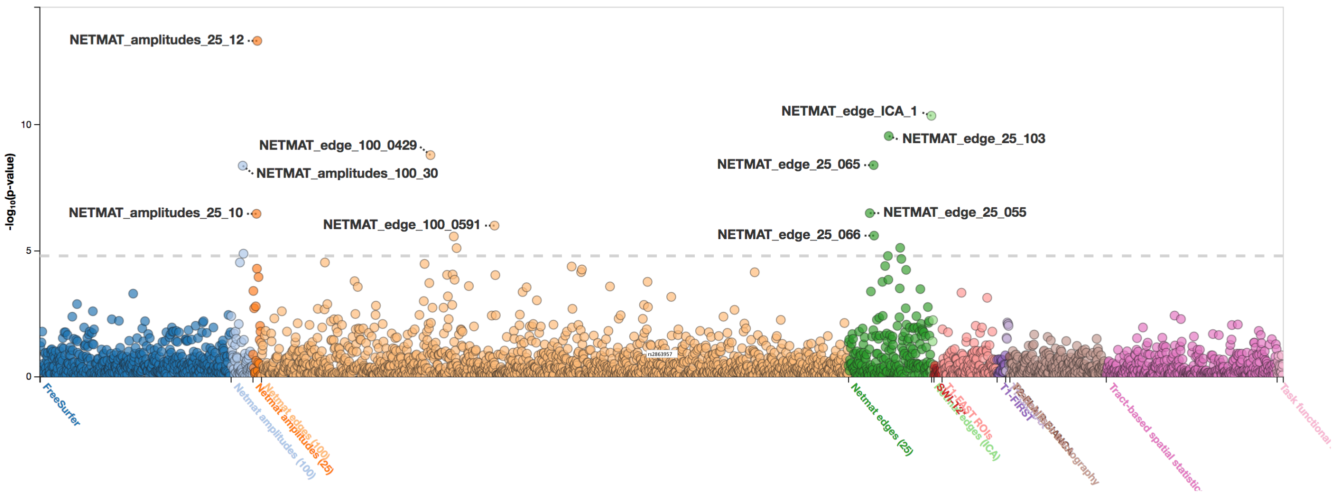

i. Short sleep (rs13107325) – *SLC39A8*

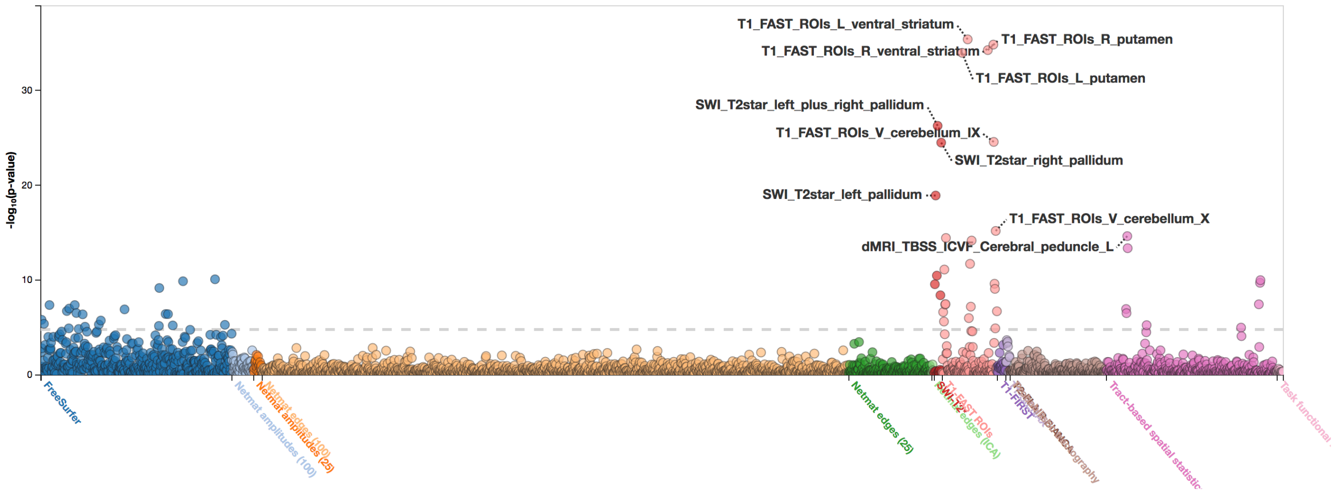

j. Short sleep (rs2820313) – *LMOD1*

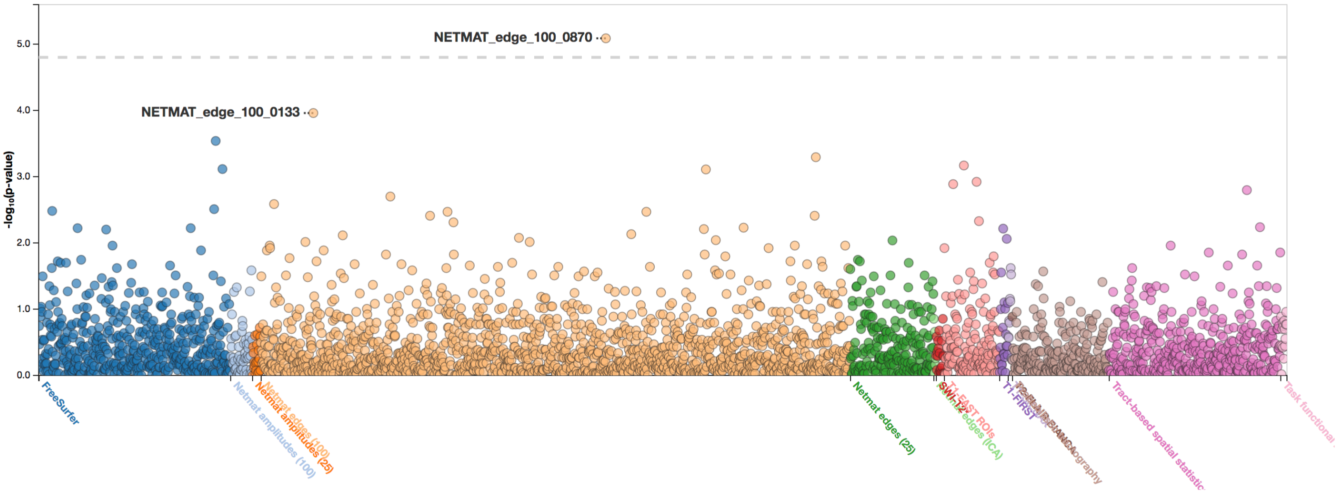

k. Short sleep (rs2186122) – *PDE4B*

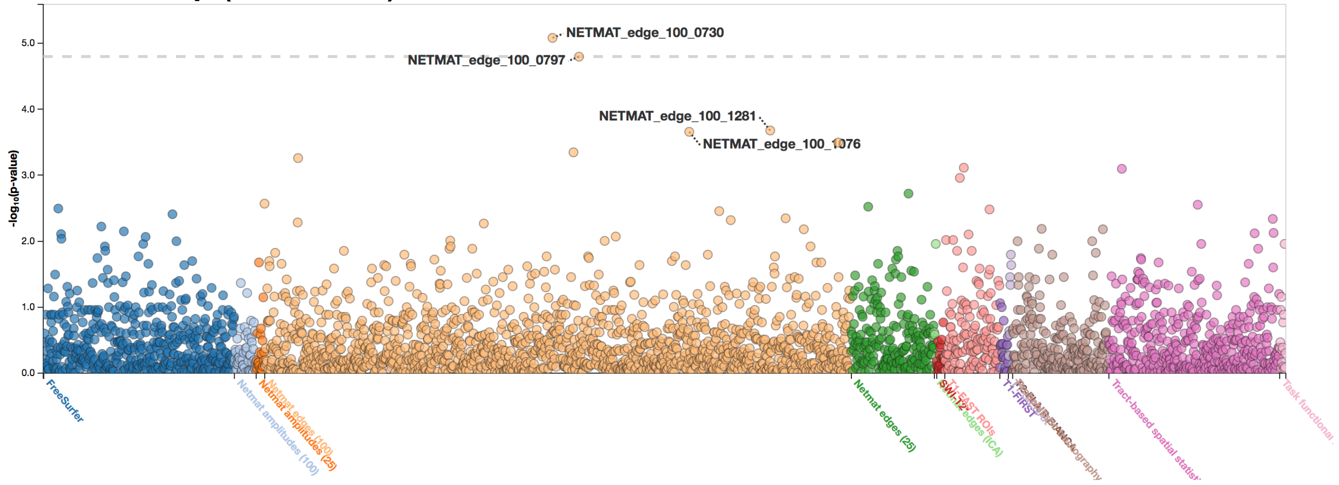

l. Short sleep (rs2014830) – *RBM5*

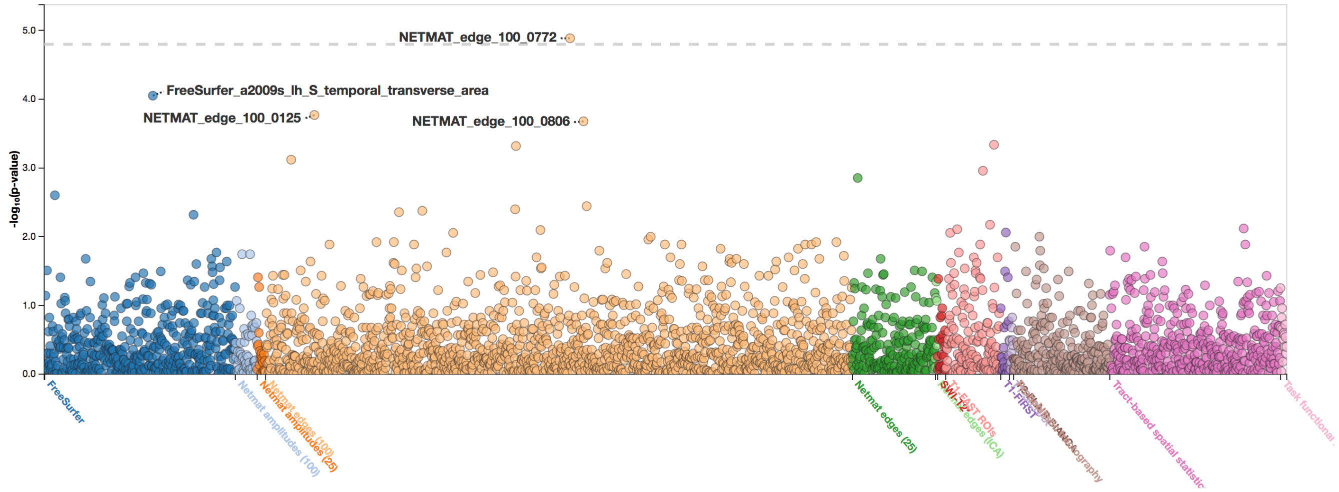

m. Long sleep (rs6737318) – *PAX8*

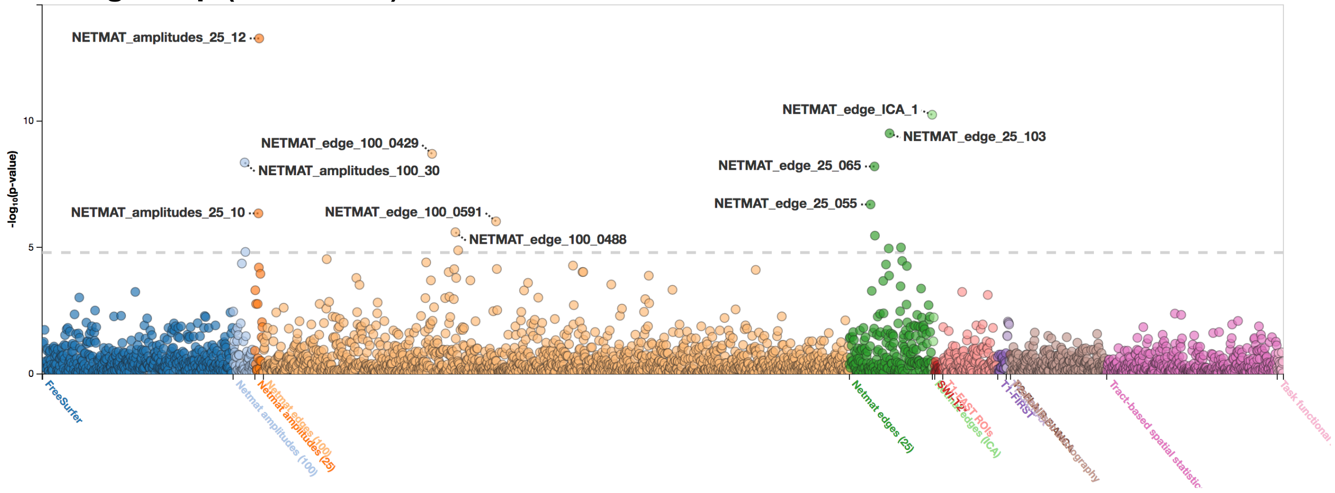

n. Long sleep (rs7534398) – *CAMTA1*

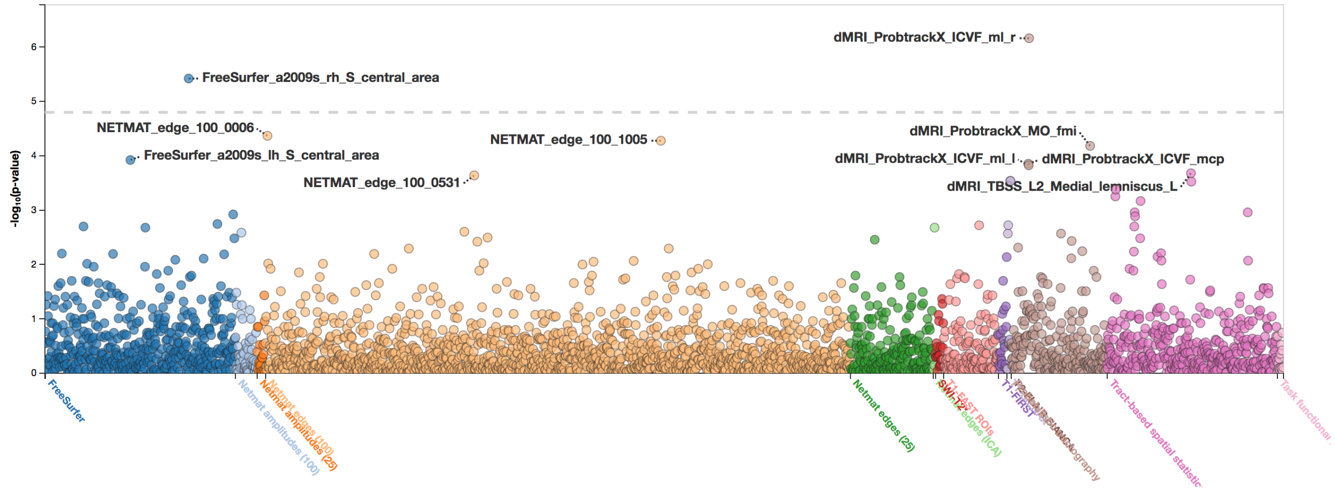

o. Long sleep (rs10899257) – *GUCY2E*

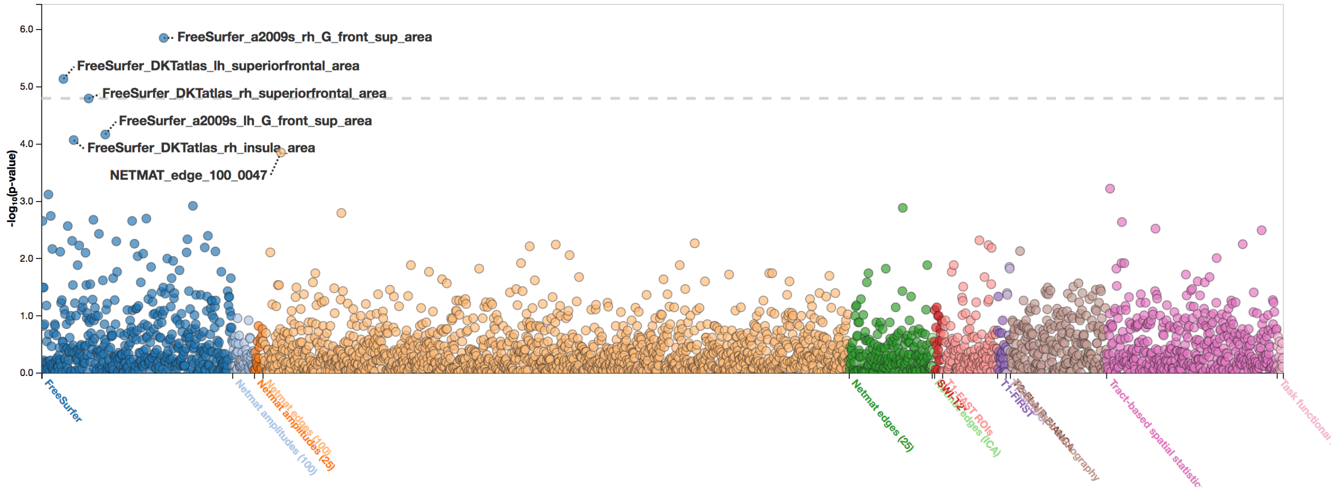

Supplementary Figure 7. Causal relationship of sleep duration with BMI (GIANT: a,b) or type 2 diabetes (DIAGRAM: c,d). Association between single nucleotide polymorphisms (SNP) associated with sleep duration and outcomes (a,c), and lines identify the slopes of the five methods tested. Forest plot shows the estimate of the effect of genetically increased sleep duration (in minutes) on outcomes (kg/m^2 or log OR; b,d). Forest plots show each SNP with the 95% confidence interval (gray line segment; error bars) of the estimate and the Inverse Variance MR, MR-Egger, and Weighted Median MR results in red. MR results can be found in tabular form in **Supplementary Table 13**.

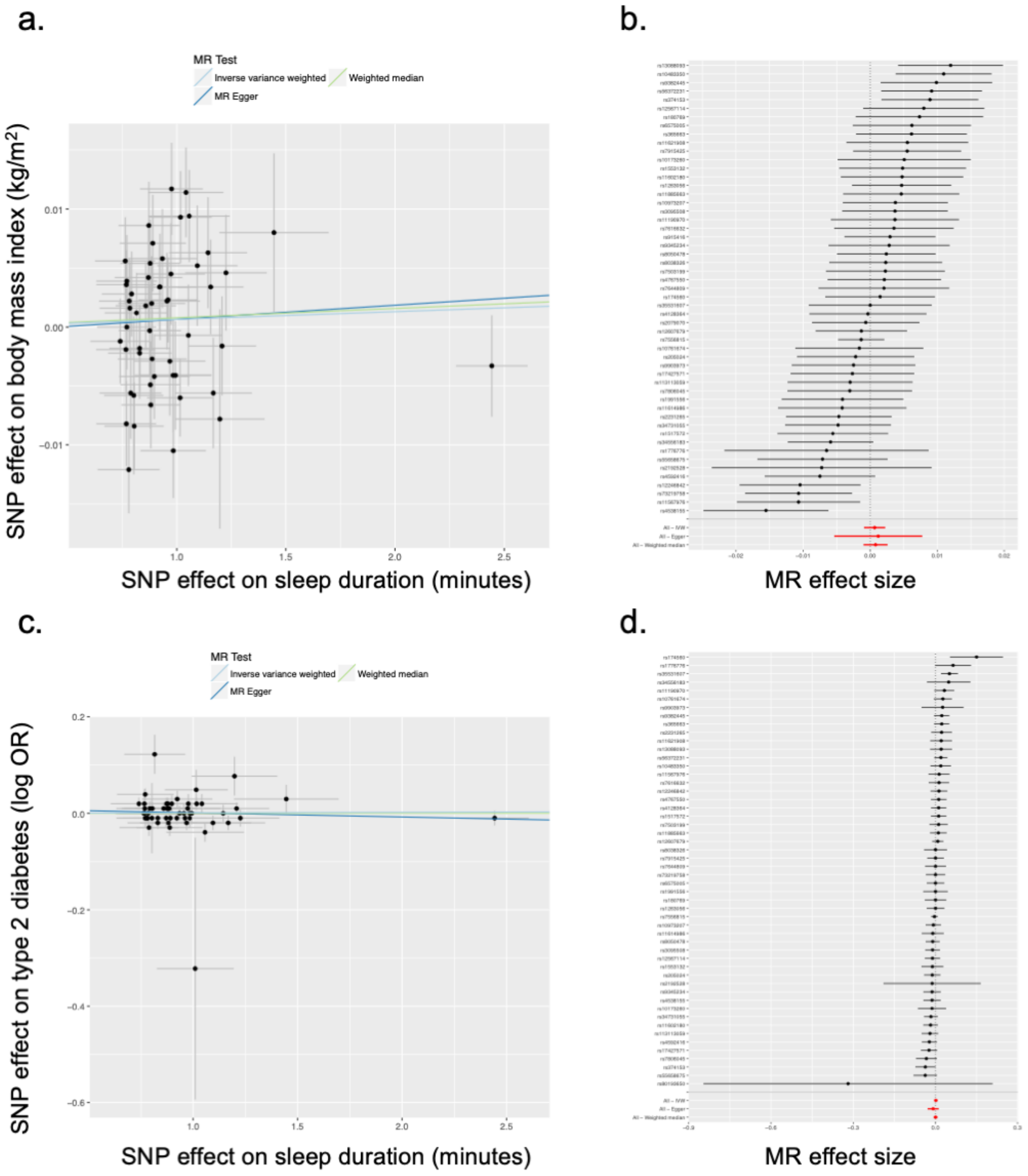

Supplementary Table 1. Descriptive table of UK Biobank participants of European ancestry (*n* =446,118) included in sleep duration genome-wide association study.

	Sleep Duration				
**Characteristic**	All (*N* =446,118)	Short (7h) (*N* =106,192)	Normal (≥7h, <9h) (*N* =305,742)	Long (≥9h) (*N* =34,184)	Phenotypic Correlation *P* value
**Sex (female), %**	54.1	53.1	54.2	56.1	< 2.2e-16
**Age, y**	57.3 (8.0)	57.2 (7.8)	57.1 (8.1)	59.3 (7.9)	< 2.2e-16
**Sleep duration, hours**	7.2 (1.1)	5.7 (0.5)	7.4 (0.5)	9.3 (0.7)	N/A
**BMI, kg/m2**	27.4 (4.7)	27.9 (5.1)	27.1 (4.6)	28.1 (5.1)	< 2.2e-16
**Coffee intake, cups/day**	2.2 (2.0)	2.3 (2.2)	2.1 (1.9)	2.1 (2.1)	< 2.2e-16
**Tea intake, cups/day**	3.5 (2.7)	3.6 (2.9)	3.5 (2.6)	3.7 (2.9)	5.36E-05
**Alcohol intake, g/day**	2.9 (1.5)	3.0 (1.5)	2.8 (1.4)	3.0 (1.6)	< 2.2e-16
**Townsend Deprivation Index**	-2.3 (-3.1 to 0.2)	-2.0 (-3.5 to 0.8)	-2.4 (-3.8 to -0.1)	-2.1 (-3.6 to 0.6)	< 2.2e-16
**Sleep apnea, %**	1.2	1.7	1.0	1.7	< 2.2e-16
**Insomnia symptoms, %**	28.0	51.0	20.7	21.2	< 2.2e-16
**Physical activity, MET-h/week**	28.3 (12.9 to 58.2)	28.3 (12.2 to 61.7)	28.4 (13.2 to 57.1)	26.6 (11.6 to 56.0)	1.98E-03
**Nap during day, %**					< 2.2e-16
Never/Rarely	56.7	58.8	58.2	35.9	
Sometimes	38.1	36.9	37.4	47.5	
Always	5.2	4.2	4.3	16.5	
**Smoking Status, %**					< 2.2e-16
Never/Rarely	53.9	51.4	55.3	50.0	
Current	10.3	12.5	9.3	11.6	
Former	35.5	35.7	35.1	38.0	
**Employment Status, %**					< 2.2e-16
Employed	57.3	60.0	59.1	33.2	
Retired	34.3	30.3	33.8	52.1	
Other	8.4	9.7	7.1	14.3	
**Menopause status, % yes**	33.2	34.4	32.5	35.4	3.68E-03
**Chronic and psychiatric disorders, %**	16.1	18.1	14.3	24.9	< 2.2e-16
**Antidepressant medication, %**	7.1	7.6	5.9	15.8	< 2.2e-16
**Antipsychotic medication, %**	0.5	0.5	0.4	1.8	< 2.2e-16
**Shift Worker, %**					< 2.2e-16
Never/Rarely	47.9	47.4	50.4	26.8	
Sometimes	4.1	5.1	3.9	2.5	
Usually	1.1	1.4	1.0	0.8	
Always	4.2	6.0	3.6	3.1	

Mean ± standard deviation or N(%) or Median [IQR]. P-values derived from chi-squared or anova test. A greater Townsend index score implies a greater degree of deprivation.

Supplementary Table 2. Conditional analyses for sleep duration in the UK Biobank participants of European ancestry using the approximate conditional and joint association method implemented in GCTA unravels additional independent signals.

							Unadjusted for lead signal			Conditioned on lead signal		
**Lead Signal**	**Chr:position (NCBI Build 37)**	**Nearest gene(s)**	**Secondary SNP**	**Chr:position (NCBI Build 37)**	**Alleles (E/A)**	**EAF**	**Beta (min)**	**SE (min)**	***P* Value**	**Beta (min)**	**SE (min)**	***P* Value**
rs75539574	2:58871658	***VRK2***, *LINC01122*	rs2717076	2:58061127	T/C	0.627	1.105	0.140	3.10E-15	1.038	0.141	1.57E-13
rs365663	5:1428883	***SLC6A3***	rs7718889	5:7200526	A/T	0.630	0.768	0.141	5.60E-08	0.776	0.141	3.55E-08
rs7115226	11:113408518	***DRD2***	rs2075654	11:113289066	T/C	0.158	1.092	0.186	6.40E-09	1.020	0.187	4.63E-08
rs1991556	17:44083402	***MAPT***, *STH*, *KANSL1*, *LOC644157*, *LOC644172*	rs9895274	17:45539117	C/T	0.510	0.801	0.136	4.70E-09	0.790	0.136	6.43E-09

Abbreviations: Chr=chromosome, E/A=effect/alternative, position=base pair coordinate hg19, SNP=single nucleotide polymorphism.

Supplementary Table 3. Novel loci identified for sleep duration from genome-wide association study (*P* <5x10-8) for sleep duration with additional exclusions for shift work and prevalent chronic diseases and psychiatric disorders in participants of European ancestry from UK Biobank (*n* =326,224) and corresponding results from the sleep duration GWAS without these exclusions.

						Additional Exclusions			Entire UK Biobank		
**SNP**	**Chr:position (NCBI Build 37)**	**Nearest gene(s)**	**Alleles (E/A)**	**EAF**	**Info**	**Beta (min)**	**SE (min)**	***P* Value**	**Beta (min)**	**SE (min)**	***P* Value**
rs226063	1:44803346	***ERI3***, *DMAP1*	C/T	0.298	1.00	0.929	0.162	**1.10E-08**	0.731	0.148	9.50E-07
rs57316292	3:61792378	***PTPRG***	A/G	0.903	0.99	1.400	0.250	**1.40E-08**	1.159	0.230	3.40E-07
rs41845	7:69025267	***AUTS2***	T/G	0.347	1.00	0.876	0.155	**1.50E-08**	0.725	0.143	4.20E-07
rs6561715	13:53888526	-	A/T	0.630	0.99	0.848	0.154	**3.40E-08**	0.754	0.141	1.10E-07
rs12462757	19:50098423	***PBX4***, *NCAN, CILP2, MAU2, HAPLN4, NDUFA13, GMIP, TM6SF2, GATAD2A, ATP13A1, SUGP1, TSSK6, LPAR2*	G/A	0.833	1.00	1.088	0.198	**4.10E-08**	0.915	0.182	4.10E-07

Genetic association analysis was performed in related subjects of European ancestry using BOLT-LMM linear mixed models and an additive genetic model adjusted for age, sex, 10 principal components of ancestry, genotyping array and genetic correlation matrix [jl2]. Bold denotes genome-wide significant signals (p<5x10-8). Genes indicate all genes within the locus of interest; bold genes indicate nearest gene. Beta (SE) estimates are per each additional effect allele.
Abbreviations: Chr=chromosome, E/A=effect/alternative alleles, EAF=effect allele frequency, Info=imputation quality score, position=base pair coordinate hg19, SNP=single nucleotide polymorphism.

Supplementary Table 4. Genetic correlations between sex-stratified sleep duration GWAS models. Genetic correlation between reported sleep traits are estimated by LDSC.

Trait 1	Trait 2	r_g	SE	z	*P* value	h2_obs	h2_obs_se	h2_int	h2_int_se	gcov_int	gcov_int_se
Sleep duration (men)	Sleep duration (women)	0.989	0.042	23.855	9.00E-126	0.074	0.005	1.012	0.008	0.010	0.006

Supplementary Table 5. Genome-wide association signals (*P* <5x10-8) for long sleep in participants of European ancestry from UK Biobank (*n* =34,184 cases/305,742 controls) and corresponding results from short sleep (*n* =106,192 cases/305,742 controls) and sleep duration (*n* =446,118) GWAS.

						Long Sleep		Short Sleep		Sleep Duration		
**SNP**	**Chr:position (NCBI Build 37)**	**Nearest gene(s)**	**Alleles (E/A)**	**EAF**	**Info**	**OR [95% CI]**	***P* Value**	**OR [95% CI]**	***P* Value**	**Beta**	**SE**	***P* Value**
rs6737318	2:114083120	***PAX8***, *LOC100130100*	G/A	0.222	0.99	1.079 [1.056-1.103]	**3.40E-13**	0.948 [0.934-0.961]	**4.70E-18**	2.442	0.164	**1.60E-49**
rs75458655	11:118115331	***MPZL2***, *JAML, MPZL3*	T/C	0.023	1.00	1.203 [1.136-1.275]	**5.40E-12**	0.967 [0.93-1.006]	2.10E-01	2.064	0.455	5.00E-06
rs17688916	17:43778680	***CRHR1***, *KIAA1267, LOC644246*	T/A	0.796	0.96467	1.074 [1.048-1.1]	**1.10E-11**	1.003 [0.988-1.018]	8.90E-01	0.903	0.172	1.30E-07
rs17817288	16:53807764	***FTO***, *RPGRIP1L*	A/G	0.518	1.00	1.04 [1.021-1.059]	**8.90E-09**	0.992 [0.981-1.004]	9.50E-02	0.829	0.136	**1.20E-09**
rs549961083	5:58184093	*RAB3C, PDE4D*	T/C	0.001	0.868466	1.705 [1.357-2.143]	**9.60E-09**	1.211 [1.025-1.43]	4.80E-04	49.74	8.16	5.00E-01
rs3751046	11:122828342	***BSX***, *C11orf63*	G/A	0.147	0.99	1.072 [1.044-1.099]	**2.00E-08**	0.975 [0.96-0.992]	2.50E-03	1.165	0.192	**1.10E-09**
rs7534398	1:7767464	***CAMTA1***, *VAMP3, PER3, UTS*	A/T	0.201	0.99	1.048 [1.024-1.072]	**2.10E-08**	0.994 [0.98-1.009]	3.50E-01	0.738	0.170	1.20E-05
rs10899257	11:76415209	***GUCY2E***, *LRRC32, TSKU*	A/G	0.144	1.00	1.07 [1.043-1.098]	**4.60E-08**	1.011 [0.995-1.028]	1.80E-01	0.419	0.193	2.70E-02

Genetic association analysis was performed in related subjects of European ancestry using BOLT-LMM linear mixed models and an additive genetic model adjusted for age, sex, 10 principal components of ancestry, genotyping array and genetic correlation matrix [jl2]. OR [95% CI] estimates for short/long sleep are from adjusted PLINK logistic regression analyses. OR [95% CI] or Beta (SE) estimates are per each additional effect allele. Bold denotes genome-wide significant signals (p<5x10-8). Genes indicate all genes within the locus of interest; bold genes indicate nearest gene.

Abbreviations: Chr=chromosome, E/A=effect/alternative alleles, EAF=effect allele frequency, Info=imputation quality score, position=base pair coordinate hg19, SNP=single nucleotide polymorphism.

Supplementary Table 6. Sensitivity analysis for genome-wide significant long sleep loci (*P* <5x10-8) in participants of European ancestry from UK Biobank (n =34,184 cases/305,742 controls), adjusting for known risk factors.

		Baseline Adjustment		BMI Adjustment		Caffeine Adjustment		Additional Lifestyle and Clinical Adjustments	
SNP	Chr:position (NCBI Build 37)	OR [95% CI]	*P* Value	OR [95% CI]	*P* Value	OR [95% CI]	*P* Value	OR [95% CI]	*P* Value
rs6737318	2:114083120	1.079 [1.056-1.103]	3.40E-13	1.08 [1.056-1.104]	2.30E-13	1.079 [1.055-1.103]	3.80E-13	1.082 [1.058-1.106]	1.10E-13
rs75458655	11:118115331	1.203 [1.136-1.275]	5.40E-12	1.204 [1.136-1.276]	5.30E-12	1.203 [1.135-1.274]	6.80E-12	1.201 [1.132-1.274]	3.10E-12
rs17688916	17:43778680	1.074 [1.048-1.1]	1.10E-11	1.074 [1.048-1.1]	9.50E-12	1.074 [1.048-1.1]	9.80E-12	1.059 [1.033-1.085]	1.50E-07
rs17817288	16:53807764	1.04 [1.021-1.059]	8.90E-09	1.053 [1.033-1.072]	4.90E-13	1.039 [1.02-1.059]	1.50E-08	1.047 [1.028-1.067]	7.10E-08
rs549961083	5:58184093	1.705 [1.357-2.143]	9.60E-09	1.708 [1.358-2.148]	8.30E-09	1.707 [1.358-2.145]	8.80E-09	1.78 [1.409-2.248]	7.60E-09
rs3751046	11:122828342	1.072 [1.044-1.099]	2.00E-08	1.073 [1.046-1.101]	1.10E-08	1.071 [1.044-1.099]	2.60E-08	1.074 [1.046-1.103]	2.30E-08
rs7534398	1:7767464	1.048 [1.024-1.072]	2.10E-08	1.047 [1.023-1.071]	4.20E-08	1.048 [1.024-1.072]	2.30E-08	1.05 [1.026-1.075]	1.10E-08
rs10899257	11:76415209	1.07 [1.043-1.098]	4.60E-08	1.071 [1.044-1.099]	3.40E-08	1.07 [1.043-1.098]	4.30E-08	1.072 [1.044-1.1]	3.00E-08

Genetic association analysis was performed in related subjects of European ancestry using BOLT-LMM linear mixed models and an additive genetic model adjusted for age, sex, 10 principal components of ancestry, genotyping array and genetic correlation matrix [jl2] as baseline and additional covariates as indicated for each analysis. OR [95% CI] estimates for short/long sleep are from adjusted PLINK logistic regression analyses. Additional lifestyle and clinical adjustments included BMI, day naps, Townsend index, smoking, alcohol intake, menopause status, employment status, and sleep apnea in addition to baseline adjustments. For further details on sensitivity variables, see methods. All analyses performed in subjects with non-missing covariates. OR [95% CI] estimates are per each additional effect allele. **Abbreviations:** BMI=body mass index, CI=confidence interval, Chr=chromosome, OR=odds ratio, SNP=single nucleotide polymorphism.

Supplementary Table 7. Low genetic correlation between adult (UK Biobank and CHARGE) and childhood/adolescent (EAGLE) genome-wide association studies for sleep duration. Genetic correlation between reported sleep traits are measured by LDSC.

Study 1	Study 2	r_g	SE	z	*P value*	h2_obs	h2_obs_se	h2_int	h2_int_se	gcov_int	gcov_int_se
UK Biobank	CHARGE	1.000	0.123	8.152	3.59E-16	0.037	0.009	1.011	0.006	0.000	0.005
UK Biobank	EAGLE	0.098	0.076	1.293	0.20	0.132	0.044	0.984	0.007	0.003	0.005
CHARGE	EAGLE	0.016	0.180	0.090	0.93	0.130	0.045	0.985	0.007	0.001	0.004

Supplementary Table 8. Descriptive table of UK Biobank participants of European ancestry who had completed up to 7 days of wrist-worn accelerometry (n =85,499).

	Sleep Duration			
**Characteristic**	All (*N*=85,499)	Short (≤ 6.5h) (*N*=13,760)	Normal (>6.5h, ≤8.5h) (*N*=66,110)	Long (>8.5h) (*N*=5,629)
**Sex (female), %**	56.2 (49.6)	44.1 (49.7)	58.5 (49.3)	59.9 (49.0)
**Age, y**	62.5 (7.8)	61.8 (8.0)	62.5 (7.8)	64.2 (7.4)
**BMI, kg/m2**	26.7 (4.5)	27.5 (4.9)	26.5 (4.4)	26.5 (4.5)
**Sleep duration, hours**	7.30 (0.86)	5.93 (0.56)	7.45 (0.51)	8.85 (0.33)
**Sleep duration standard deviation, hours**	0.93 (0.57)	1.30 (0.85)	0.86 (0.47)	0.83 (.42)
**Sleep efficiency, %**	76.18 (7.18)	69.13 (9.05)	77.17 (5.84)	81.84 (4.01)
**Number of sleep bouts, *n***	17.25 (3.59)	16.37 (3.81)	17.38 (3.51)	17.89 (3.57)
**Midpoint of 5-hour daily period of minimum activity (L5 timing), HH:MM**	3:19 (1:06)	3:22 (1:16)	3:19 (1:01)	3:19 (1:06)
**Midpoint of 10-hour daily period of maximum activity (M10 timing), HH:MM**	13:41 (1:13)	13:43 (1:31)	13:41 (1:09)	13:50 (1:05)
**Daytime Inactivity Duration, hours**	0.97 (0.68)	0.82 (0.60)	0.94 (0.63)	1.63 (0.95)
**Sleep Midpoint, HH:MM**	2:59 (0:45)	3:01 (1.07)	3:00 (0:51)	2:49 (0:56)

Abbreviations: SD=standard deviation, BMI=body mass index.

Supplementary Table 9. MAGMA pathway-based enrichment analysis for sleep traits based on MAGMA gene-sets. Top 10 of 10,891 pathways are shown. Significant pathways with P <4.59x10^{-06} for 10,891 tested pathways are bolded.

Trait	Gene Set	N genes	Beta	Beta STD	SE	*P* Value	P_{bon}
Sleep Duration	**GO_bp:go_striatum_development**	**15**	**1.55**	**0.0443**	**0.307**	**2.35E-07**	**0.0026**
	**GO_bp:go_mechanosensory_behavior**	**12**	**1.54**	**0.0393**	**0.313**	**4.81E-07**	**0.0052**
	**GO_mf:go_dopamine_binding**	**10**	**1.61**	**0.0376**	**0.334**	**7.48E-07**	**0.0081**
	**GO_bp:go_subpallium_development**	**21**	**1.14**	**0.0387**	**0.257**	**4.41E-06**	**0.048**
	GO_bp:go_synaptic_signaling	404	0.221	0.0325	0.0539	2.08E-05	0.23
	Curated_gene_sets:ivanova_hematopoiesis_early_progenitor	507	0.186	0.0305	0.0468	3.54E-05	0.39
	GO_bp:go_response_to_auditory_stimulus	23	0.936	0.0332	0.239	4.61E-05	0.50
	GO_bp:go_adenylate_cyclase_activating_g_protein_coupled_receptor_signaling_pathway	71	0.491	0.0305	0.127	5.90E-05	0.64
	GO_cc:go_somatodendritic_compartment	621	0.169	0.0305	0.0441	6.49E-05	0.71
	GO_cc:go_neuron_projection	900	0.141	0.0305	0.0369	6.66E-05	0.72
Short Sleep	Curated_gene_sets:ikeda_mir1_targets_dn	7	1.6	0.0313	0.393	2.34E-05	0.25
	Curated_gene_sets:benporath_oct4_targets	275	0.235	0.0286	0.0607	5.30E-05	0.58
	GO_bp:go_regulation_of_potassium_ion_transport	81	0.443	0.0294	0.114	5.38E-05	0.59
	Curated_gene_sets:kegg_glycolysis_gluconeogenesis	57	0.0421	0.00235	0.133	0.37544	1
	Curated_gene_sets:kegg_citrate_cycle_tca_cycle	29	0.255	0.0102	0.176	0.07381	1
	Curated_gene_sets:kegg_pentose_phosphate_pathway	23	0.0573	0.00203	0.188	0.38029	1
	Curated_gene_sets:kegg_pentose_and_glucuronate_interconversions	29	0.198	0.00789	0.238	0.20214	1
	Curated_gene_sets:kegg_fructose_and_mannose_metabolism	32	-0.173	-0.00723	0.163	0.85546	1
	Curated_gene_sets:kegg_galactose_metabolism	26	0.116	0.00435	0.193	0.27453	1
	Curated_gene_sets:kegg_ascorbate_and_aldarate_metabolism	26	-0.288	-0.0108	0.267	0.85941	1
Long Sleep	**GO_bp:go_catecholamine_metabolic_process**	**36**	**0.69**	**0.0306**	**0.146**	**1.20E-06**	**0.013**
	**GO_bp:go_catechol_containing_compound_metabolic_process**	**36**	**0.69**	**0.0306**	**0.146**	**1.20E-06**	**0.013**
	GO_bp:go_catecholamine_biosynthetic_process	17	0.92	0.028	0.226	2.30E-05	0.25
	GO_bp:go_catechol_containing_compound_biosynthetic_process	17	0.92	0.028	0.226	2.30E-05	0.25
	GO_bp:go_dopamine_metabolic_process	20	0.773	0.0255	0.191	2.61E-05	0.28
	GO_bp:go_phenol_containing_compound_biosynthetic_process	31	0.641	0.0263	0.165	5.10E-05	0.56
	Curated_gene_sets:reactome_microrna_mirna_biogenesis	22	0.788	0.0273	0.206	6.50E-05	0.71
	Curated_gene_sets:kurokawa_liver_cancer_chemotherapy_dn	39	0.508	0.0234	0.136	8.87E-05	0.97
	Curated_gene_sets:kegg_glycolysis_gluconeogenesis	57	0.00534	0.000298	0.122	0.48255	1
	Curated_gene_sets:kegg_citrate_cycle_tca_cycle	29	0.0386	0.00153	0.162	0.40604	1

Supplementary Table 10. Pascal pathway-based enrichment test for sleep traits using Pascal (gene-set enrichment analysis using 1,077 pathways from KEGG, REACTOME, BIOCARTA databases). Top 10 of 1,077 pathways are shown. Significant pathways with *P* <4.64x10^{-05} for 1,077 tested pathways are bolded.

Trait	Name	chi2Pvalue	empPvalue	P_{bon}
Sleep Duration	**KEGG_LONG_TERM_DEPRESSION**	**1.66E-06**	**1.00E-06**	**1.08E-03**
	**REACTOME_NEURONAL_SYSTEM**	**6.80E-07**	**1.60E-06**	**1.72E-03**
	**REACTOME_TRANSMISSION_ACROSS_CHEMICAL_SYNAPSES**	**4.12E-06**	**5.20E-06**	**5.60E-03**
	KEGG_MAPK_SIGNALING_PATHWAY	2.36E-05	1.02E-04	1.10E-01
	KEGG_GAP_JUNCTION	1.34E-04	1.30E-04	1.40E-01
	REACTOME_HEMOSTASIS	4.13E-04	5.20E-04	5.60E-01
	REACTOME_SIGNALING_BY_FGFR_IN_DISEASE	2.51E-04	5.57E-04	6.00E-01
	REACTOME_SIGNALING_BY_FGFR	4.42E-04	7.10E-04	7.65E-01
	REACTOME_MEIOTIC_SYNAPSIS	1.01E-03	9.00E-04	9.69E-01
	REACTOME_PLC_BETA_MEDIATED_EVENTS	9.76E-04	1.06E-03	1.00
Short Sleep	**REACTOME_DEVELOPMENTAL_BIOLOGY**	**3.17E-05**	**2.03E-05**	**2.19E-02**
	**KEGG_LONG_TERM_DEPRESSION**	**1.29E-05**	**2.18E-05**	**2.35E-02**
	**KEGG_MAPK_SIGNALING_PATHWAY**	**1.18E-05**	**2.42E-05**	**2.61E-02**
	REACTOME_SIGNALING_BY_FGFR_IN_DISEASE	3.74E-05	5.39E-05	5.81E-02
	REACTOME_SIGNALING_BY_FGFR	3.84E-05	5.70E-05	6.14E-02
	REACTOME_DOWNSTREAM_SIGNALING_OF_ACTIVATED_FGFR	1.99E-04	2.26E-04	2.43E-01
	REACTOME_PROLONGED_ERK_ACTIVATION_EVENTS	1.32E-04	2.34E-04	2.52E-01
	KEGG_NON_SMALL_CELL_LUNG_CANCER	1.35E-04	2.37E-04	2.55E-01
	REACTOME_MAP_KINASE_ACTIVATION_IN_TLR_CASCADE	1.91E-04	2.61E-04	2.81E-01
	REACTOME_FRS2_MEDIATED_CASCADE	3.15E-04	2.95E-04	3.18E-01
Long Sleep	KEGG_LONG_TERM_DEPRESSION	3.33E-04	2.94E-04	3.17E-01
	REACTOME_TRAFFICKING_OF_GLUR2_CONTAINING_AMPA_RECEPTORS	6.57E-04	7.60E-04	8.19E-01
	REACTOME_NEURONAL_SYSTEM	4.38E-04	7.80E-04	8.40E-01
	BIOCARTA_P35ALZHEIMERS_PATHWAY	2.85E-03	8.90E-04	9.59E-01
	REACTOME_TRANSMISSION_ACROSS_CHEMICAL_SYNAPSES	7.69E-04	1.02E-03	1.00
	BIOCARTA_BIOPEPTIDES_PATHWAY	5.24E-03	1.78E-03	1.00
	KEGG_MAPK_SIGNALING_PATHWAY	2.48E-03	3.14E-03	1.00
	REACTOME_CASPASE_MEDIATED_CLEAVAGE_OF_CYTOSKELETAL_PROTEINS	1.92E-02	3.35E-03	1.00
	REACTOME_TRAFFICKING_OF_AMPA_RECEPTORS	7.39E-03	3.55E-03	1.00
	REACTOME_NEUROTRANSMITTER_RECEPTOR_BINDING_AND_DOWNSTREAM _TRANSMISSION_IN_THE_POSTSYNAPTIC_CELL	4.27E-03	3.67E-03	1.00

Supplementary Table 11. Enrichment analysis of synaptic sleep-need-index phosphoproteins (SNIPPs) human orthologs in sleep duration GWAS.

				Sleep Duration		Short Sleep		Long Sleep	
**Gene Symbol**	**Chromosome**	**Start**	**End**	**# SNPs**	***P* value**	**# SNPs**	***P* value**	**# SNPs**	***P* value**
SCN1A	Chr 2	166845669	167005642	**593**	**1.92E-09**	593	4.40E-03	**593**	**2.03E-04**
PDE4B	Chr 1	66258192	66840262	1125	4.29E-09	**1125**	**7.91E-08**	1125	1.56E-01
GRM5	Chr 11	88237743	88799113	1107	3.01E-06	1107	6.24E-05	1107	2.28E-01
MAP2	Chr 2	210288770	210598834	604	9.75E-06	604	3.06E-03	604	4.26E-02
MINK1	Chr 17	4736634	4801356	337	3.59E-05	337	6.62E-03	337	6.76E-03
NAV1	Chr 1	201617449	201796102	459	8.70E-05	459	2.77E-06	459	4.74E-02
PITPNM2	Chr 12	123468026	123635285	170	1.44E-04	170	5.07E-05	170	6.97E-01
SHANK2	Chr 11	70313960	70935842	1234	5.55E-04	1234	5.35E-03	1234	2.99E-01
RIMS1	Chr 6	72596405	73112845	1139	6.93E-04	1139	1.07E-01	1139	5.17E-01
USP31	Chr 16	23072727	23160591	366	8.46E-04	366	1.10E-01	366	8.44E-02
SCN2A	Chr 2	166095911	166248820	491	1.05E-03	491	2.62E-02	491	6.84E-02
ARFGAP2	Chr 11	47185848	47198676	137	1.20E-03	137	4.93E-03	137	1.15E-01
LRRC7	Chr 1	70032867	70589171	1350	1.97E-03	1350	6.55E-02	1350	1.51E-01
SRCIN1	Chr 17	36686258	36762183	325	1.99E-03	325	1.10E-01	325	1.41E-01
ELFN2	Chr 22	37763999	37823505	367	2.01E-03	367	1.33E-02	367	6.12E-01
SHANK3	Chr 22	51113069	51171640	230	3.06E-03	230	7.00E-01	230	9.92E-03
PCLO	Chr 7	82383320	82792197	992	3.30E-03	992	1.06E-01	992	7.01E-02
ARHGEF2	Chr 1	155916629	155959864	138	3.45E-03	138	6.13E-03	138	8.52E-01
AGAP2	Chr 12	58118075	58135944	131	1.05E-02	131	5.13E-02	131	2.10E-01
DLGAP3	Chr 1	35331036	35370984	229	1.19E-02	229	4.34E-01	229	3.31E-01
DLGAP2	Chr 8	1449531	1656642	997	1.53E-02	997	4.07E-03	997	3.27E-01
GRIN2B	Chr 12	13714409	14133022	1141	1.80E-02	1141	3.14E-01	1141	4.69E-01
BAIAP2	Chr 17	79008946	79091232	599	1.85E-02	599	1.95E-01	599	9.55E-02
SIPA1L1	Chr 14	71933901	72206120	292	2.17E-02	292	7.79E-02	292	3.72E-01
SORBS2	Chr 4	186506597	186877870	1522	2.99E-02	1522	1.35E-01	1522	8.46E-01
MARK4	Chr 19	45754515	45808541	326	3.20E-02	326	2.18E-01	326	1.64E-01
TNIK	Chr 3	170780291	171178197	993	3.25E-02	993	1.10E-02	993	9.55E-01
DMXL2	Chr 15	51739920	51914967	351	3.64E-02	351	4.23E-03	351	1.17E-01
ARHGAP39	Chr 8	145754562	145911194	116	4.05E-02	116	1.28E-01	116	4.83E-03
MARK2	Chr 11	63606399	63678492	214	6.26E-02	214	7.56E-02	214	6.58E-01
RAPGEF2	Chr 4	160188997	160281301	246	7.30E-02	246	2.16E-02	246	4.37E-01
CAMK2B	Chr 7	44256748	44365230	393	7.42E-02	393	1.58E-01	393	7.69E-01
LPPR4	Chr 1	99729847	99775138	226	7.96E-02	226	9.62E-03	226	6.63E-01
ANK3	Chr 10	61786055	62493284	1869	8.75E-02	1869	3.16E-02	1869	1.97E-02
RIMS2	Chr 8	104512975	105266656	2182	1.11E-01	2182	5.58E-01	2182	5.16E-01
DOCK7	Chr 1	62920396	63154039	330	1.41E-01	330	7.78E-01	330	6.56E-01
BSN	Chr 3	49591921	49708982	233	1.66E-01	233	4.36E-02	233	8.69E-01
MARK1	Chr 1	220701567	220837799	255	1.69E-01	255	5.34E-02	255	6.04E-01
MFF	Chr 2	228189866	228222552	381	1.75E-01	381	1.37E-01	381	2.28E-01
IQSEC1	Chr 3	12938541	13114617	656	1.75E-01	656	1.17E-02	656	1.33E-01
CEP170	Chr 1	243287729	243418708	170	1.87E-01	170	1.29E-01	170	4.70E-02

ANKRD63	Chr 15	40573644	40574787	202	1.87E-01	202	8.03E-01	202	1.63E-01
SYNPO	Chr 5	149980641	150038792	153	2.04E-01	153	2.27E-01	153	4.48E-01
C2CD4C	Chr 19	405442	409170	258	2.18E-01	258	3.39E-01	258	7.47E-01
TBC1D10B	Chr 16	30368421	30381522	63	2.38E-01	63	2.79E-01	63	9.47E-01
MAST1	Chr 19	12949258	12985766	121	2.64E-01	121	2.97E-01	121	1.39E-01
TANC2	Chr 17	61086897	61505067	380	2.93E-01	380	4.00E-01	380	5.47E-02
DNM1	Chr 9	130965662	131017527	213	0.31	213	0.052	213	0.55
RAP1GAP2	Chr 17	2699731	2941035	902	3.21E-01	902	4.51E-01	902	5.62E-02
BRSK1	Chr 19	55793439	55823903	236	3.25E-01	236	5.20E-01	236	7.89E-01
ANKS1B	Chr 12	99128568	100378432	2392	3.82E-01	2392	1.10E-01	2392	2.73E-01
OSBPL6	Chr 2	179059207	179264160	515	4.05E-01	515	4.09E-01	515	4.51E-01
CASKIN1	Chr 16	2227183	2246465	140	4.09E-01	140	8.71E-02	140	8.54E-01
RAP1GAP	Chr 1	21922707	21995856	456	4.27E-01	456	3.62E-01	456	5.02E-01
ARHGAP21	Chr 10	24872537	25012597	378	4.61E-01	378	8.37E-01	378	5.92E-01
CAMSAP1	Chr 9	138700332	138799005	332	0.47	332	0.59	332	0.29
EXOC1	Chr 4	56719815	56771244	240	4.95E-01	240	7.01E-01	240	2.36E-01
CADPS	Chr 3	62384020	62861064	1363	5.58E-01	1363	4.51E-01	1363	4.51E-01
ABI1	Chr 10	27035524	27150016	521	5.76E-01	521	4.51E-02	521	2.48E-01
CACNA1E	Chr 1	181452685	181775921	809	6.31E-01	809	6.03E-02	809	3.30E-01
ARFGAP3	Chr 22	43192531	43253408	405	6.71E-01	405	7.45E-01	405	8.93E-01
GPRIN1	Chr 5	176022802	176037131	211	6.96E-01	211	7.72E-01	211	2.14E-01
CLASP2	Chr 3	33537738	33759705	368	7.29E-01	368	1.05E-01	368	3.16E-01
ABL2	Chr 1	179068461	179198819	423	7.77E-01	423	6.50E-01	423	1.31E-01
ILDR2	Chr 1	166882440	166944561	190	8.15E-01	190	7.75E-01	190	1.74E-01
STK32C	Chr 10	134020995	134146063	620	8.27E-01	620	5.48E-01	620	3.91E-01
RAB11FIP5	Chr 2	73300509	73340146	299	9.23E-01	299	8.64E-01	299	5.86E-01
CLASP1	Chr 2	122095351	122407052	543	9.23E-01	543	8.61E-01	543	6.03E-01
TRIO	Chr 5	14143828	14509458	865	9.54E-01	865	8.04E-01	865	9.43E-01

Supplementary Table 12. Tissue enrichment analysis of gene expression in GTEx RNA-seq data using MAGMA. Significant tissue enrichments with P <9.43x10^{-04} for 53 tested tissues are bolded.

	Sleep Duration			Short Sleep			Long Sleep		
**Tissue**	**Beta**	**SE**	***P* Value**	**Beta**	**SE**	***P* Value**	**Beta**	**SE**	***P* Value**
Brain_Cerebellar_Hemisphere	0.0556	0.0089	**2.21E-10**	0.0388	0.0079	**5.27E-07**	0.0265	0.0071	**9.81E-05**
Brain_Cerebellum	0.0559	0.0093	**8.22E-10**	0.0384	0.0082	**1.61E-06**	0.0261	0.0074	**1.99E-04**
Brain_Frontal_Cortex_BA9	0.0593	0.0101	**2.47E-09**	0.0343	0.0090	**6.91E-05**	0.0208	0.0080	4.76E-03
Brain_Cortex	0.0581	0.0104	**1.27E-08**	0.0334	0.0092	**1.49E-04**	0.0197	0.0082	8.15E-03
Brain_Anterior_cingulate_cortex_BA24	0.0584	0.0108	**2.84E-08**	0.0326	0.0095	**3.13E-04**	0.0173	0.0085	2.10E-02
Brain_Nucleus_accumbens_basal_ganglia	0.0573	0.0112	**1.55E-07**	0.0325	0.0099	**5.17E-04**	0.0070	0.0088	2.14E-01
Brain_Caudate_basal_ganglia	0.0529	0.0118	**3.62E-06**	0.0310	0.0104	1.43E-03	0.0051	0.0092	2.89E-01
Brain_Hippocampus	0.0526	0.0120	**6.17E-06**	0.0316	0.0106	1.44E-03	0.0113	0.0094	1.14E-01
Brain_Hypothalamus	0.0525	0.0122	**8.61E-06**	0.0309	0.0107	2.00E-03	0.0055	0.0095	2.80E-01
Brain_Putamen_basal_ganglia	0.0491	0.0119	**1.77E-05**	0.0282	0.0105	3.52E-03	0.0036	0.0093	3.48E-01
Brain_Amygdala	0.0467	0.0120	**4.72E-05**	0.0270	0.0106	5.33E-03	0.0074	0.0094	2.16E-01
Pituitary	0.0446	0.0135	**5.03E-04**	0.0252	0.0118	1.61E-02	0.0156	0.0102	6.39E-02
Brain_Substantia_nigra	0.0348	0.0133	4.47E-03	0.0176	0.0117	6.59E-02	0.0040	0.0103	3.47E-01
Testis	0.0150	0.0077	2.66E-02	0.0118	0.0069	4.27E-02	0.0035	0.0062	2.84E-01
Brain_Spinal_cord_cervical_c.1	0.0155	0.0131	1.20E-01	0.0179	0.0115	5.99E-02	0.0032	0.0102	3.78E-01
Cells_EBV.transformed_lymphocytes	0.0069	0.0073	1.71E-01	0.0008	0.0065	4.50E-01	0.0122	0.0059	1.93E-02
Cells_Transformed_fibroblasts	0.0015	0.0108	4.47E-01	0.0026	0.0096	3.94E-01	0.0020	0.0085	4.07E-01
Ovary	-0.0021	0.0152	5.55E-01	0.0224	0.0131	4.33E-02	-0.0055	0.0114	6.85E-01
Uterus	-0.0136	0.0191	7.61E-01	-0.0013	0.0162	5.33E-01	-0.0041	0.0137	6.17E-01
Colon_Sigmoid	-0.0153	0.0209	7.68E-01	-0.0088	0.0176	6.91E-01	-0.0171	0.0148	8.75E-01
Muscle_Skeletal	-0.0076	0.0100	7.77E-01	-0.0117	0.0089	9.05E-01	0.0029	0.0080	3.61E-01
Whole_Blood	-0.0080	0.0080	8.40E-01	-0.0084	0.0072	8.79E-01	0.0013	0.0066	4.20E-01
Cervix_Endocervix	-0.0211	0.0208	8.44E-01	-0.0165	0.0175	8.27E-01	-0.0107	0.0146	7.68E-01
Artery_Tibial	-0.0236	0.0163	9.27E-01	-0.0138	0.0140	8.38E-01	-0.0189	0.0121	9.40E-01
Nerve_Tibial	-0.0266	0.0179	9.31E-01	-0.0057	0.0152	6.45E-01	-0.0076	0.0128	7.23E-01
Esophagus_Muscularis	-0.0314	0.0208	9.34E-01	-0.0252	0.0176	9.24E-01	-0.0098	0.0148	7.46E-01
Esophagus_Gastroesophageal_Junction	-0.0360	0.0221	9.49E-01	-0.0236	0.0186	8.98E-01	-0.0188	0.0155	8.88E-01
Cervix_Ectocervix	-0.0409	0.0233	9.61E-01	-0.0284	0.0195	9.28E-01	-0.0127	0.0161	7.85E-01
Fallopian_Tube	-0.0379	0.0216	9.61E-01	-0.0196	0.0181	8.61E-01	-0.0162	0.0150	8.60E-01
Adrenal_Gland	-0.0278	0.0154	9.65E-01	-0.0055	0.0133	6.59E-01	0.0009	0.0115	4.69E-01
Thyroid	-0.0321	0.0165	9.74E-01	-0.0215	0.0141	9.37E-01	-0.0062	0.0120	6.97E-01
Skin_Not_Sun_Exposed_Suprapubic	-0.0234	0.0119	9.76E-01	-0.0204	0.0104	9.75E-01	-0.0043	0.0092	6.82E-01
Prostate	-0.0430	0.0207	9.81E-01	-0.0389	0.0174	9.88E-01	-0.0142	0.0145	8.36E-01
Skin_Sun_Exposed_Lower_leg	-0.0248	0.0118	9.82E-01	-0.0218	0.0104	9.82E-01	-0.0068	0.0091	7.73E-01
Heart_Atrial_Appendage	-0.0334	0.0146	9.89E-01	-0.0200	0.0127	9.43E-01	-0.0209	0.0111	9.70E-01
Heart_Left_Ventricle	-0.0305	0.0131	9.90E-01	-0.0243	0.0115	9.83E-01	-0.0176	0.0102	9.58E-01

Bladder	-0.0488	0.0208	9.91E-01	-0.0338	0.0175	9.73E-01	-0.0209	0.0146	9.24E-01
Artery_Aorta	-0.0404	0.0167	9.92E-01	-0.0206	0.0143	9.25E-01	-0.0164	0.0123	9.09E-01
Colon_Transverse	-0.0457	0.0174	9.96E-01	-0.0351	0.0148	9.91E-01	-0.0168	0.0126	9.08E-01
Vagina	-0.0521	0.0194	9.96E-01	-0.0334	0.0164	9.79E-01	-0.0201	0.0138	9.28E-01
Kidney_Cortex	-0.0394	0.0146	9.97E-01	-0.0224	0.0126	9.62E-01	-0.0180	0.0110	9.50E-01
Minor_Salivary_Gland	-0.0423	0.0157	9.97E-01	-0.0325	0.0135	9.92E-01	-0.0183	0.0116	9.42E-01
Esophagus_Mucosa	-0.0309	0.0114	9.97E-01	-0.0212	0.0100	9.83E-01	-0.0061	0.0089	7.54E-01
Stomach	-0.0549	0.0200	9.97E-01	-0.0363	0.0170	9.84E-01	-0.0361	0.0144	9.94E-01
Spleen	-0.0307	0.0109	9.98E-01	-0.0260	0.0096	9.97E-01	0.0050	0.0084	2.78E-01
Pancreas	-0.0414	0.0129	9.99E-01	-0.0220	0.0114	9.73E-01	-0.0144	0.0101	9.23E-01
Small_Intestine_Terminal_Ileum	-0.0424	0.0130	9.99E-01	-0.0272	0.0113	9.92E-01	-0.0056	0.0098	7.17E-01
Liver	-0.0301	0.0091	1.00	-0.0121	0.0081	9.32E-01	-0.0039	0.0073	7.03E-01
Artery_Coronary	-0.0701	0.0204	1.00	-0.0358	0.0172	9.82E-01	-0.0237	0.0144	9.49E-01
Lung	-0.0574	0.0154	1.00	-0.0307	0.0132	9.90E-01	-0.0155	0.0113	9.15E-01
Adipose_Subcutaneous	-0.0676	0.0179	1.00	-0.0349	0.0152	9.89E-01	-0.0178	0.0129	9.16E-01
Adipose_Visceral_Omentum	-0.0743	0.0185	1.00	-0.0288	0.0156	9.67E-01	-0.0219	0.0133	9.50E-01
Breast_Mammary_Tissue	-0.0896	0.0225	1.00	-0.0488	0.0187	9.95E-01	-0.0242	0.0155	9.41E-01

MAGMA analysis conditioned on gene size, log gene size, gene density, log gene density, inverse minor allele count, and log inverse minor allele count.

Supplementary Table 13. Causal links of sleep duration (in minutes) with health outcomes using Mendelian Randomization.

				Inverse Variance Weighted			MR Egger			Weighted Median		
**Outcome**	**Study**	**PMID**	**SNPs**	**Beta/ log OR**	***SE***	***P* value**	**Beta/ log OR**	***SE***	***P* value**	**Beta/ log OR**	***SE***	***P* value**
Insomnia, log OR	UK Biobank	N/A	73	-0.004	0.000	**2.87E-24**	-0.006	0.001	**8.70E-06**	-0.004	0.000	**1.32E-26**
Schizophrenia, log OR	PGC	25056061	67	0.0088	0.003	**3.70E-03**	0.015	0.011	0.20	0.008	0.003	**3.95E-03**
Bipolar disorder, log OR	PGC	21926972	53	0.006	0.004	1.52E-01	0.0002	0.018	0.99	0.001	0.006	8.90E-01
PGC cross-disorder traits, log OR	PGC	23453885	48	0.003	0.002	2.11E-01	0.001	0.010	0.95	0.001	0.003	8.62E-01
Age at menarche, years	ReproGen	25231870	53	0.003	0.001	3.74E-02	0.0001	0.006	0.98	0.002	0.002	2.82E-01
Body mass index, kg/m2	GIANT	25673413	53	0.001	0.001	0.42	0.001	0.003	0.72	0.001	0.001	0.41
Type 2 diabetes, log OR	DIAGRAM	24509480	52	0.001	0.003	0.67	-0.009	0.010	0.40	0.000	0.004	1.00

Beta is the estimated effect of change on outcome in units indicated (continuous) or log odds ratio for case/control traits using the SNPs associated with sleep duration with available proxies in each study (number of SNPs used indicated in the SNPs column) using effect estimates for sleep duration in minutes (Supplementary Data 1). Three Mendelian Randomization methods were used. Each outcome was obtained from publicly available GWAS summary data. The number of SNPs used varies for each outcome because of not being able to find some of the 78 SNPs (or proxies) in the summary data.

Supplementary Table 14. Bidirectional causal links of sleep duration (in minutes) with schizophrenia (log odds ratio) using two-sample Mendelian Randomization.

			Inverse Variance Weighted			MR Egger			Weighted Median		
**Exposure**	**Outcome**	**SNPs**	**Beta**	***SE***	***P* value**	**Beta**	***SE***	***P* value**	**Beta**	***SE***	***P* value**
Sleep duration (UK Biobank - baseline adjustment)	Schizophrenia (PGC)	67	0.009	0.003	**3.70E-03**	0.015	0.011	0.20	0.008	0.003	**3.95E-03**
Sleep duration (UK Biobank - BMI adjustment)	Schizophrenia (PGC)	65	0.009	0.003	**5.18E-03**	0.016	0.012	0.18	0.008	0.003	**5.12E-03**
Sleep duration (UK Biobank - insomnia adjustment)	Schizophrenia (PGC)	43	0.009	0.003	**8.48E-03**	0.012	0.012	0.34	0.009	0.003	**1.05E-02**
Sleep duration (UK Biobank - lifestyle adjustment)	Schizophrenia (PGC)	48	0.008	0.003	**2.03E-02**	0.011	0.012	0.37	0.008	0.003	**1.46E-02**
Sleep duration (UK Biobank - only meta-analysis GWAS loci)	Schizophrenia (PGC)	48	0.006	0.003	6.16E-02	0.0157	0.012	0.20	0.008	0.003	**8.14E-03**
**Exposure**	**Outcome**	**SNPs**	**Log OR**	***SE***	***P* value**	**Log OR**	***SE***	***P* value**	**Log OR**	***SE***	***P* value**
Schizophrenia (PGC)	Sleep duration (UK Biobank - baseline adjustment)	69	0.025	0.007	**6.05E-04**	0.026	0.027	0.35	0.026	0.006	**3.36E-05**

Abbreviations: MR =Mendlian randomization; OR =odds ratio
