# Supplementary material for: Genome-wide association study identifies genetic loci for self-reported habitual sleep duration supported by accelerometer-derived estimates
Source: Nat Commun. 2019 Mar 7;10:1100. doi: 10.1038/s41467-019-08917-4 (PMC6405943; doi:10.1038/s41467-019-08917-4)
Supplement: Supplementary file 3 — Description of Additional Supplementary Files [file 41467_2019_8917_MOESM3_ESM.pdf]

Description of Additional Supplementary Files

-
- **Supplementary Data 1:** Genome-wide association signals (P <5x10-8) for sleep duration in participants of European ancestry from UK Biobank (*n* =446,118) and corresponding results from sleep duration GWAS with additional exclusions for shift work and prevalent chronic diseases and psychiatric disorders (*n* =326,224).
- **Supplementary Data 2:** Regional association plots for genome-wide significant sleep duration loci (P <$5x10^{-8}$), short/long sleep loci, and loci from meta-analyses with CHARGE (adults), EAGLE (children), or both (CHARGE + EAGLE). The panels show $-\log_{10} P$ values for lead SNPs. The SNPs shown are those within 400 kb of the lead SNP. LD is indicated in colorscale in relation to the highlighted marker (purple). The scheme is red for strong LD ($r^2 \geq 0.8$), orange, green and blue for lower LD, and navy blue for no LD. chr, chromosome; cM, centimorgan; LD, linkage disequilibrium; Mb, mega base pair; SNP, single-nucleotide polymorphism.
- **Supplementary Data 3:** Sensitivity analysis for genome-wide significant sleep duration loci (P <$5x10^{-8}$) in participants of European ancestry from UK Biobank (*n* =446,118), adjusting for additional known risk factors.
- **Supplementary Data 4:** Genome-wide association signals (P <$5x10^{-8}$) for short sleep in participants of European ancestry from UK Biobank (*n* =106,192 cases/305,742 controls) and corresponding results from long sleep (*n* =34,184 cases/305,742 controls) and sleep duration (n =446,118) GWAS.
- **Supplementary Data 5:** Sensitivity analysis for genome-wide significant short sleep loci (P <$5x10^{-8}$) in participants of European ancestry from UK Biobank (*n* =106,192 cases/305,742 controls), adjusting for known risk factors.
- **Supplementary Data 6:** Replication of UK Biobank 78 sleep duration signals in CHARGE (adult) and EAGLE (childhood/adolescent) studies and meta-analyses with UK Biobank.
- **Supplementary Data 7:** Additional new loci (P <$5x10^{-8}$) unraveled for sleep duration UK Biobank sleep duration genome-wide association meta-analyses with CHARGE (adult; *n* =493,298), EAGLE (childhood/adolescent; *n* =456,672) or both (CHARGE + EAGLE; *n* =503,852).
- **Supplementary Data 8:** Genetic variants for self-reported sleep duration association with accelerometer-derived estimates of sleep duration, fragmentation, and timing in the UK Biobank (*n* =85,499).
- **Supplementary Data 9:** Genetic variants for self-reported short and long sleep associations with accelerometer-derived estimates of sleep duration, fragmentation, and timing in the UK Biobank (*n* =85,499).
- **Supplementary Data 10:** Annotation of genes under association signals for sleep duration.
- **Supplementary Data 11:** Functional annotation of sleep duration SNPs with a causal probability larger than 0.2.
- **Supplementary Data 12:** Functional annotation of short and long sleep SNPs with a causal probability larger than 0.2.
- **Supplementary Data 13:** Partitioned heritability for sleep duration traits across functional annotation classes using LDSC. We tested 50 functional annotations; annotations passing the multiple-testing significance threshold (P <$2.0x10^{-3}$) are bolded.
- **Supplementary Data 14:** Gene-based association analysis using Pascal (summing statistics across independent signals in each gene) for sleep duration.
- **Supplementary Data 15:** Gene-based association analysis using Pascal (summing statistics across independent signals in each gene) for short and long sleep.
- **Supplementary Data 16:** Single cell enrichment analysis utilizing the Tabula Muris dataset. Bold denotes significant enrichment based on Bonferroni correction accounting for all 115 tested cell types (P <$4.3x10^{-4}$).
- **Supplementary Data 17:** Significant results from transcriptome-wide association analyses (TWAS) on enriched tissue types for all traits. Significant results are based on Bonferroni correction for the number of genes tested per tissue and for all 11 tissues.
- **Supplementary Data 18:** Genetic correlation between sleep duration and 45 traits using LD-score regression. Significant correlations with P <$2.2x10^{-4}$ accounting for 224 tested traits are bolded.

- **Supplementary Data 19:** Leave-one-out Mendelian Randomization sensitivity analysis for causal links of sleep duration (in minutes) on schizophrenia (log odds ratio) using inverse variance weighted method.
