# Supplementary material for: Genome-wide association study identifies genetic loci for self-reported habitual sleep duration supported by accelerometer-derived estimates
Source: Nat Commun. 2019 Mar 7;10:1100. doi: 10.1038/s41467-019-08917-4 (PMC6405943; doi:10.1038/s41467-019-08917-4)
Supplement: Supplementary file 5 — Supplementary Data 2 [file 41467_2019_8917_MOESM5_ESM.pdf]

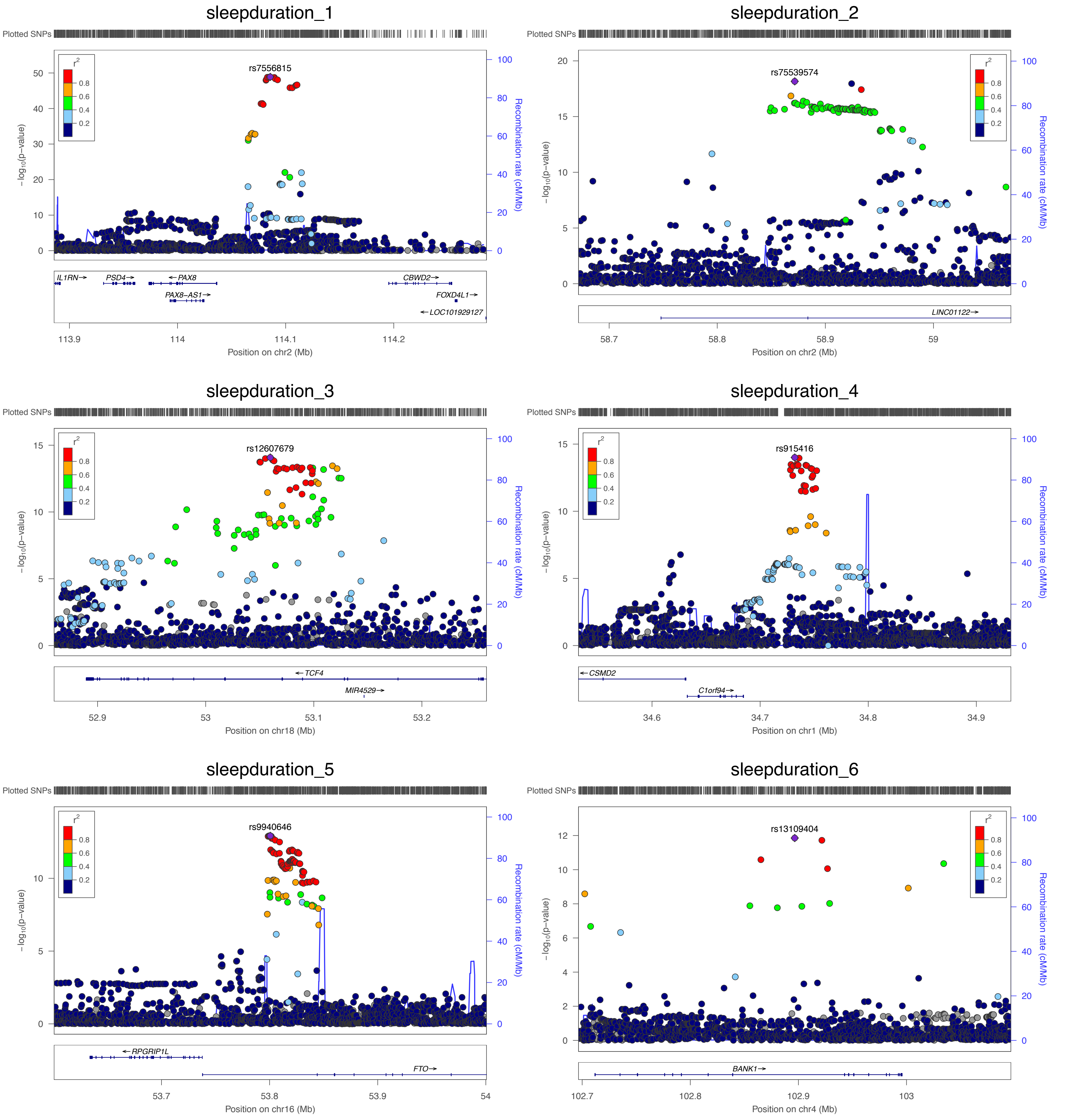
sleepduration_1
Plotted SNPs
rs7556815
r2
0.8
0.6
0.4
0.2
−log10(p−value)
Recombination rate (cM/Mb)
IL1RN
PSD4
PAX8
PAX8−AS1
CBWD2
FOXD4L1
LOC101929127
Position on chr2 (Mb)
sleepduration_2
Plotted SNPs
rs75539574
LINC01122
Position on chr2 (Mb)
sleepduration_3
Plotted SNPs
rs12607679
TCF4
MIR4529
Position on chr18 (Mb)
sleepduration_4
Plotted SNPs
rs915416
CSMD2
C1orf94
Position on chr1 (Mb)
sleepduration_5
Plotted SNPs
rs9940646
RPGRIP1L
FTO
Position on chr16 (Mb)
sleepduration_6
Plotted SNPs
rs13109404
BANK1
Position on chr4 (Mb)

sleepduration_7

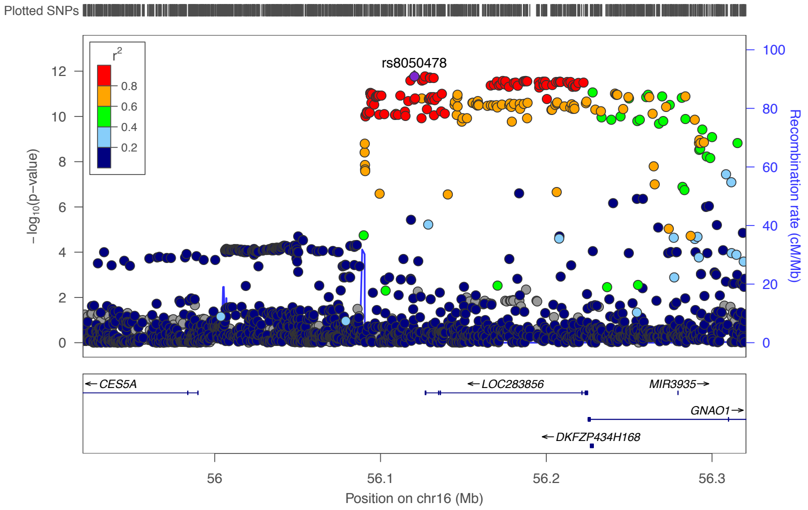

sleepduration_8

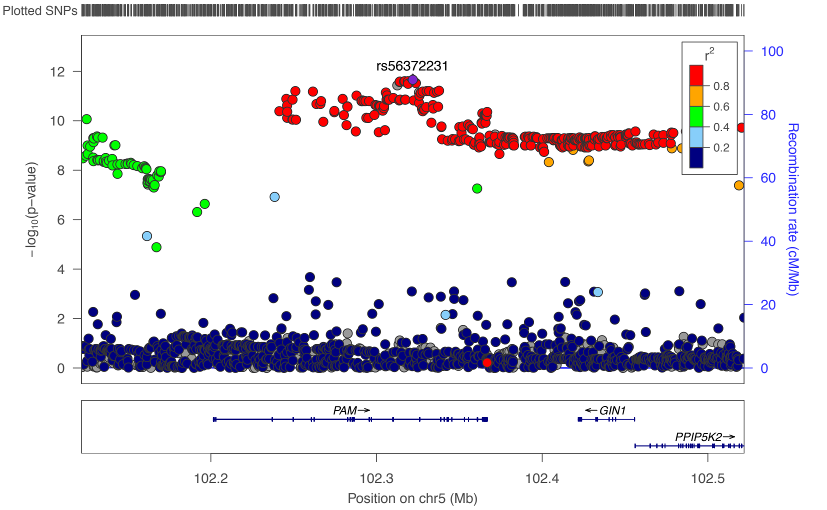

sleepduration_9

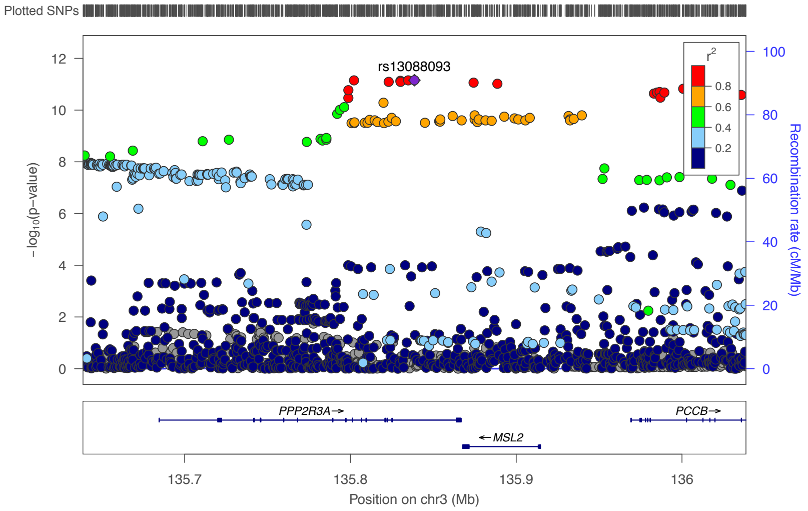

sleepduration_10

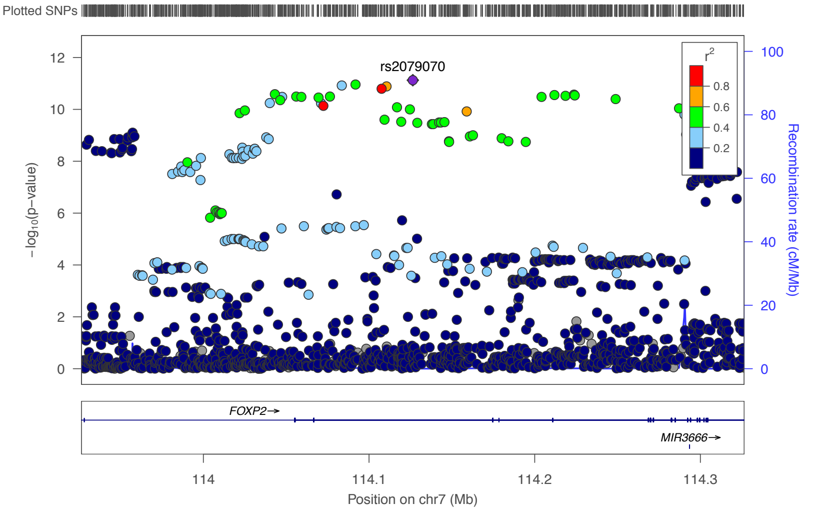

sleepduration_11

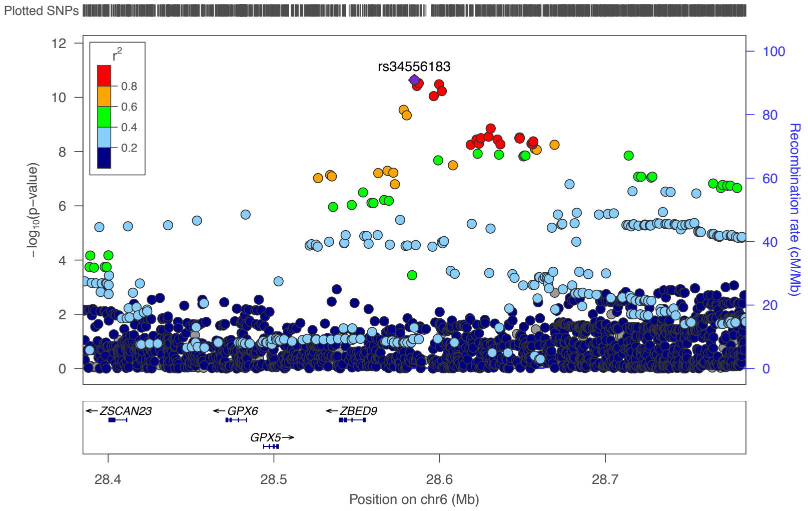

sleepduration_12

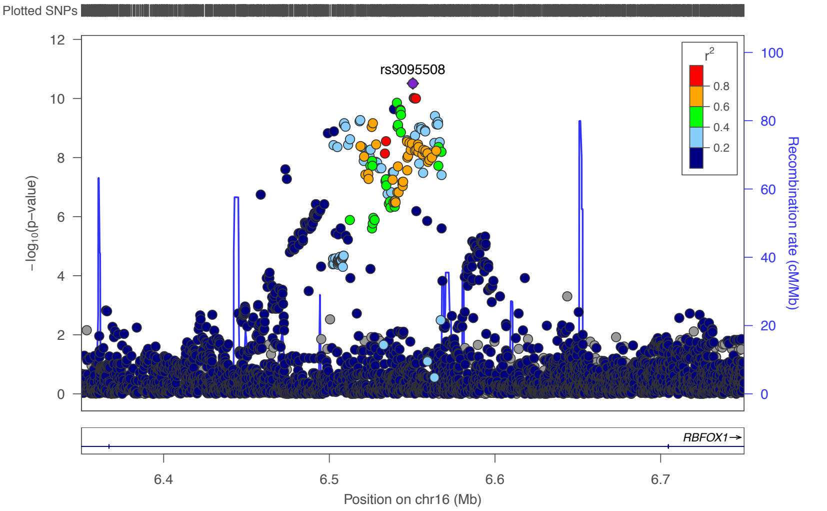

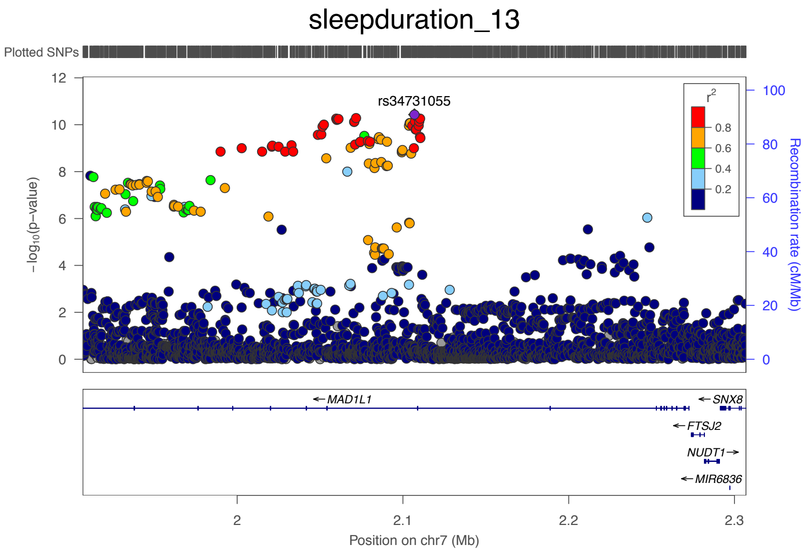
sleepduration_13
Plotted SNPs
rs34731055
−log10(p−value)
Recombination rate (cM/Mb)
r2
MAD1L1
SNX8
FTSJ2
NUDT1
MIR6836
Position on chr7 (Mb)

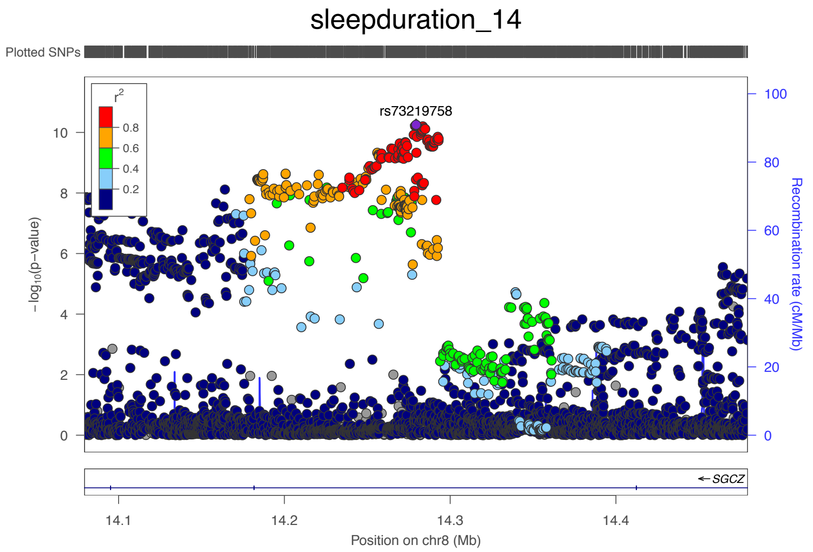
sleepduration_14
Plotted SNPs
rs73219758
−log10(p−value)
Recombination rate (cM/Mb)
r2
SGCZ
Position on chr8 (Mb)

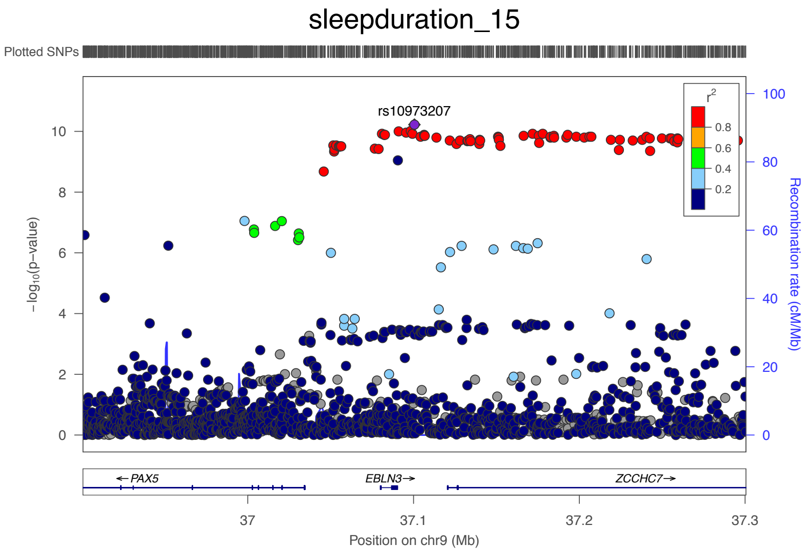
sleepduration_15
Plotted SNPs
rs10973207
−log10(p−value)
Recombination rate (cM/Mb)
r2
PAX5
EBLN3
ZCCHC7
Position on chr9 (Mb)

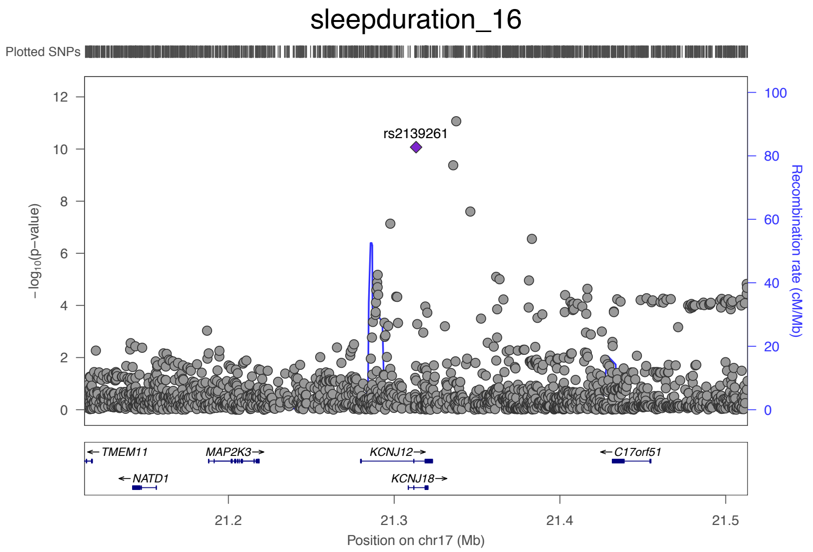
sleepduration_16
Plotted SNPs
rs2139261
−log10(p−value)
Recombination rate (cM/Mb)
TMEM11
MAP2K3
KCNJ12
C17orf51
NATD1
KCNJ18
Position on chr17 (Mb)

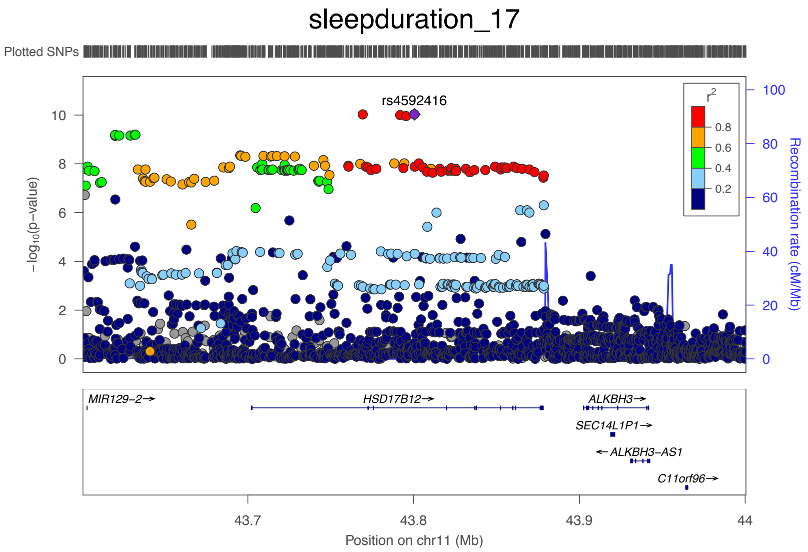
sleepduration_17
Plotted SNPs
rs4592416
−log10(p−value)
Recombination rate (cM/Mb)
r2
MIR129−2
HSD17B12
ALKBH3
SEC14L1P1
ALKBH3−AS1
C11orf96
Position on chr11 (Mb)

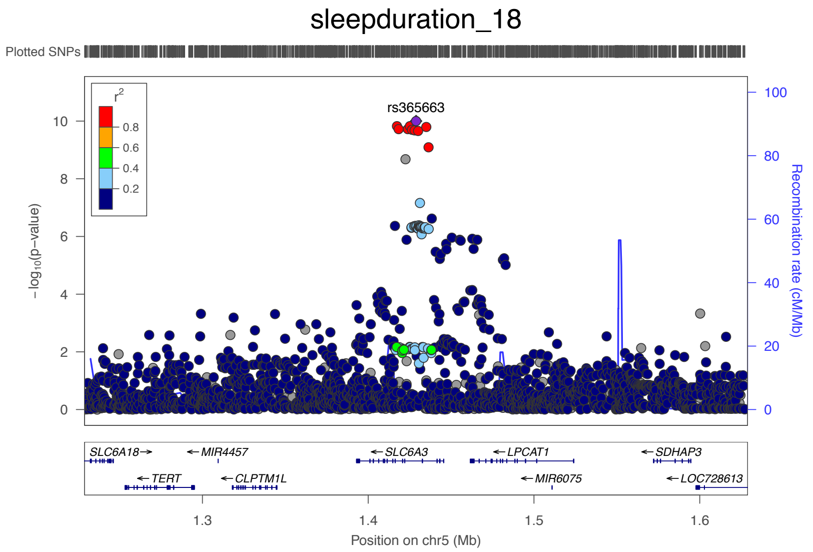
sleepduration_18
Plotted SNPs
rs365663
−log10(p−value)
Recombination rate (cM/Mb)
r2
SLC6A18
MIR4457
SLC6A3
LPCAT1
SDHAP3
TERT
CLPTM1L
MIR6075
LOC728613
Position on chr5 (Mb)

sleepduration_19

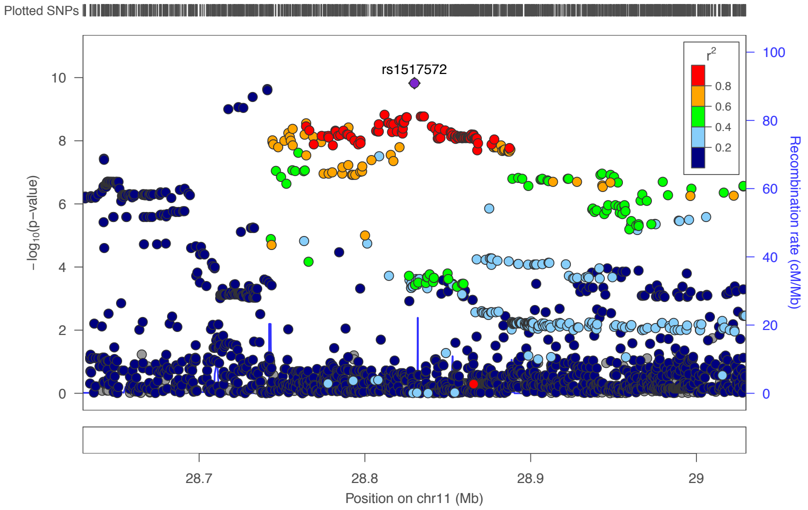

sleepduration_20

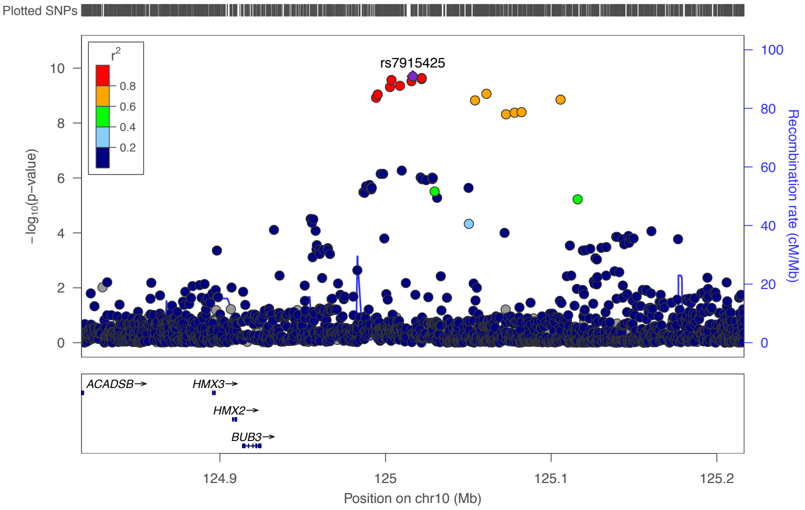

sleepduration_21

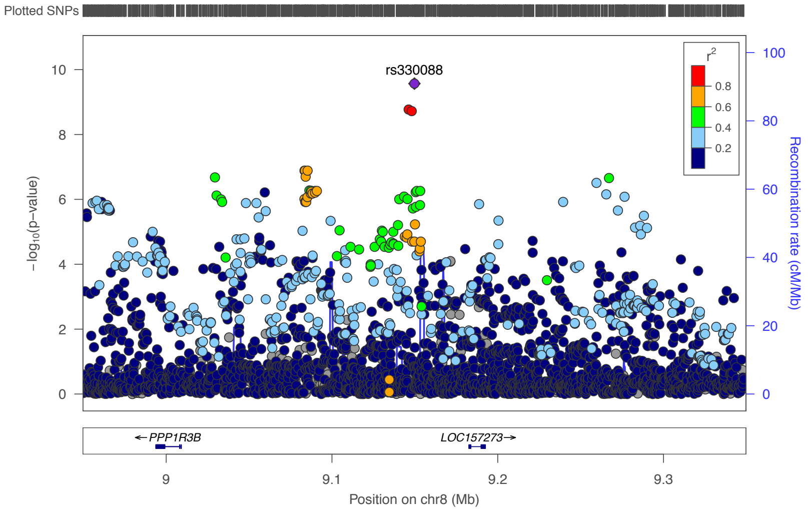

sleepduration_22

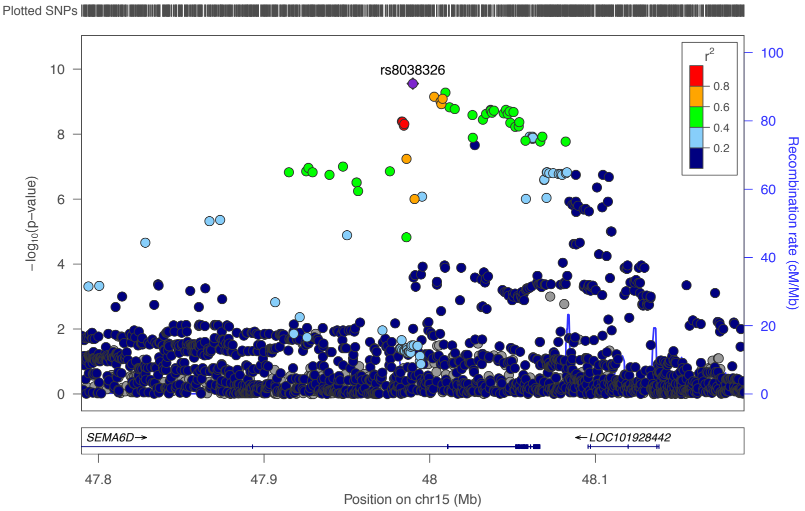

sleepduration_23

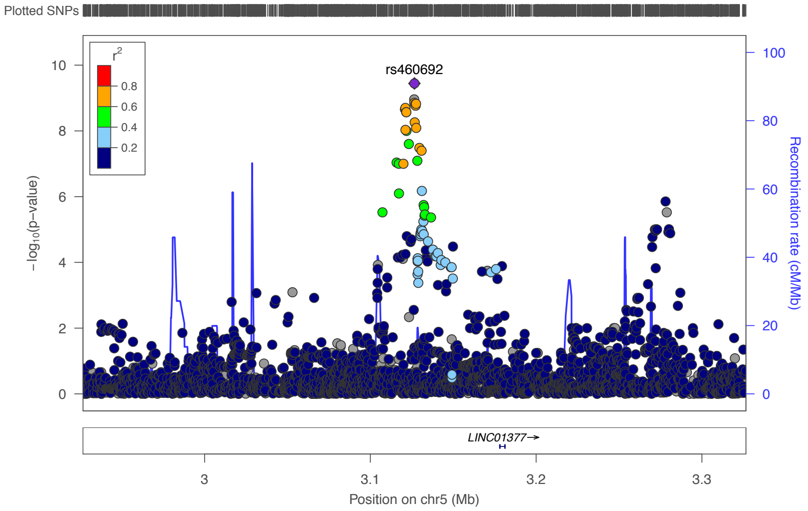

sleepduration_24

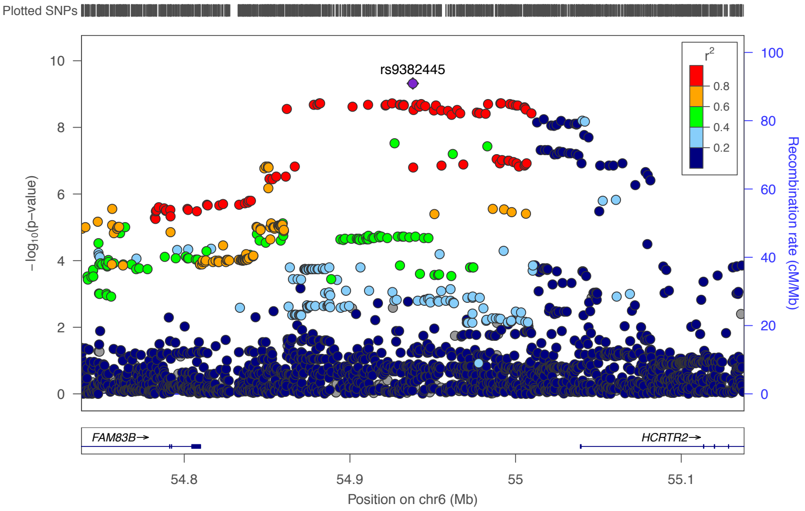

sleepduration_25

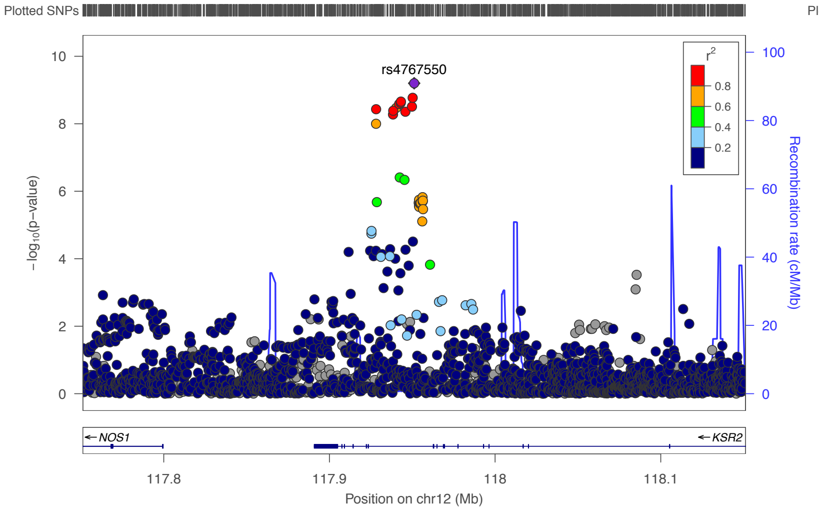

sleepduration_26

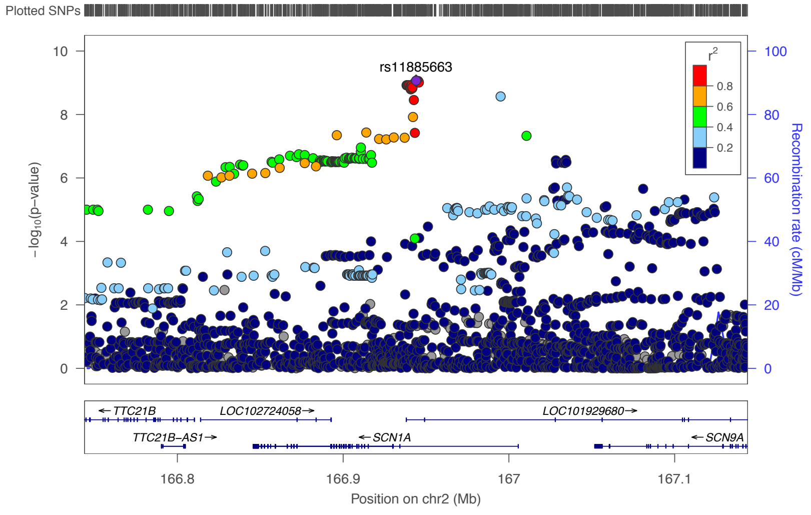

sleepduration_27

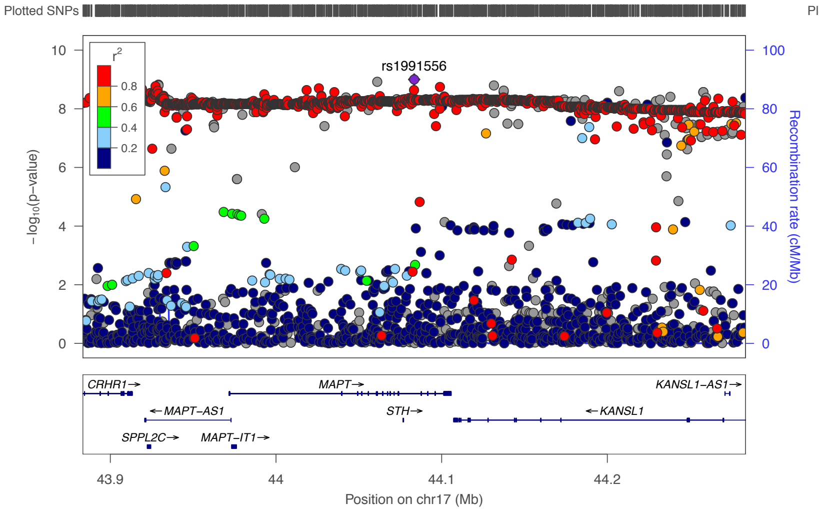

sleepduration_28

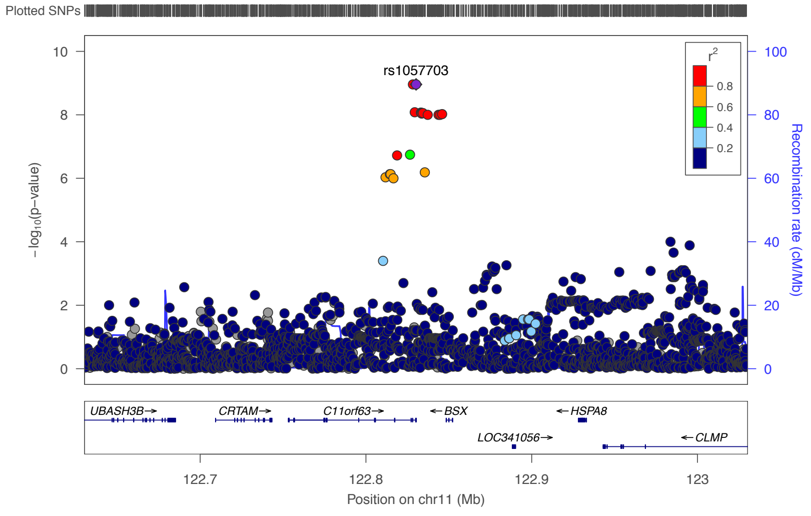

sleepduration_29

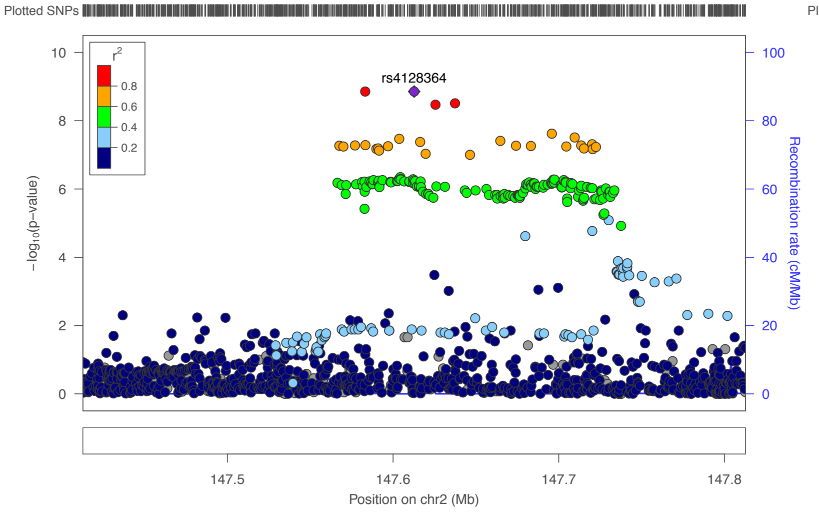

sleepduration_30

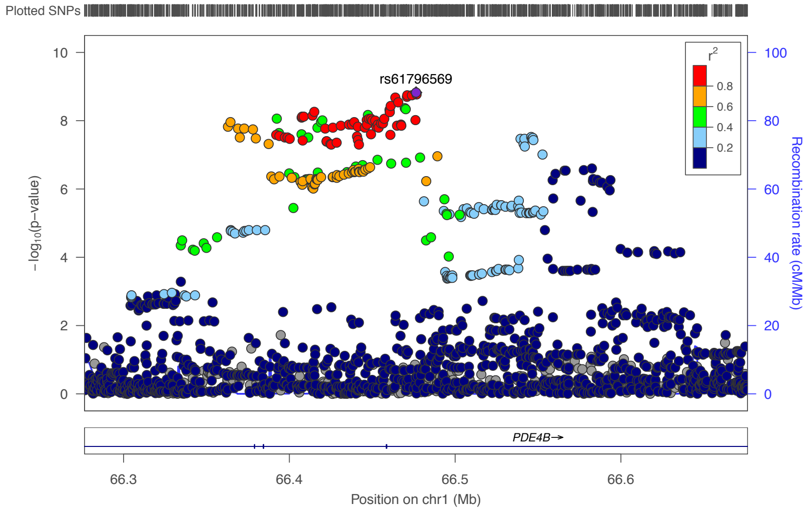

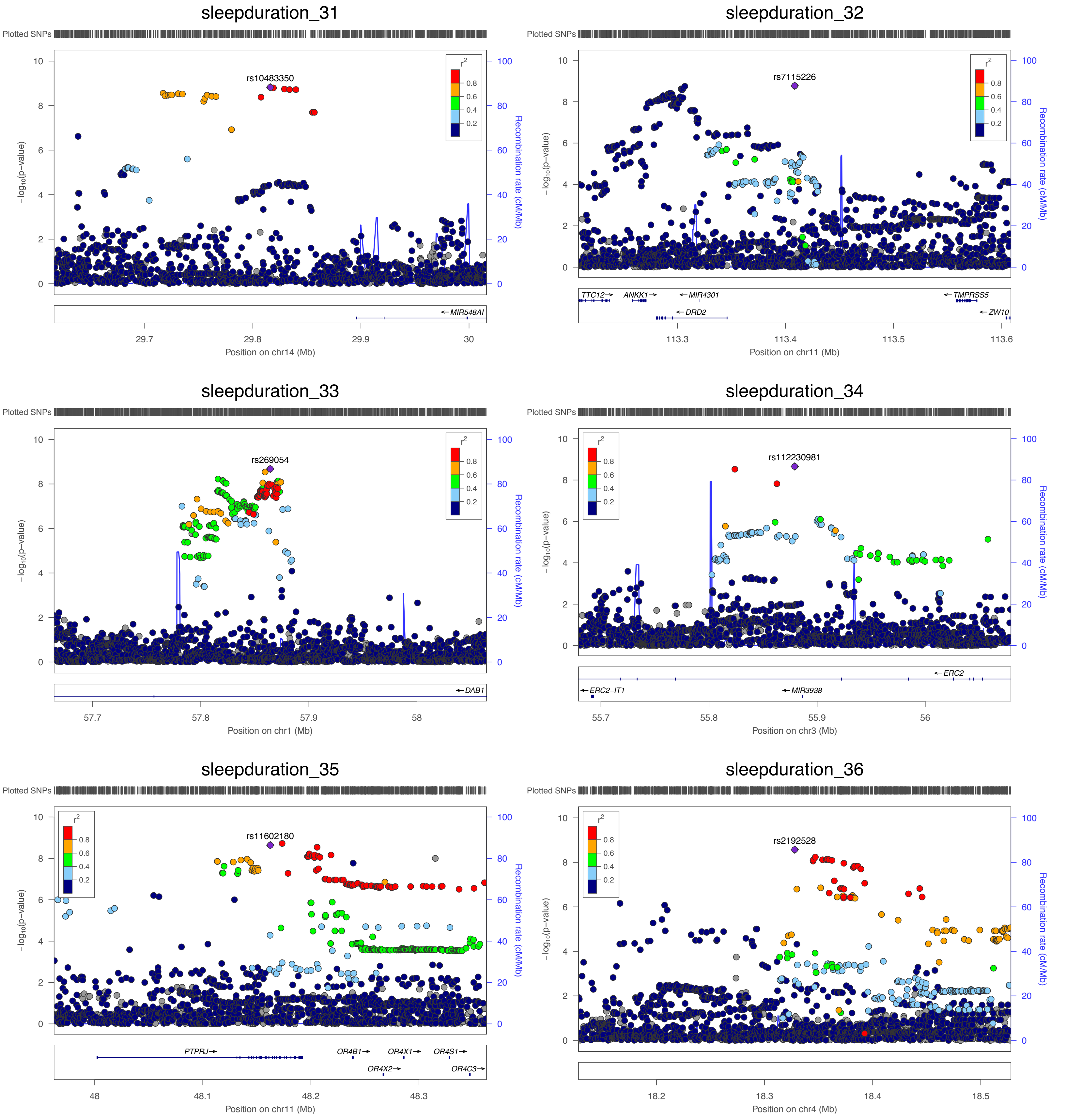
sleepduration_31
Plotted SNPs
rs10483350
r2
0.8
0.6
0.4
0.2
−log10(p−value)
Recombination rate (cM/Mb)
MIR548AI
29.7
29.8
29.9
30
Position on chr14 (Mb)
sleepduration_32
rs7115226
TTC12
ANKK1
MIR4301
DRD2
TMPRSS5
ZW10
113.3
113.4
113.5
113.6
Position on chr11 (Mb)
sleepduration_33
rs269054
DAB1
57.7
57.8
57.9
58
Position on chr1 (Mb)
sleepduration_34
rs112230981
ERC2
ERC2−IT1
MIR3938
55.7
55.8
55.9
56
Position on chr3 (Mb)
sleepduration_35
rs11602180
PTPRJ
OR4B1
OR4X1
OR4S1
OR4X2
OR4C3
48
48.1
48.2
48.3
Position on chr11 (Mb)
sleepduration_36
rs2192528
18.2
18.3
18.4
18.5
Position on chr4 (Mb)

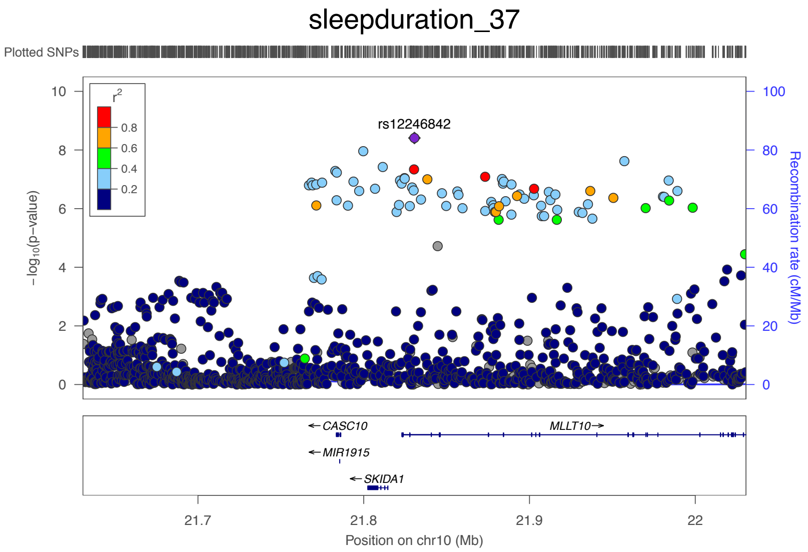
sleepduration_37
Plotted SNPs
rs12246842
r2
0.8
0.6
0.4
0.2
−log10(p−value)
Recombination rate (cM/Mb)
CASC10
MLLT10
MIR1915
SKIDA1
21.7
21.8
21.9
22
Position on chr10 (Mb)

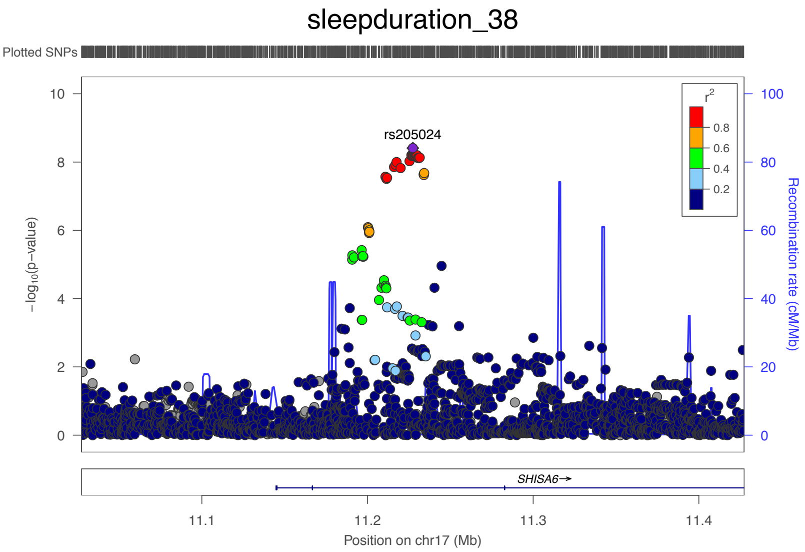
sleepduration_38
Plotted SNPs
rs205024
r2
0.8
0.6
0.4
0.2
−log10(p−value)
Recombination rate (cM/Mb)
SHISA6
11.1
11.2
11.3
11.4
Position on chr17 (Mb)

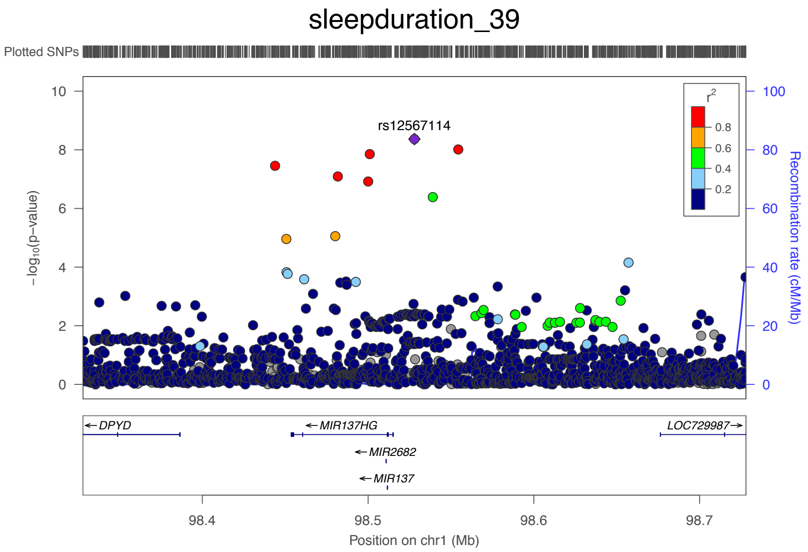
sleepduration_39
Plotted SNPs
rs12567114
r2
0.8
0.6
0.4
0.2
−log10(p−value)
Recombination rate (cM/Mb)
DPYD
MIR137HG
LOC729987
MIR2682
MIR137
98.4
98.5
98.6
98.7
Position on chr1 (Mb)

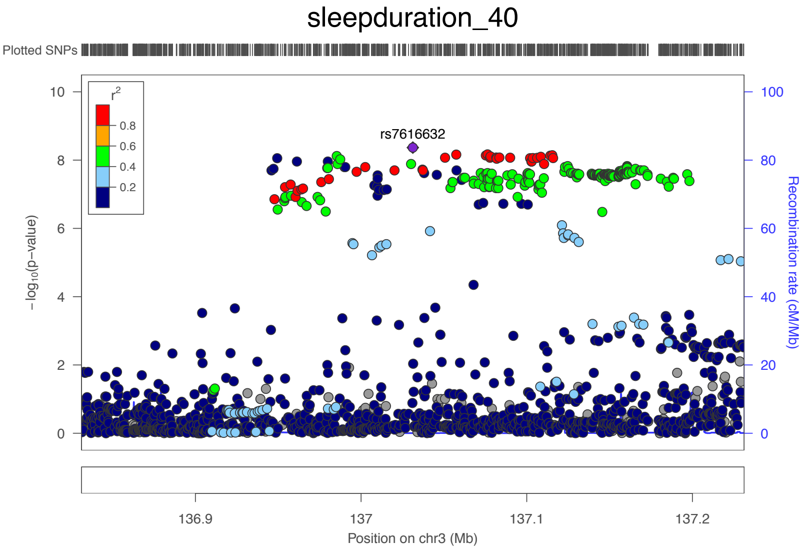
sleepduration_40
Plotted SNPs
rs7616632
r2
0.8
0.6
0.4
0.2
−log10(p−value)
Recombination rate (cM/Mb)
136.9
137
137.1
137.2
Position on chr3 (Mb)

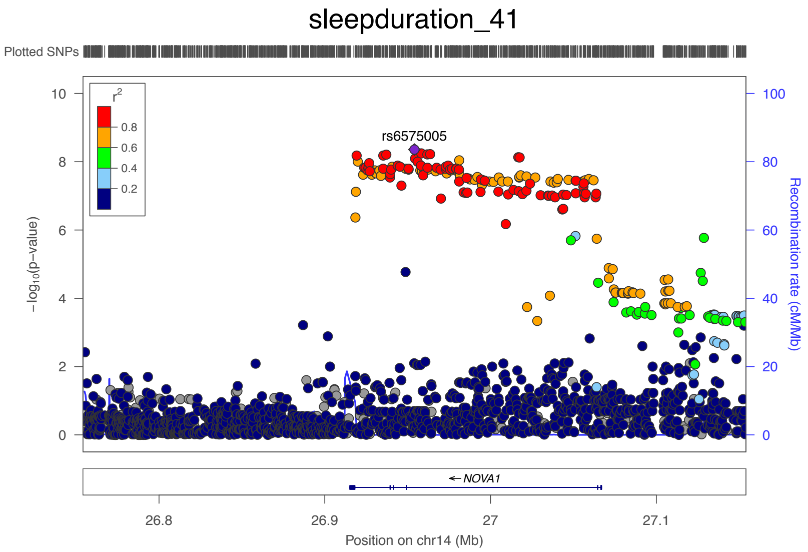
sleepduration_41
Plotted SNPs
rs6575005
r2
0.8
0.6
0.4
0.2
−log10(p−value)
Recombination rate (cM/Mb)
NOVA1
26.8
26.9
27
27.1
Position on chr14 (Mb)

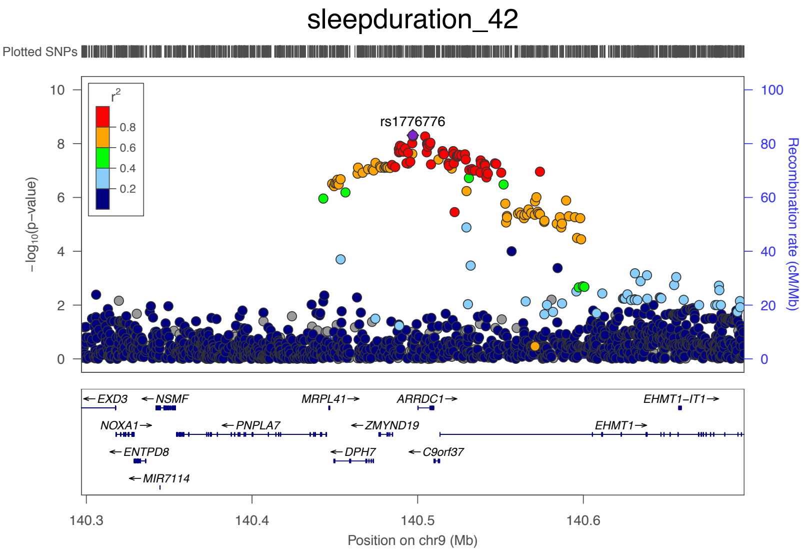
sleepduration_42
Plotted SNPs
rs1776776
r2
0.8
0.6
0.4
0.2
−log10(p−value)
Recombination rate (cM/Mb)
EXD3
NSMF
MRPL41
ARRDC1
EHMT1−IT1
NOXA1
PNPLA7
ZMYND19
EHMT1
ENTPD8
DPH7
C9orf37
MIR7114
140.3
140.4
140.5
140.6
Position on chr9 (Mb)

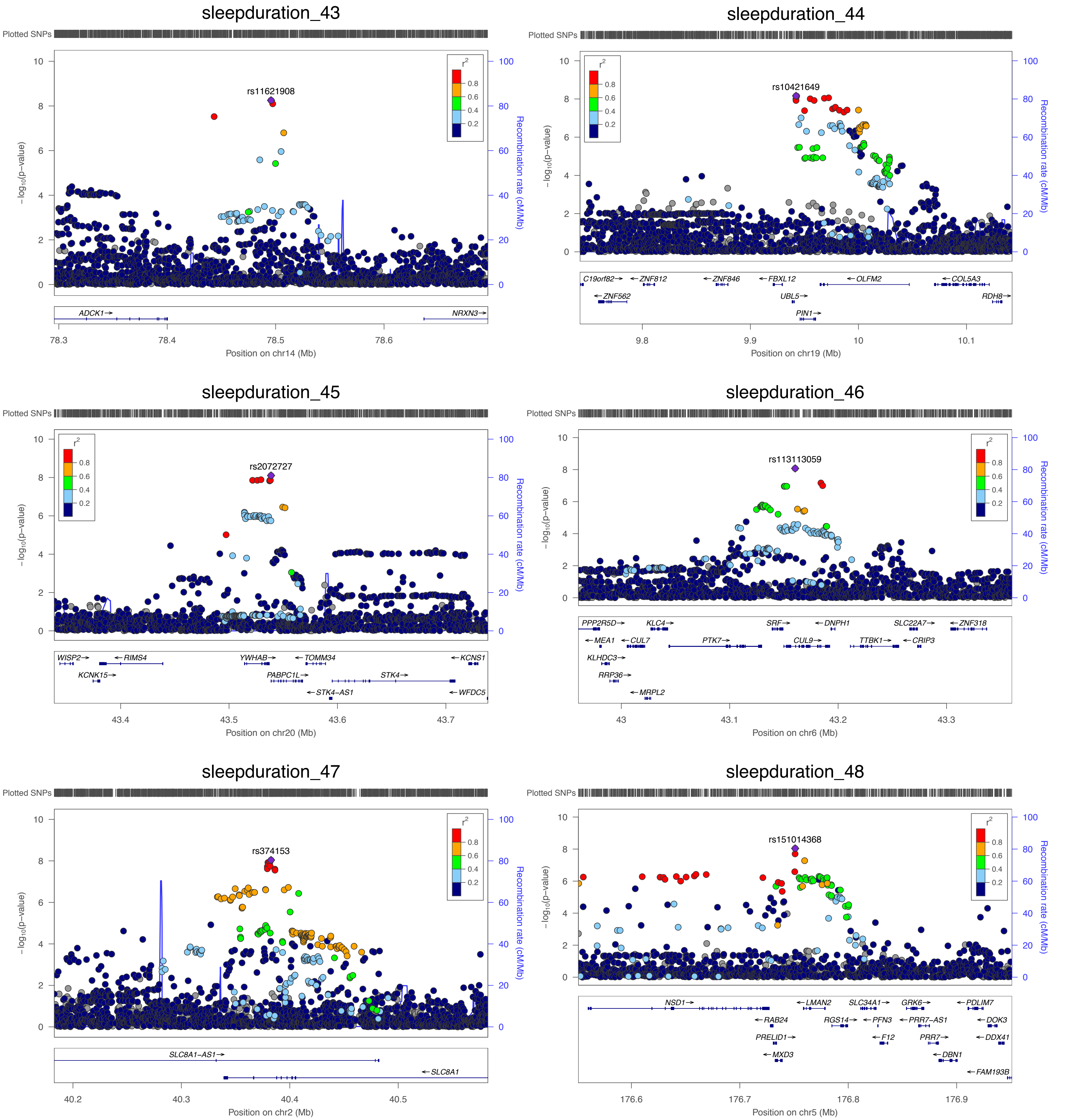
sleepduration_43
Plotted SNPs
rs11621908
ADCK1→
NRXN3→
Position on chr14 (Mb)
sleepduration_44
rs10421649
C19orf82→
←ZNF812
←ZNF846
←FBXL12
←OLFM2
←COL5A3
←ZNF562
UBL5→
RDH8→
PIN1→
Position on chr19 (Mb)
sleepduration_45
rs2072727
WISP2→
←RIMS4
YWHAB→
←TOMM34
←KCNS1
KCNK15→
PABPC1L→
STK4→
←STK4−AS1
←WFDC5
Position on chr20 (Mb)
sleepduration_46
rs113113059
PPP2R5D→
KLC4→
SRF→
←DNPH1
SLC22A7→
←ZNF318
←MEA1
←CUL7
PTK7→
CUL9→
TTBK1→
←CRIP3
KLHDC3→
RRP36→
←MRPL2
Position on chr6 (Mb)
sleepduration_47
rs374153
SLC8A1−AS1→
←SLC8A1
Position on chr2 (Mb)
sleepduration_48
rs151014368
NSD1→
←LMAN2
SLC34A1→
GRK6→
←PDLIM7
←RAB24
RGS14→
←PFN3
←PRR7−AS1
←DOK3
PRELID1→
←F12
PRR7→
←DDX41
←MXD3
←DBN1
←FAM193B
Position on chr5 (Mb)
−log10(p−value)
Recombination rate (cM/Mb)
r2

sleepduration_49

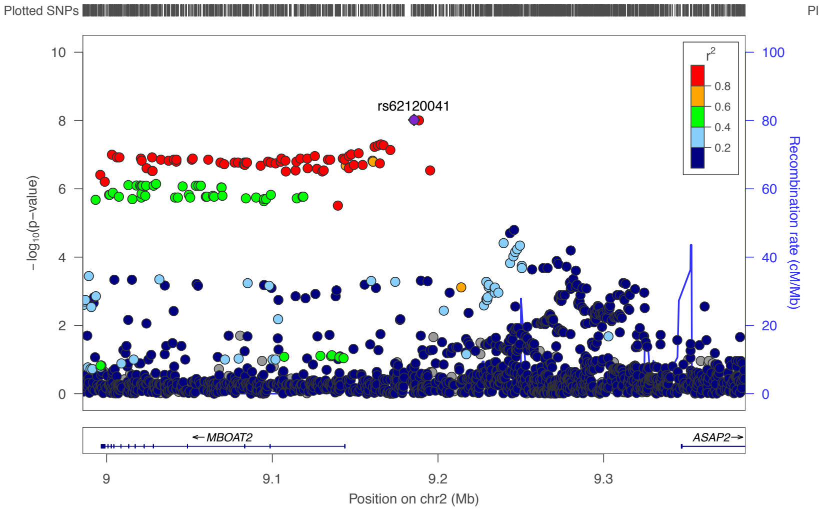

sleepduration_50

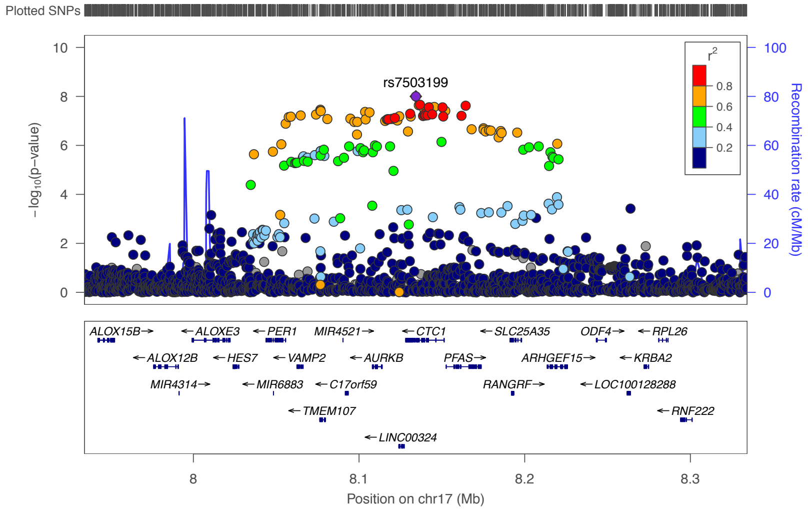

sleepduration_51

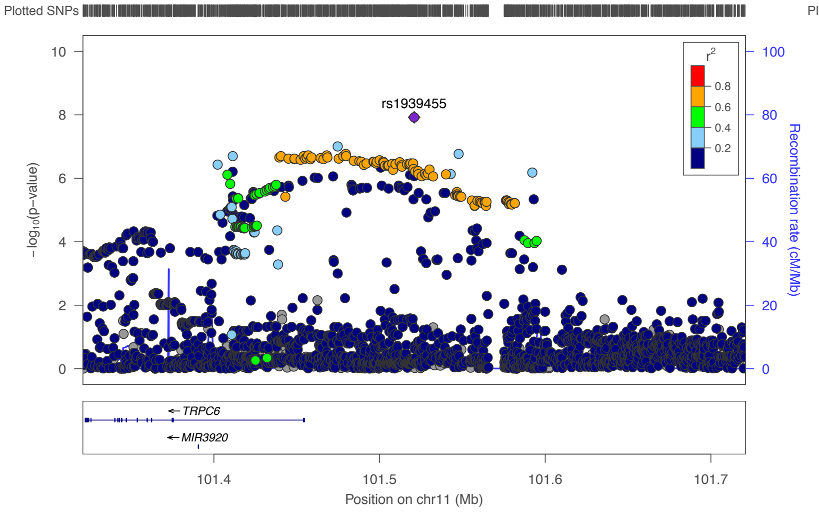

sleepduration_52

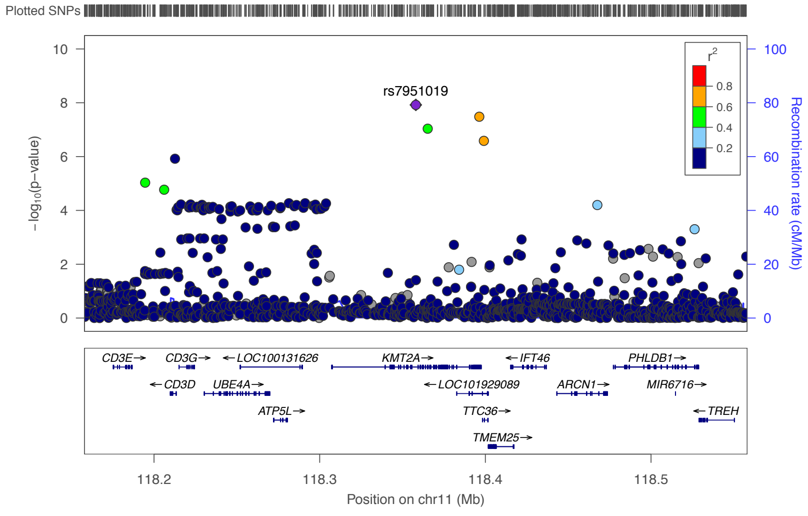

sleepduration_53

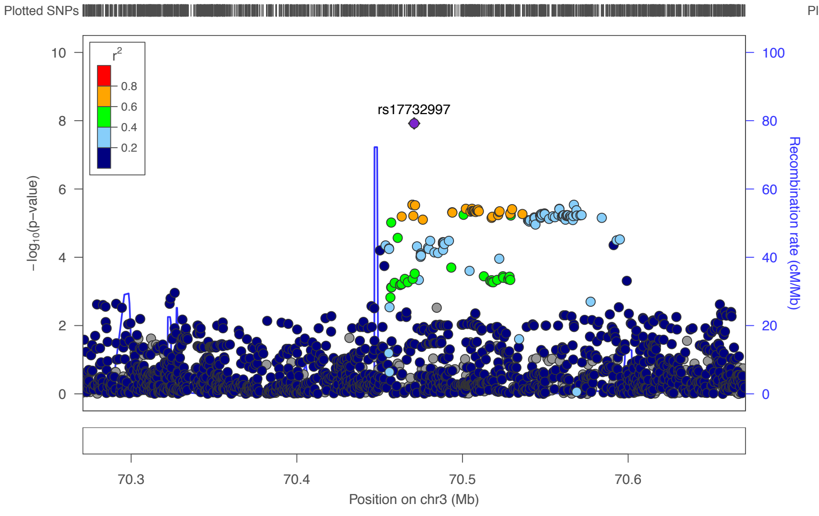

sleepduration_54

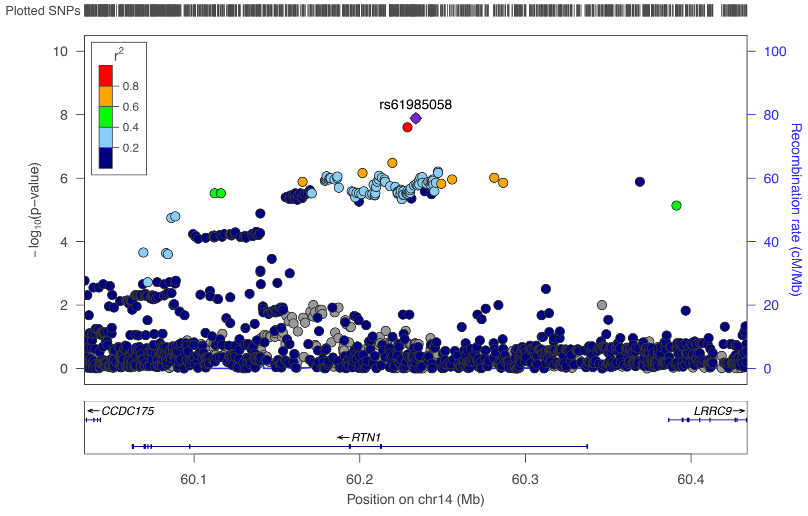

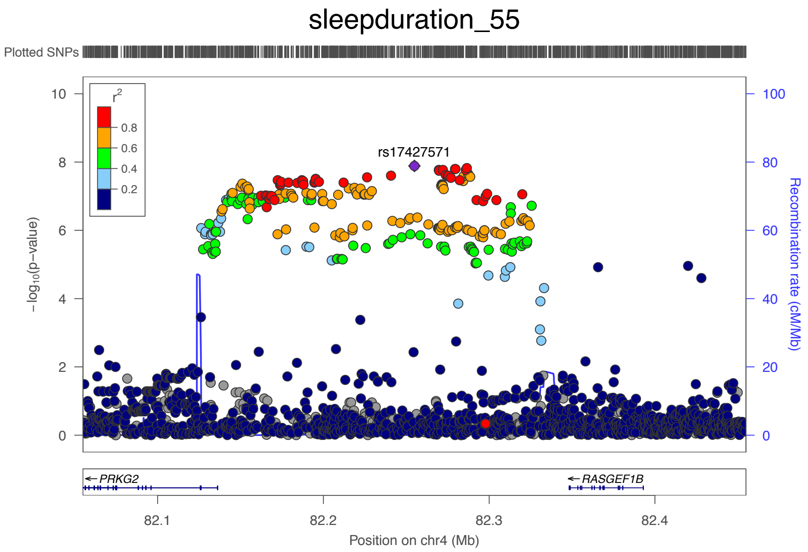
sleepduration_55
Plotted SNPs
rs17427571
PRKG2
RASGEF1B
−log10(p−value)
Recombination rate (cM/Mb)
Position on chr4 (Mb)
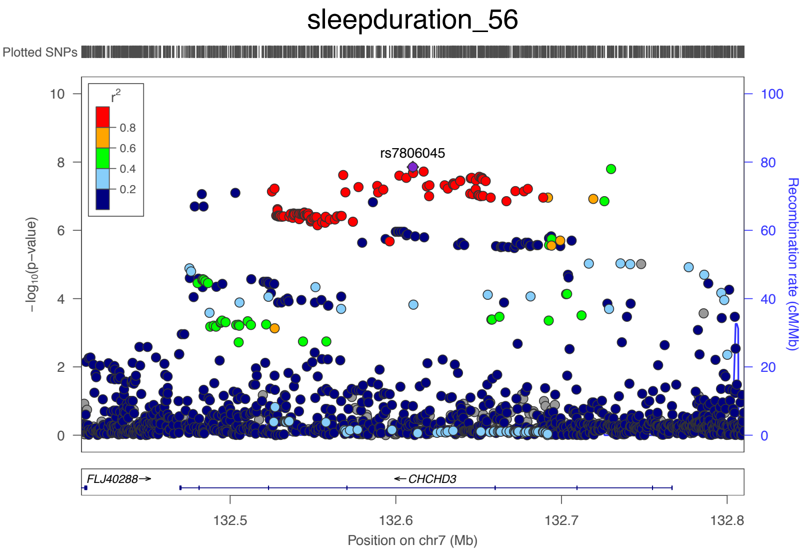
sleepduration_56
Plotted SNPs
rs7806045
FLJ40288
CHCHD3
−log10(p−value)
Recombination rate (cM/Mb)
Position on chr7 (Mb)
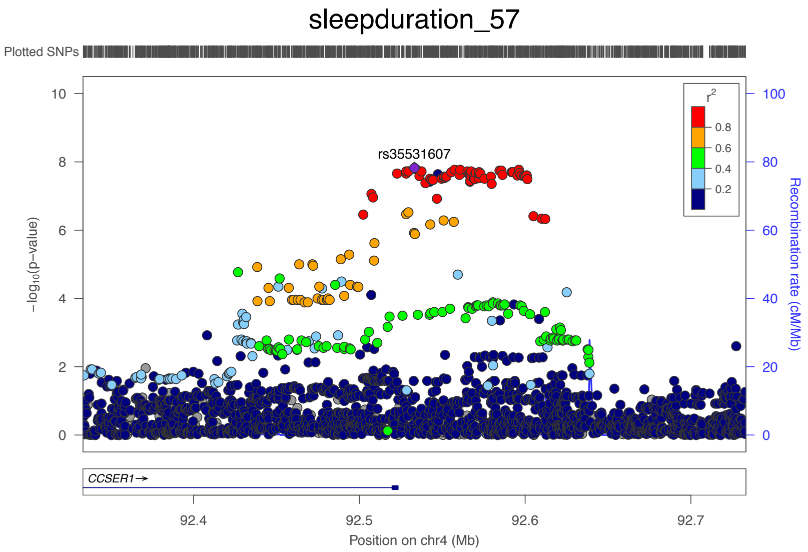
sleepduration_57
Plotted SNPs
rs35531607
CCSER1
−log10(p−value)
Recombination rate (cM/Mb)
Position on chr4 (Mb)
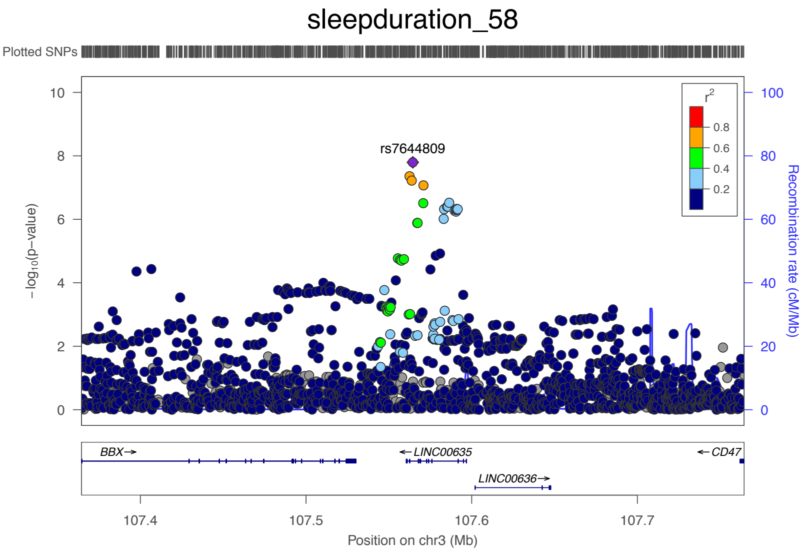
sleepduration_58
Plotted SNPs
rs7644809
BBX
LINC00635
CD47
LINC00636
−log10(p−value)
Recombination rate (cM/Mb)
Position on chr3 (Mb)
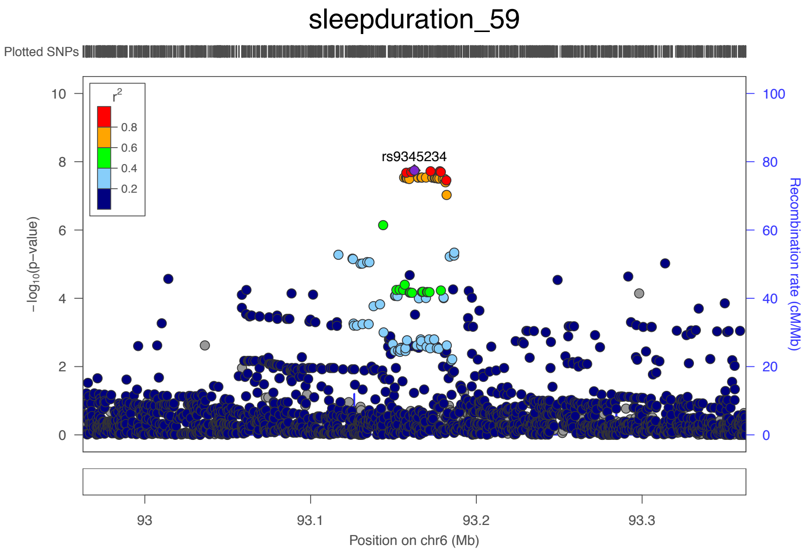
sleepduration_59
Plotted SNPs
rs9345234
−log10(p−value)
Recombination rate (cM/Mb)
Position on chr6 (Mb)
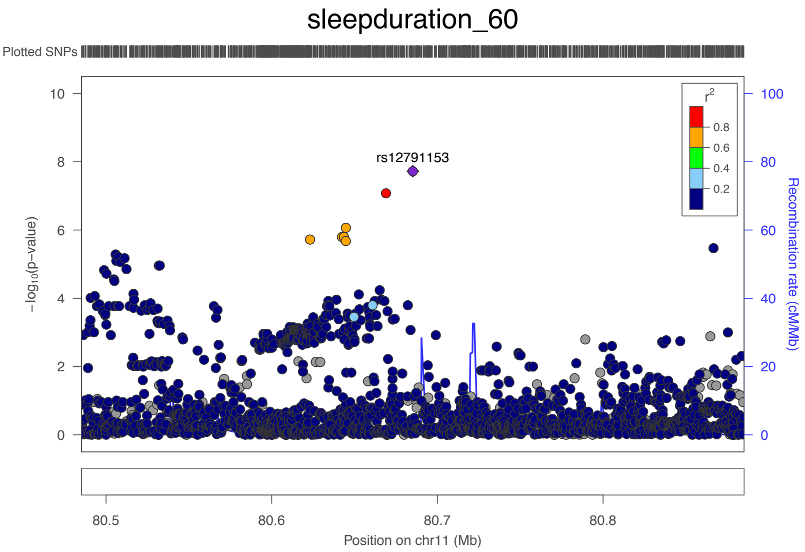
sleepduration_60
Plotted SNPs
rs12791153
−log10(p−value)
Recombination rate (cM/Mb)
Position on chr11 (Mb)

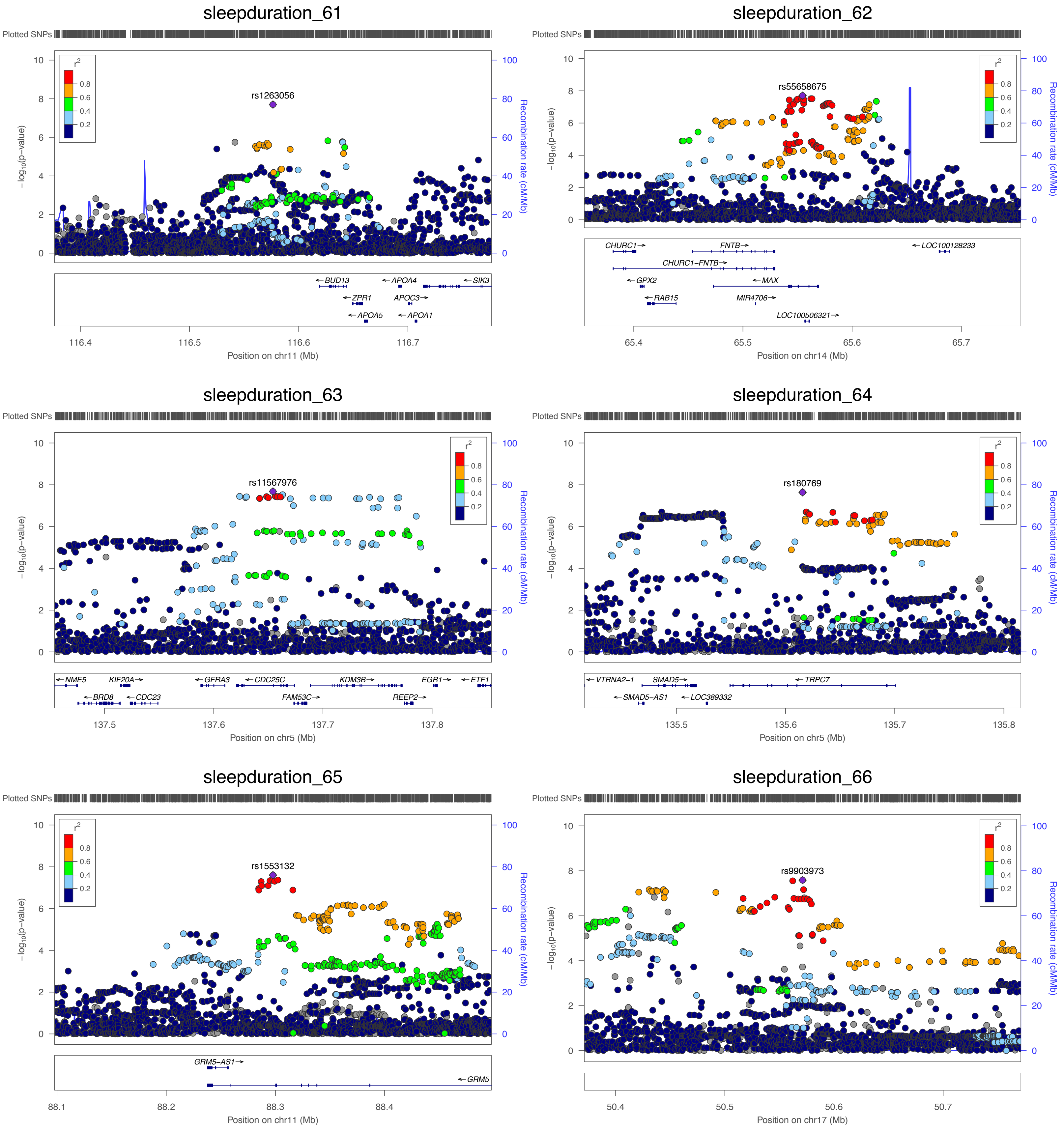
sleepduration_61
Plotted SNPs
rs1263056
BUD13
APOA4
SIK3
ZPR1
APOC3
APOA5
APOA1
Position on chr11 (Mb)
sleepduration_62
Plotted SNPs
rs55658675
CHURC1
FNTB
LOC100128233
CHURC1−FNTB
GPX2
MAX
RAB15
MIR4706
LOC100506321
Position on chr14 (Mb)
sleepduration_63
Plotted SNPs
rs11567976
NME5
KIF20A
GFRA3
CDC25C
KDM3B
EGR1
ETF1
BRD8
CDC23
FAM53C
REEP2
Position on chr5 (Mb)
sleepduration_64
Plotted SNPs
rs180769
VTRNA2−1
SMAD5
TRPC7
SMAD5−AS1
LOC389332
Position on chr5 (Mb)
sleepduration_65
Plotted SNPs
rs1553132
GRM5−AS1
GRM5
Position on chr11 (Mb)
sleepduration_66
Plotted SNPs
rs9903973
Position on chr17 (Mb)
−log10(p−value)
Recombination rate (cM/Mb)
r2

sleepduration_67

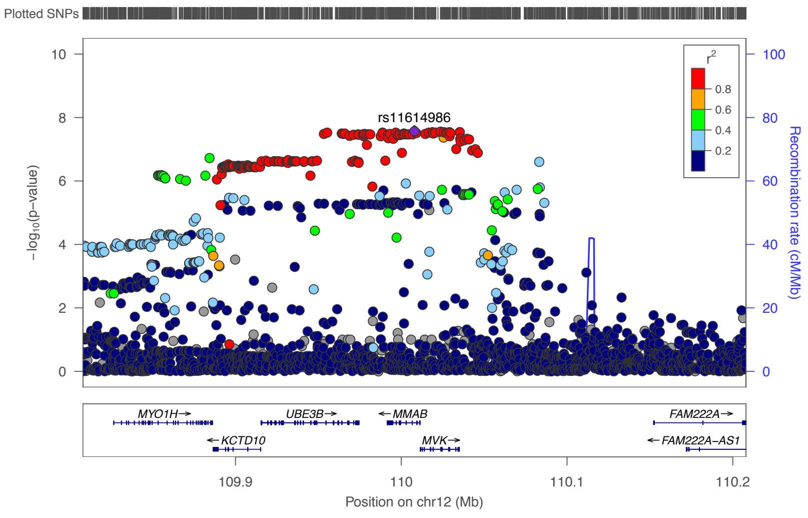

sleepduration_68

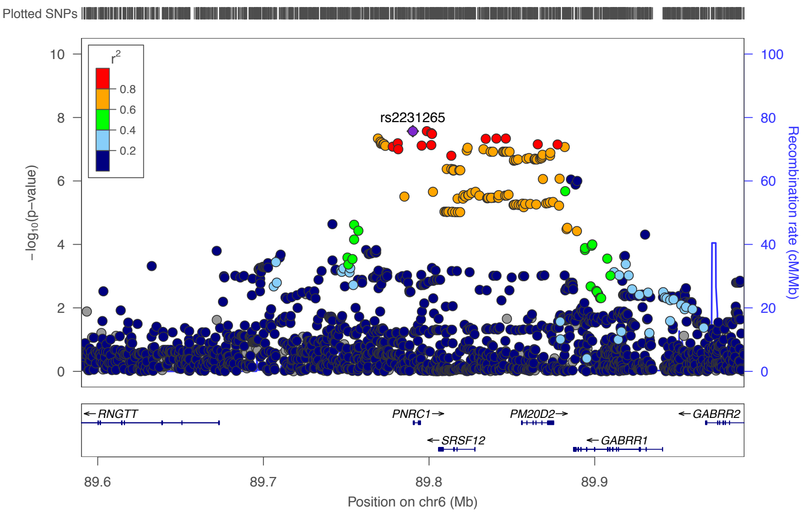

sleepduration_69

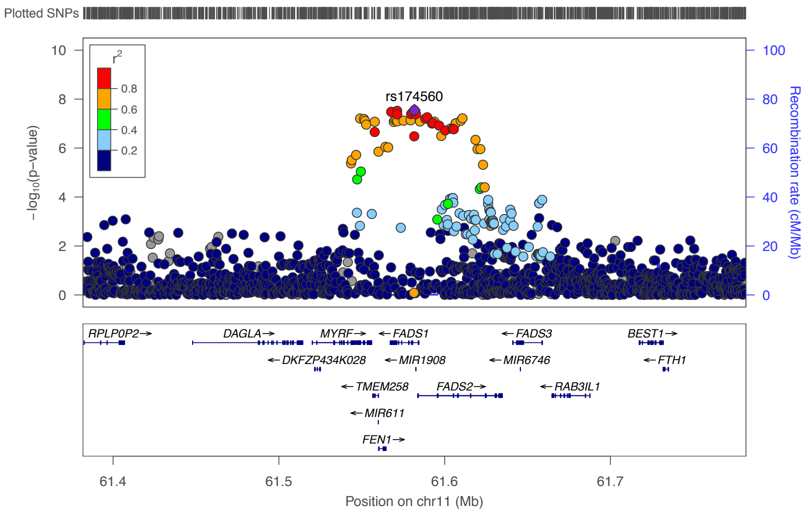

sleepduration_70

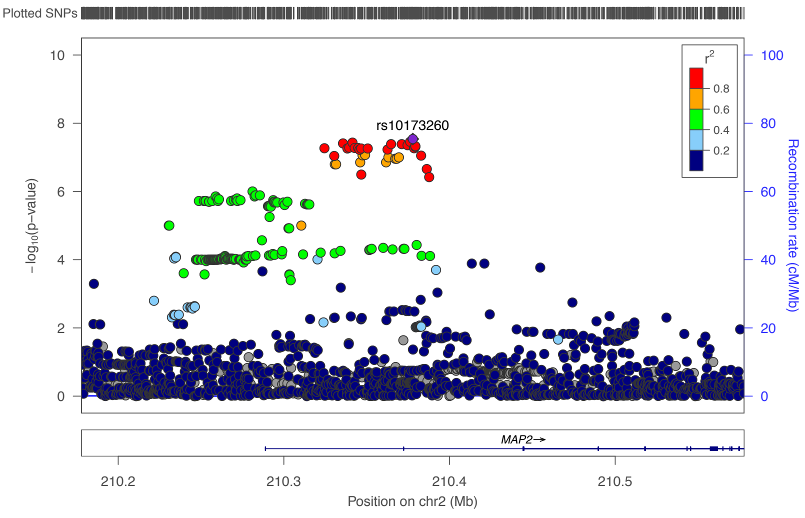

sleepduration_71

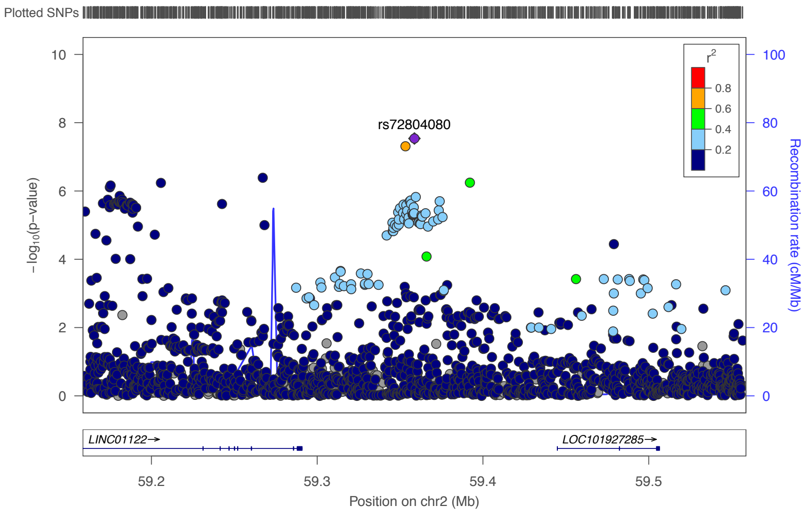

sleepduration_72

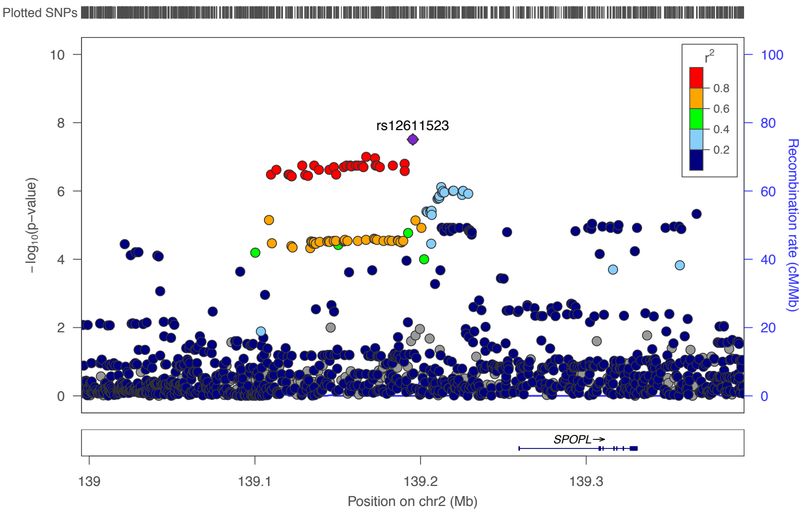

sleepduration_73

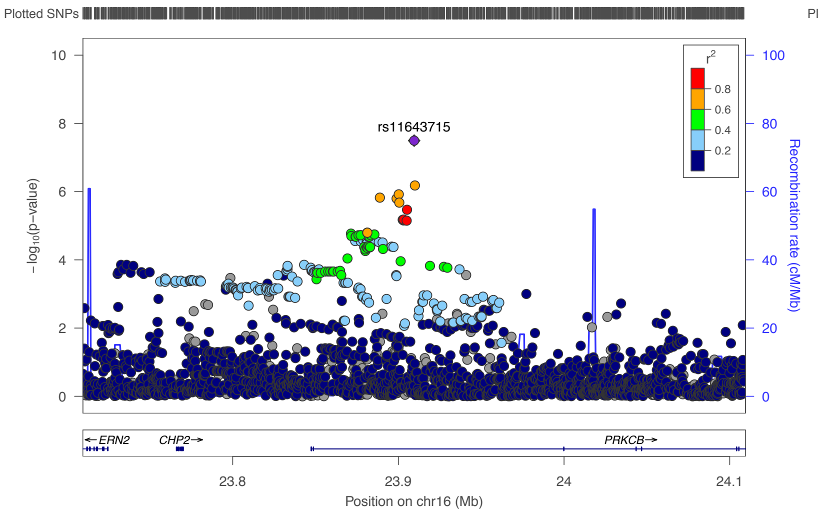

sleepduration_74

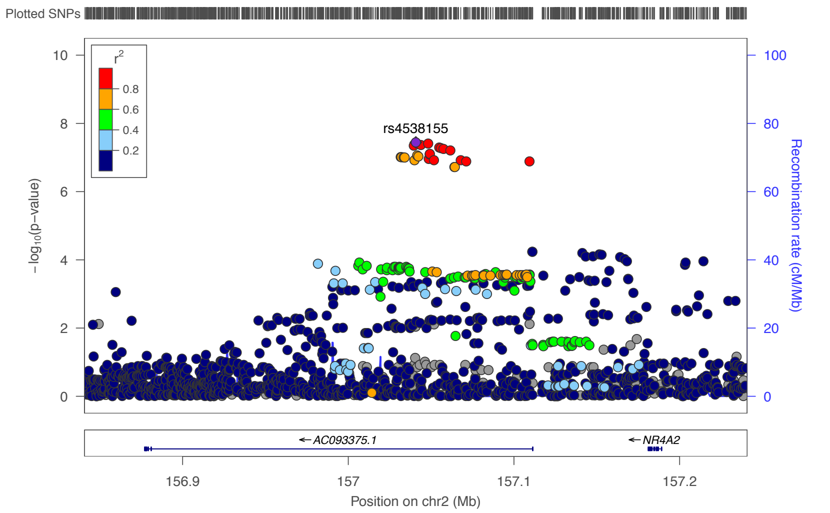

sleepduration_75

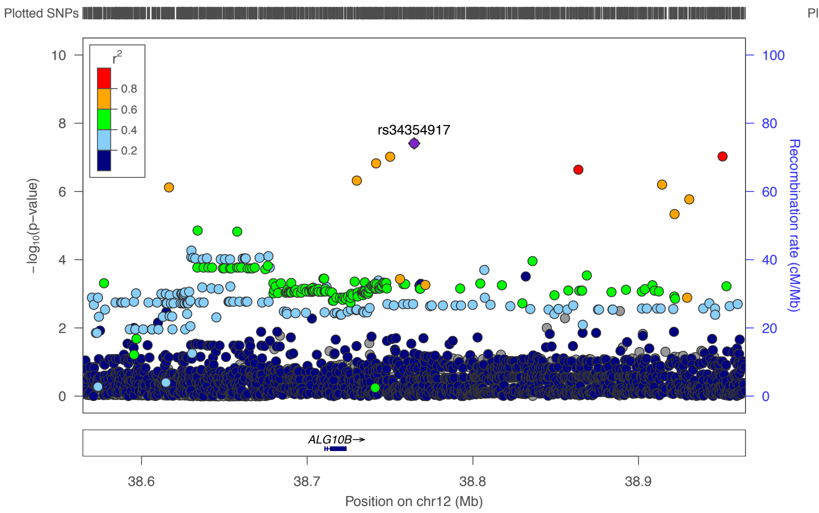

sleepduration_76

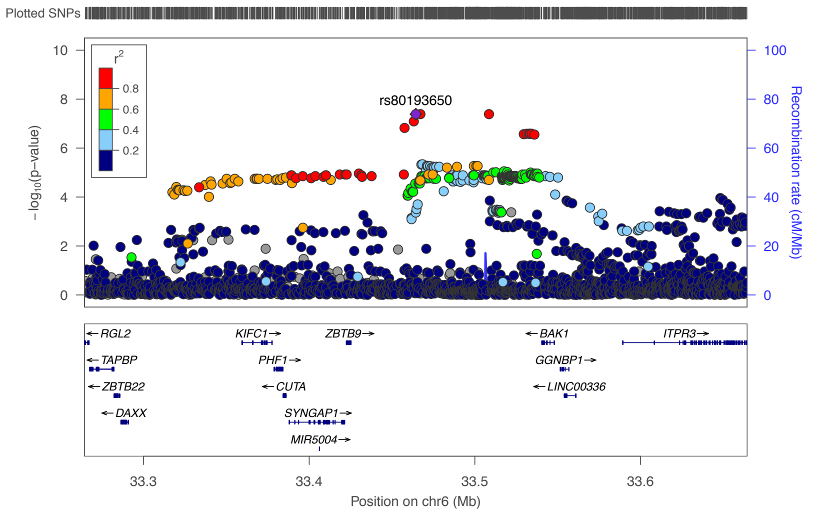

sleepduration_77

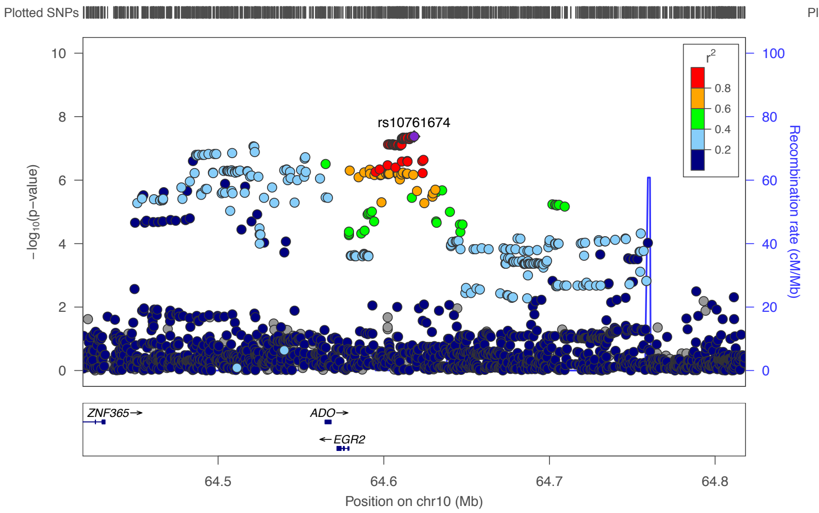

sleepduration_78

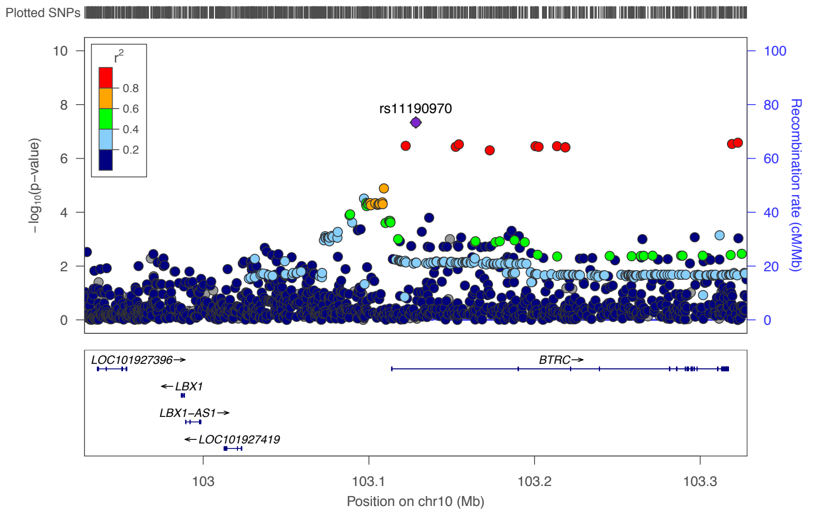

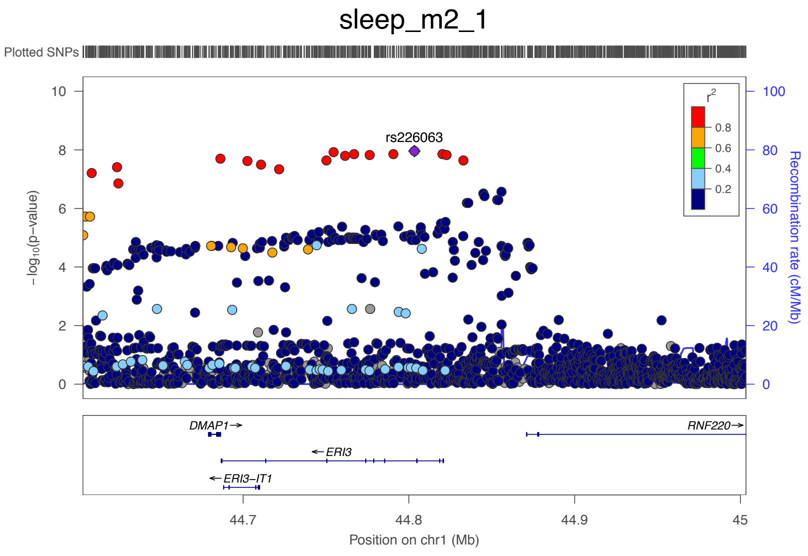
sleep_m2_1
Plotted SNPs
rs226063
r2
0.8
0.6
0.4
0.2
−log10(p−value)
Recombination rate (cM/Mb)
DMAP1→
RNF220→
←ERI3
←ERI3−IT1
44.7
44.8
44.9
45
Position on chr1 (Mb)

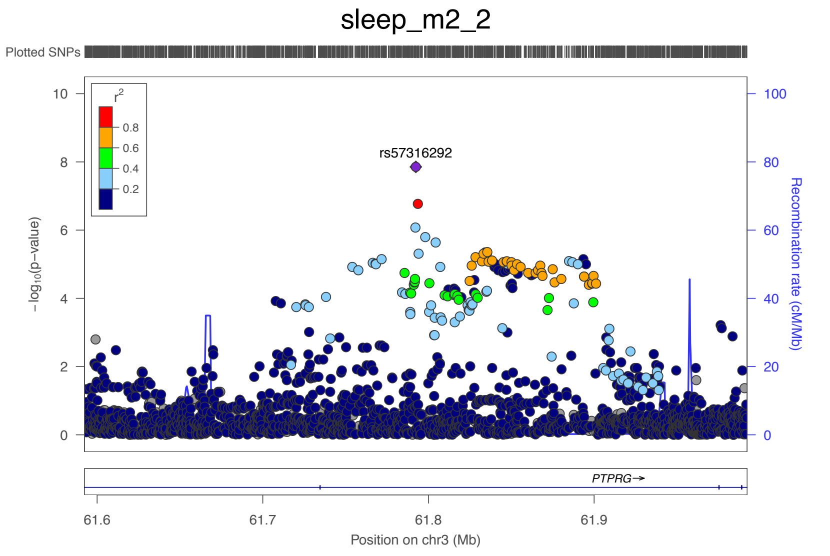
sleep_m2_2
Plotted SNPs
rs57316292
r2
0.8
0.6
0.4
0.2
−log10(p−value)
Recombination rate (cM/Mb)
PTPRG→
61.6
61.7
61.8
61.9
Position on chr3 (Mb)

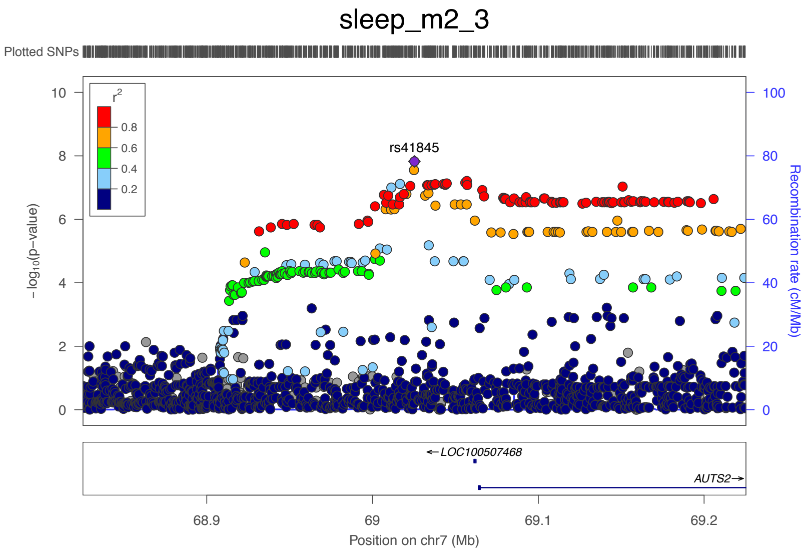
sleep_m2_3
Plotted SNPs
rs41845
r2
0.8
0.6
0.4
0.2
−log10(p−value)
Recombination rate (cM/Mb)
←LOC100507468
AUTS2→
68.9
69
69.1
69.2
Position on chr7 (Mb)

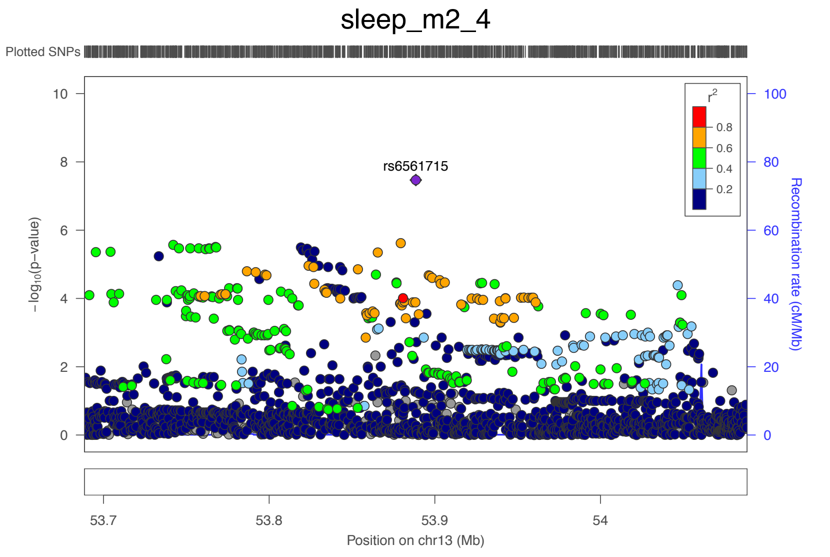
sleep_m2_4
Plotted SNPs
rs6561715
r2
0.8
0.6
0.4
0.2
−log10(p−value)
Recombination rate (cM/Mb)
53.7
53.8
53.9
54
Position on chr13 (Mb)

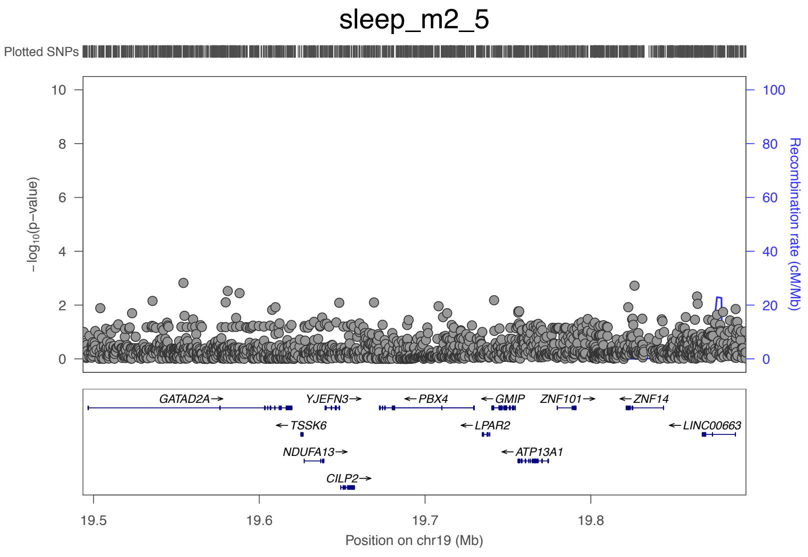
sleep_m2_5
Plotted SNPs
−log10(p−value)
Recombination rate (cM/Mb)
GATAD2A→
YJEFN3→
←PBX4
←GMIP
ZNF101→
←ZNF14
←TSSK6
←LPAR2
←LINC00663
NDUFA13→
←ATP13A1
CILP2→
19.5
19.6
19.7
19.8
Position on chr19 (Mb)

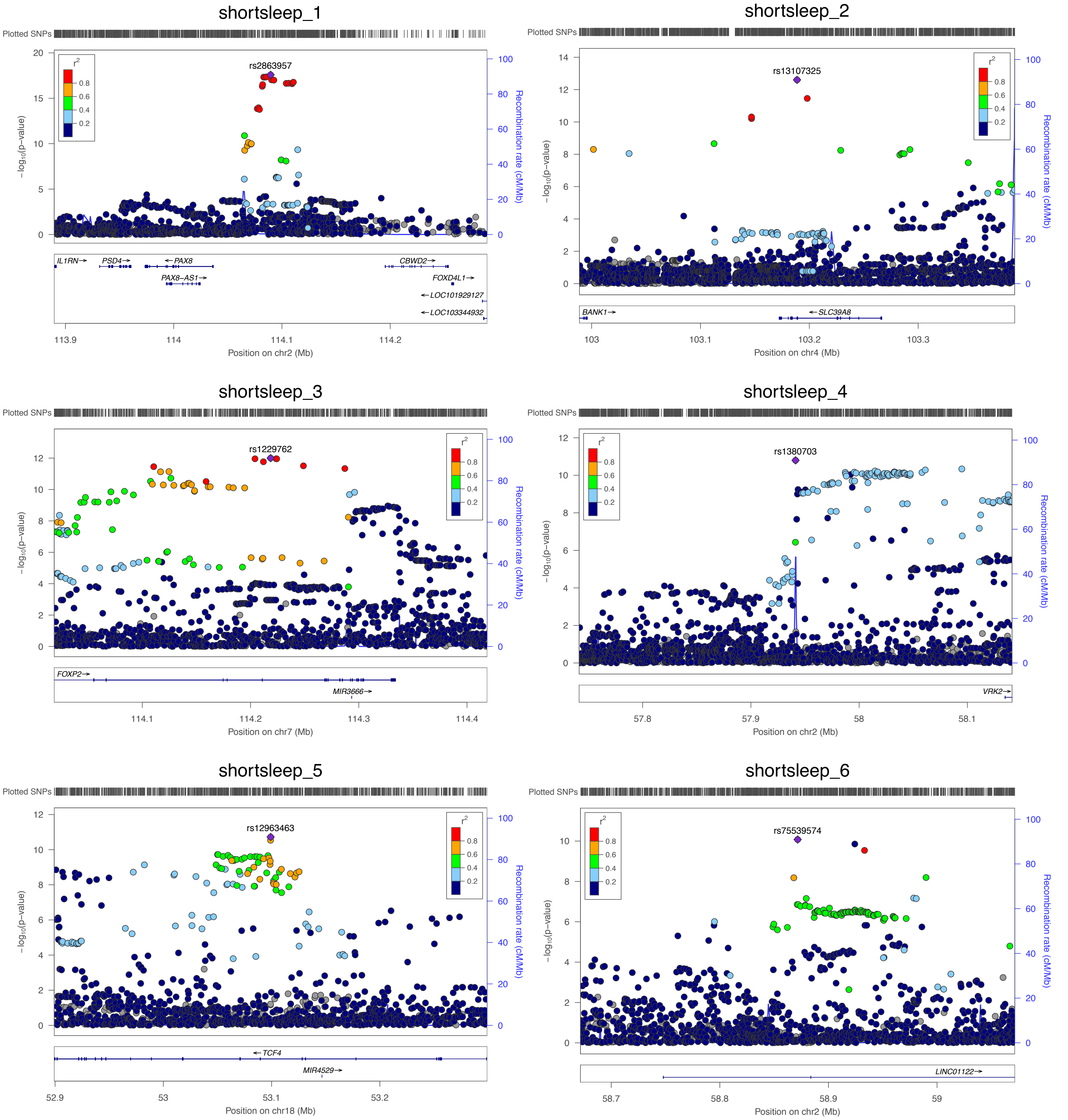

shortsleep_1
rs2863957
IL1RN→
PSD4→
←PAX8
PAX8−AS1→
CBWD2→
FOXD4L1→
←LOC101929127
←LOC103344932
Position on chr2 (Mb)
shortsleep_2
rs13107325
BANK1→
←SLC39A8
Position on chr4 (Mb)
shortsleep_3
rs1229762
FOXP2→
MIR3666→
Position on chr7 (Mb)
shortsleep_4
rs1380703
VRK2→
Position on chr2 (Mb)
shortsleep_5
rs12963463
←TCF4
MIR4529→
Position on chr18 (Mb)
shortsleep_6
rs75539574
LINC01122→
Position on chr2 (Mb)
Plotted SNPs
−log10(p−value)
Recombination rate (cM/Mb)
r2

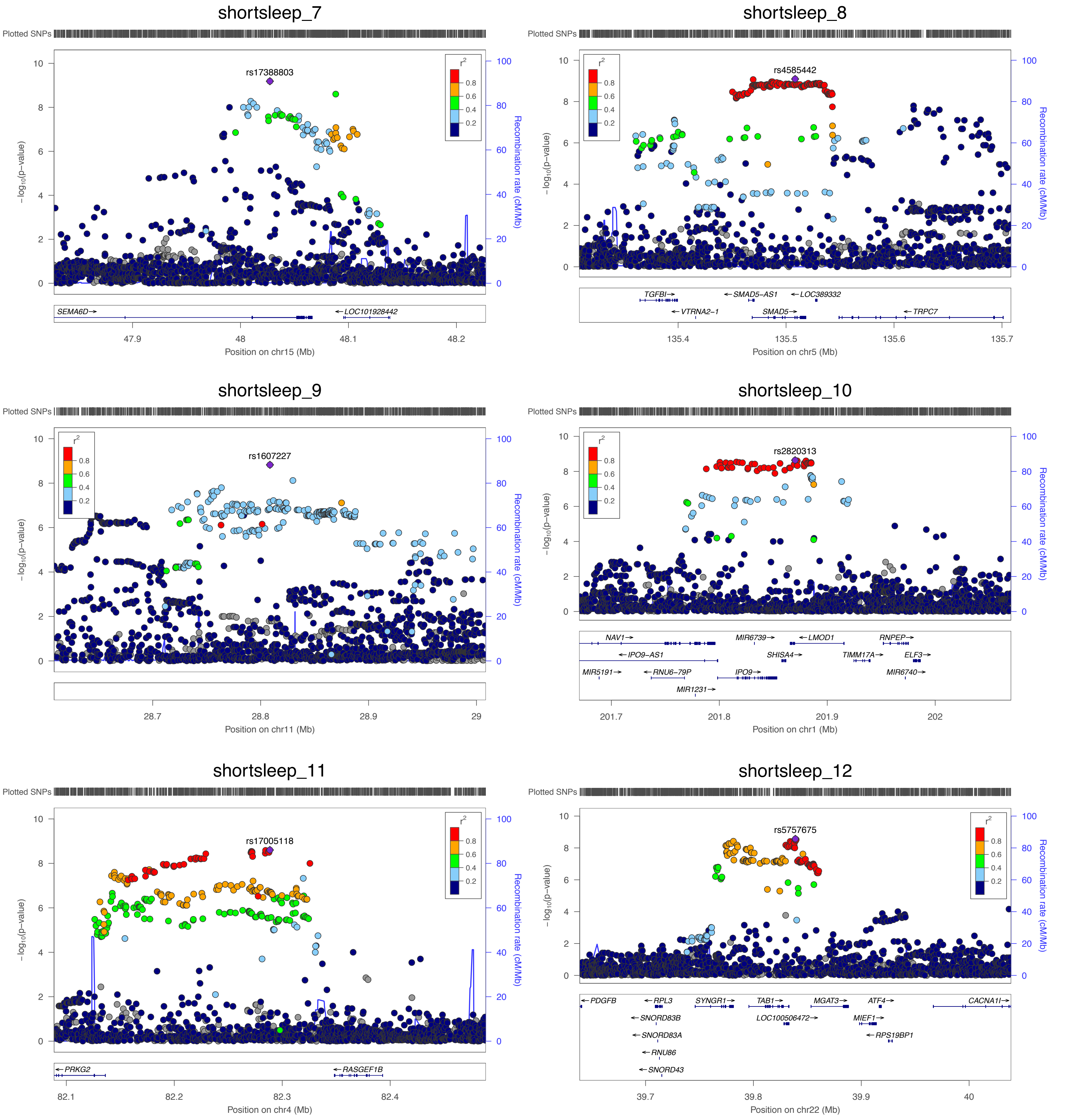

shortsleep_7
Plotted SNPs
rs17388803
SEMA6D→
←LOC101928442
Position on chr15 (Mb)
shortsleep_8
rs4585442
TGFBI→
←SMAD5−AS1
←LOC389332
←VTRNA2−1
SMAD5→
←TRPC7
Position on chr5 (Mb)
shortsleep_9
rs1607227
Position on chr11 (Mb)
shortsleep_10
rs2820313
NAV1→
MIR6739→
←LMOD1
RNPEP→
←IPO9−AS1
SHISA4→
TIMM17A→
ELF3→
MIR5191→
←RNU6−79P
IPO9→
MIR6740→
MIR1231→
Position on chr1 (Mb)
shortsleep_11
rs17005118
←PRKG2
←RASGEF1B
Position on chr4 (Mb)
shortsleep_12
rs5757675
←PDGFB
←RPL3
SYNGR1→
TAB1→
MGAT3→
ATF4→
CACNA1I→
←SNORD83B
LOC100506472→
MIEF1→
←SNORD83A
←RPS19BP1
←RNU86
←SNORD43
Position on chr22 (Mb)
−log10(p−value)
Recombination rate (cM/Mb)
r2

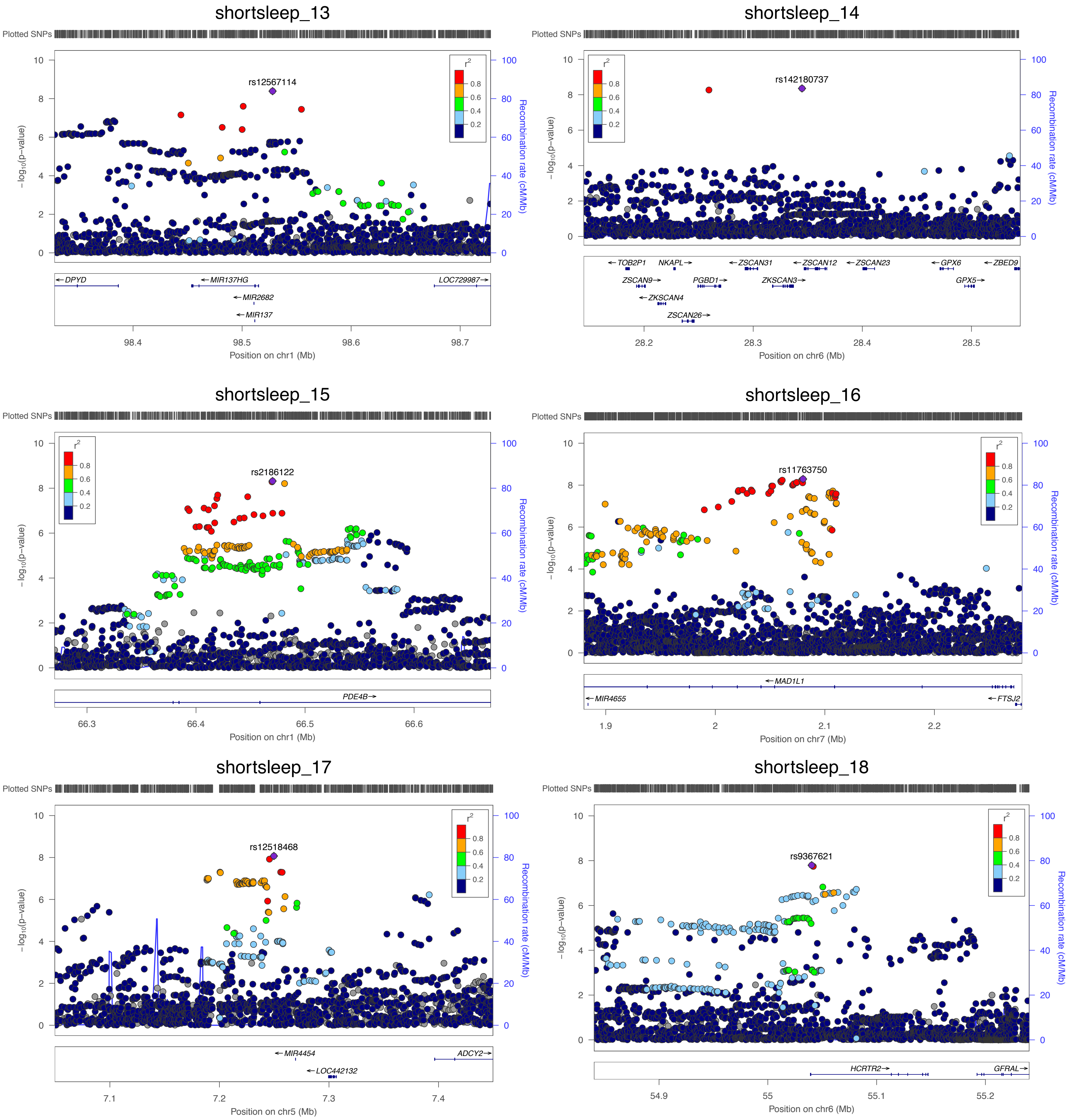

shortsleep_13
Plotted SNPs
rs12567114
DPYD
MIR137HG
MIR2682
MIR137
LOC729987
Position on chr1 (Mb)
shortsleep_14
Plotted SNPs
rs142180737
TOB2P1
NKAPL
ZSCAN31
ZSCAN12
ZSCAN23
GPX6
ZBED9
ZSCAN9
PGBD1
ZKSCAN3
GPX5
ZKSCAN4
ZSCAN26
Position on chr6 (Mb)
shortsleep_15
Plotted SNPs
rs2186122
PDE4B
Position on chr1 (Mb)
shortsleep_16
Plotted SNPs
rs11763750
MAD1L1
MIR4655
FTSJ2
Position on chr7 (Mb)
shortsleep_17
Plotted SNPs
rs12518468
MIR4454
ADCY2
LOC442132
Position on chr5 (Mb)
shortsleep_18
Plotted SNPs
rs9367621
HCRTR2
GFRAL
Position on chr6 (Mb)
−log10(p−value)
Recombination rate (cM/Mb)
r2

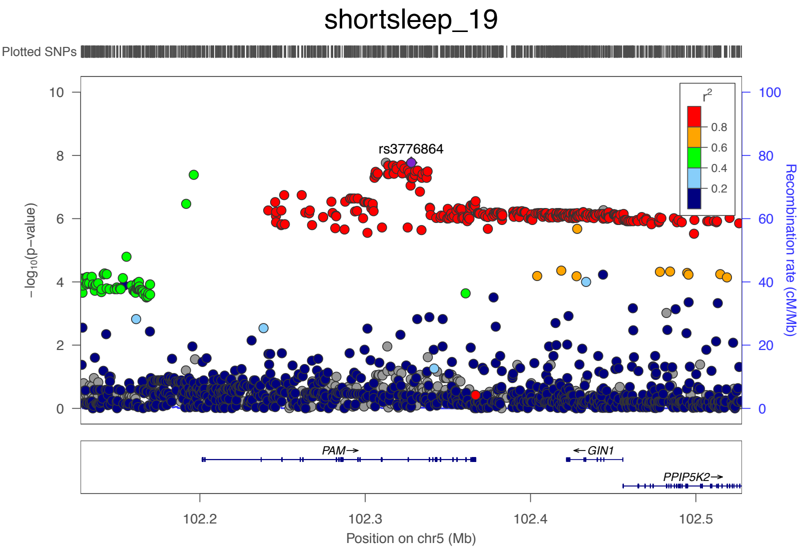
shortsleep_19
Plotted SNPs
rs3776864
r²
−log10(p−value)
Recombination rate (cM/Mb)
PAM→
←GIN1
PPIP5K2→
102.2
102.3
102.4
102.5
Position on chr5 (Mb)

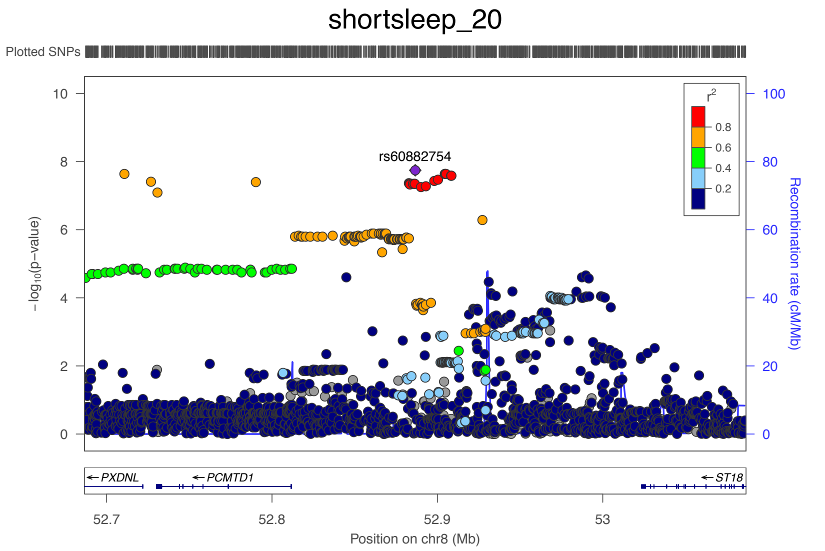
shortsleep_20
Plotted SNPs
rs60882754
r²
−log10(p−value)
Recombination rate (cM/Mb)
←PXDNL
←PCMTD1
←ST18
52.7
52.8
52.9
53
Position on chr8 (Mb)

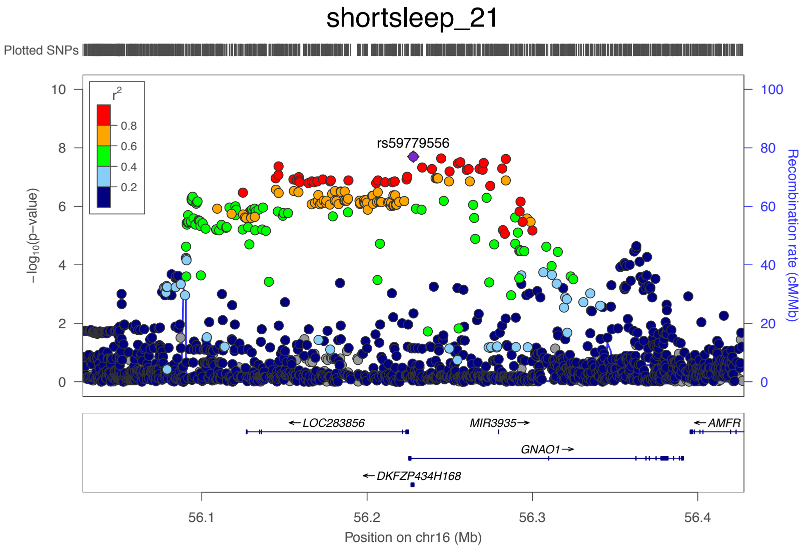
shortsleep_21
Plotted SNPs
rs59779556
r²
−log10(p−value)
Recombination rate (cM/Mb)
←LOC283856
MIR3935→
←AMFR
GNAO1→
←DKFZP434H168
56.1
56.2
56.3
56.4
Position on chr16 (Mb)

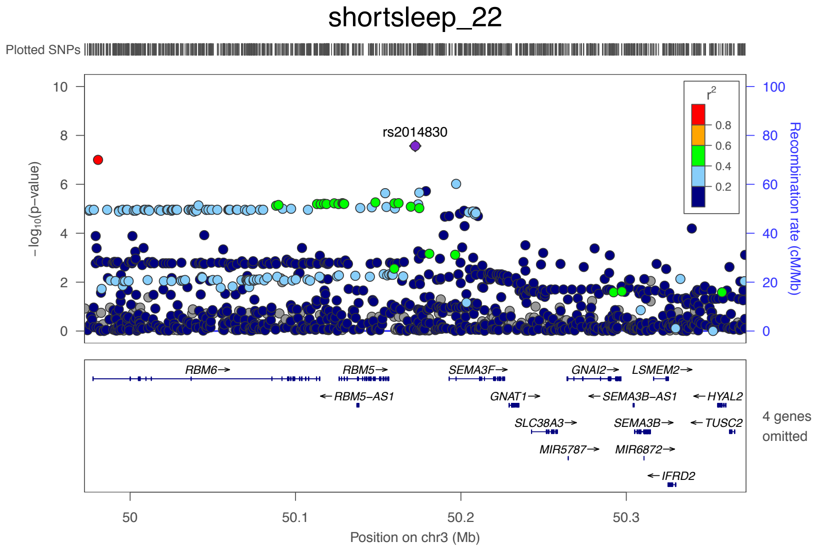
shortsleep_22
Plotted SNPs
rs2014830
r²
−log10(p−value)
Recombination rate (cM/Mb)
RBM6→
RBM5→
SEMA3F→
GNAI2→
LSMEM2→
←RBM5−AS1
GNAT1→
←SEMA3B−AS1
←HYAL2
SLC38A3→
SEMA3B→
←TUSC2
MIR5787→
MIR6872→
←IFRD2
4 genes omitted
50
50.1
50.2
50.3
Position on chr3 (Mb)

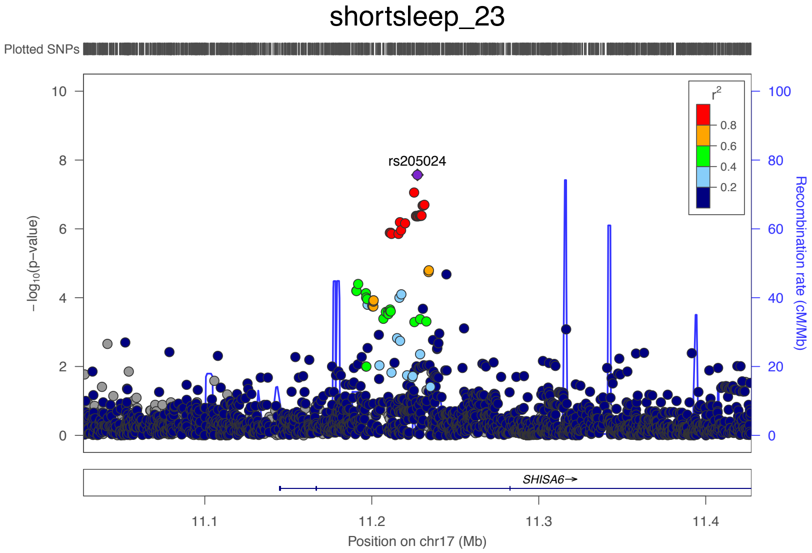
shortsleep_23
Plotted SNPs
rs205024
r²
−log10(p−value)
Recombination rate (cM/Mb)
SHISA6→
11.1
11.2
11.3
11.4
Position on chr17 (Mb)

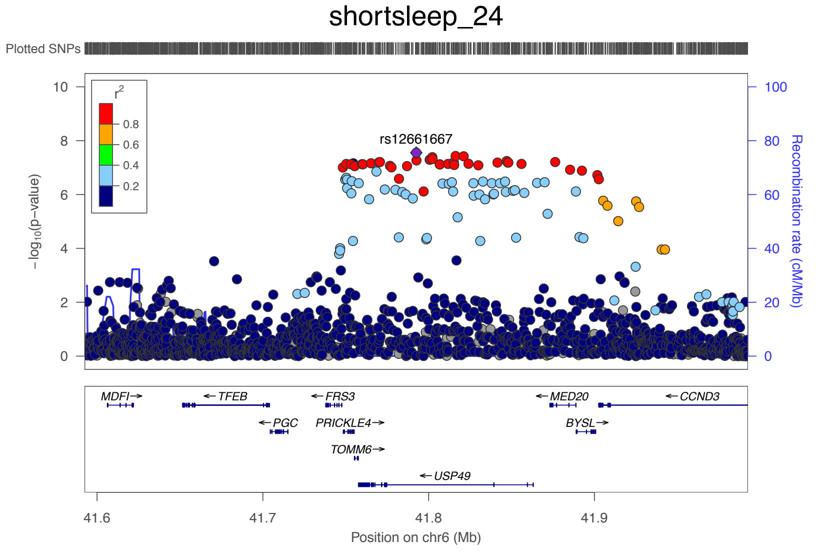
shortsleep_24
Plotted SNPs
rs12661667
r²
−log10(p−value)
Recombination rate (cM/Mb)
MDFI→
←TFEB
←FRS3
←MED20
←CCND3
←PGC
PRICKLE4→
BYSL→
TOMM6→
←USP49
41.6
41.7
41.8
41.9
Position on chr6 (Mb)

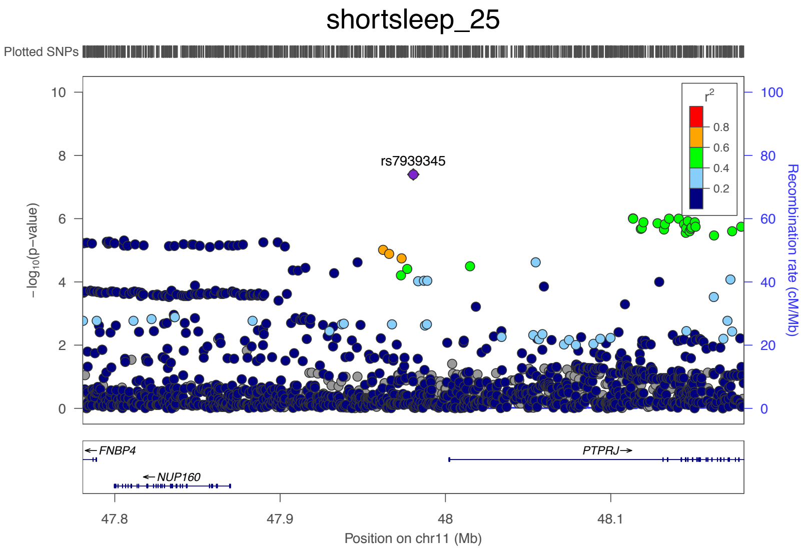
shortsleep_25
Plotted SNPs
rs7939345
r²
0.8
0.6
0.4
0.2
−log10(p−value)
Recombination rate (cM/Mb)
FNBP4
NUP160
PTPRJ
47.8
47.9
48
48.1
Position on chr11 (Mb)

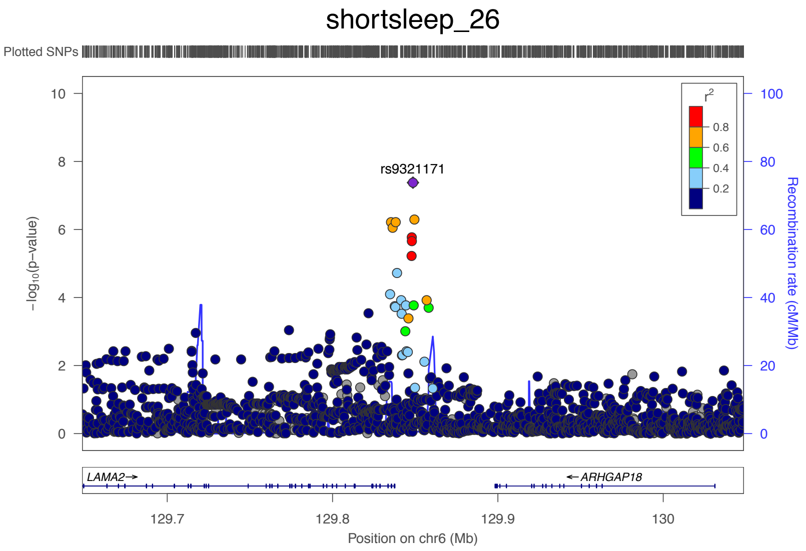
shortsleep_26
Plotted SNPs
rs9321171
r²
0.8
0.6
0.4
0.2
−log10(p−value)
Recombination rate (cM/Mb)
LAMA2
ARHGAP18
129.7
129.8
129.9
130
Position on chr6 (Mb)

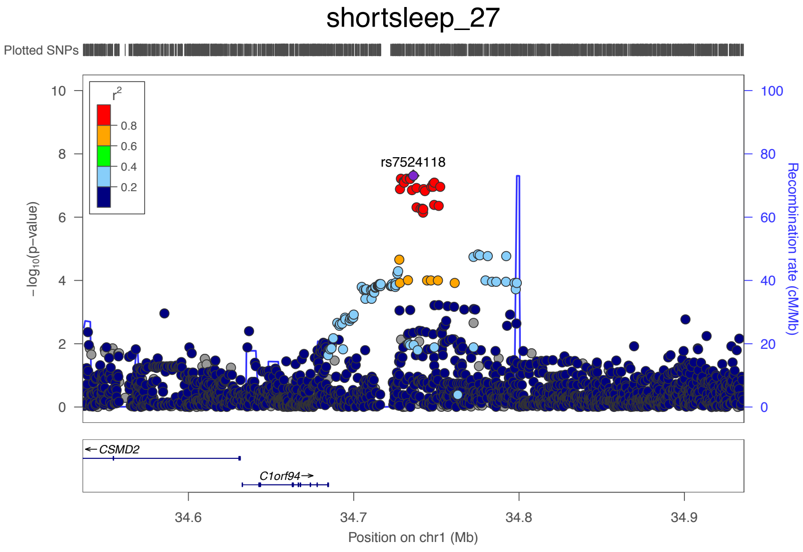
shortsleep_27
Plotted SNPs
rs7524118
r²
0.8
0.6
0.4
0.2
−log10(p−value)
Recombination rate (cM/Mb)
CSMD2
C1orf94
34.6
34.7
34.8
34.9
Position on chr1 (Mb)

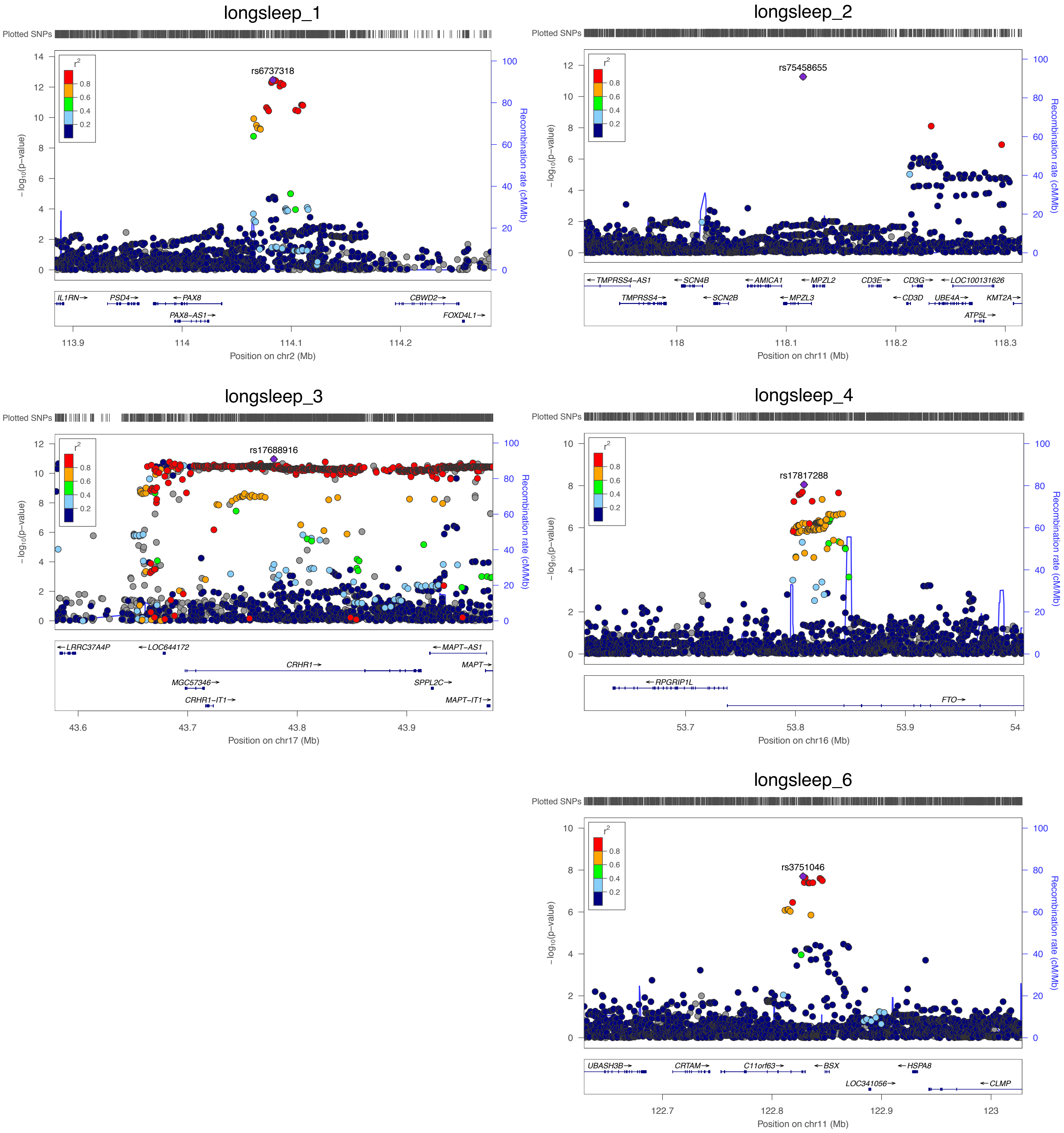

longsleep_1
Plotted SNPs
rs6737318
r2
0.8
0.6
0.4
0.2
−log10(p−value)
Recombination rate (cM/Mb)
IL1RN
PSD4
PAX8
PAX8−AS1
CBWD2
FOXD4L1
113.9
114
114.1
114.2
Position on chr2 (Mb)
longsleep_2
Plotted SNPs
rs75458655
TMPRSS4−AS1
SCN4B
AMICA1
MPZL2
CD3E
CD3G
LOC100131626
TMPRSS4
SCN2B
MPZL3
CD3D
UBE4A
KMT2A
ATP5L
118
118.1
118.2
118.3
Position on chr11 (Mb)
longsleep_3
Plotted SNPs
rs17688916
LRRC37A4P
LOC644172
MAPT−AS1
CRHR1
MAPT
MGC57346
SPPL2C
CRHR1−IT1
MAPT−IT1
43.6
43.7
43.8
43.9
Position on chr17 (Mb)
longsleep_4
Plotted SNPs
rs17817288
RPGRIP1L
FTO
53.7
53.8
53.9
54
Position on chr16 (Mb)
longsleep_6
Plotted SNPs
rs3751046
UBASH3B
CRTAM
C11orf63
BSX
HSPA8
LOC341056
CLMP
122.7
122.8
122.9
123
Position on chr11 (Mb)

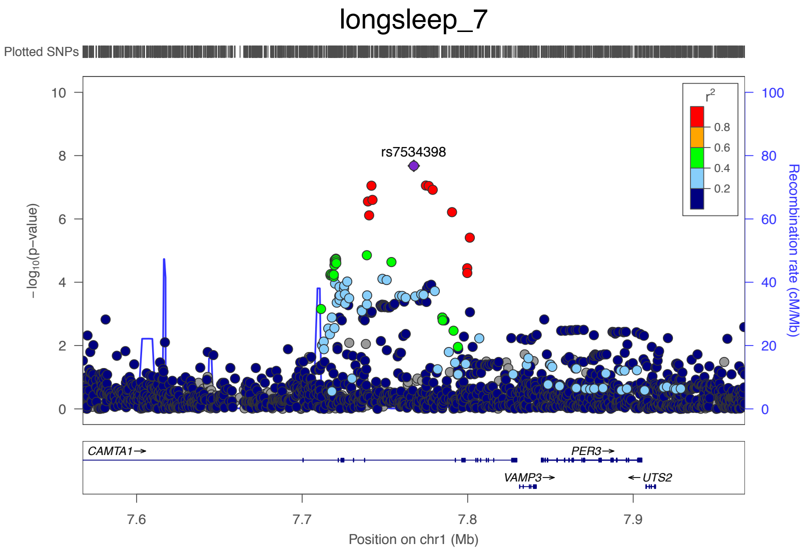
longsleep_7
Plotted SNPs
rs7534398
r²
0.8
0.6
0.4
0.2
−log10(p−value)
Recombination rate (cM/Mb)
CAMTA1→
PER3→
VAMP3→
←UTS2
Position on chr1 (Mb)

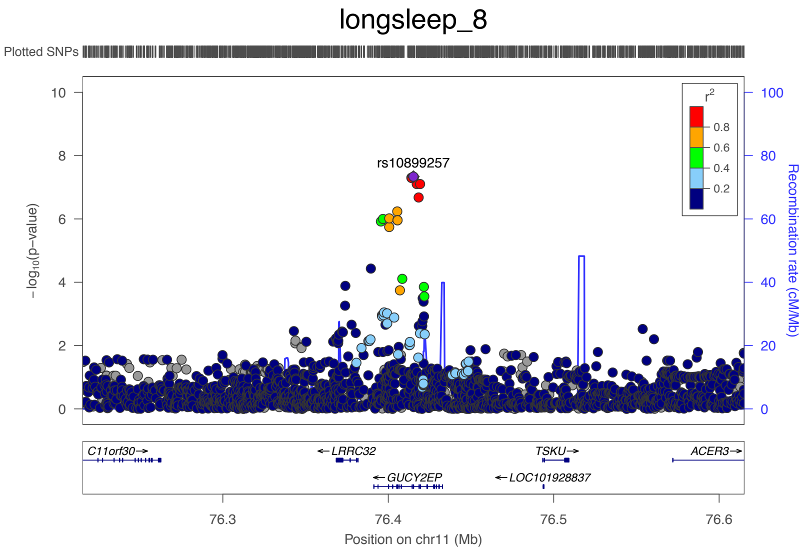
longsleep_8
Plotted SNPs
rs10899257
r²
0.8
0.6
0.4
0.2
−log10(p−value)
Recombination rate (cM/Mb)
C11orf30→
←LRRC32
TSKU→
ACER3→
←GUCY2EP
←LOC101928837
Position on chr11 (Mb)

ma_adult_1

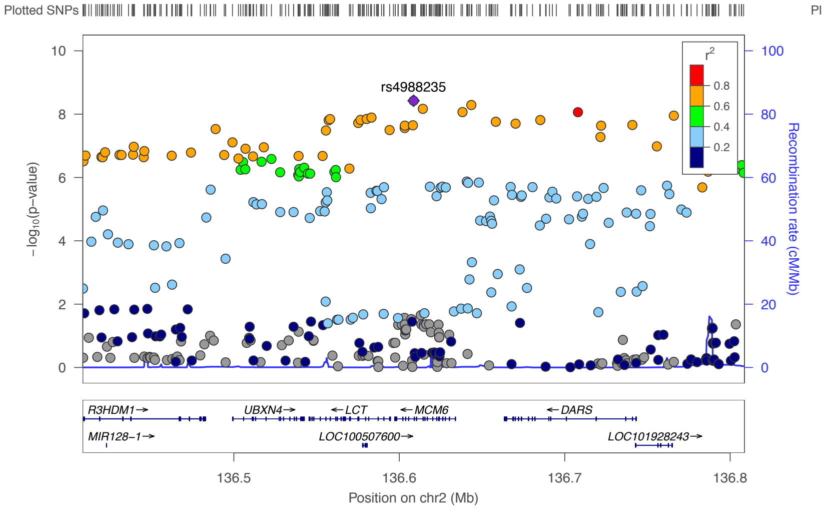

ma_adult_2

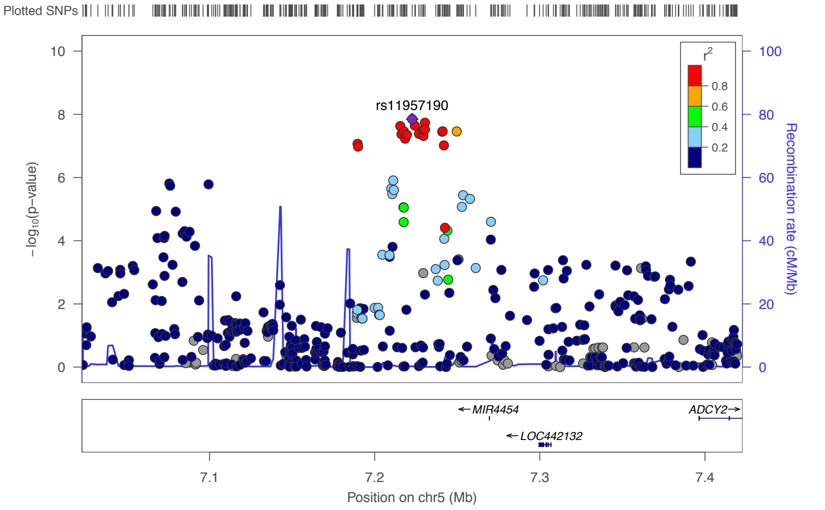

ma_adult_3

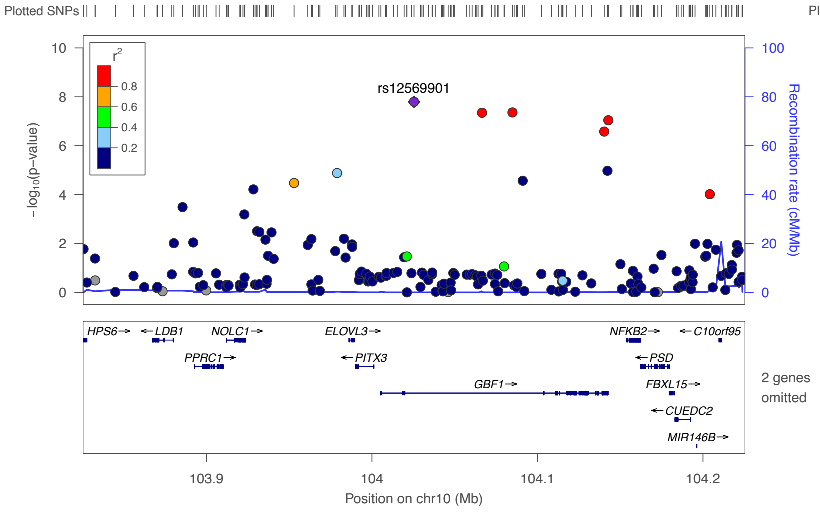

ma_adult_4

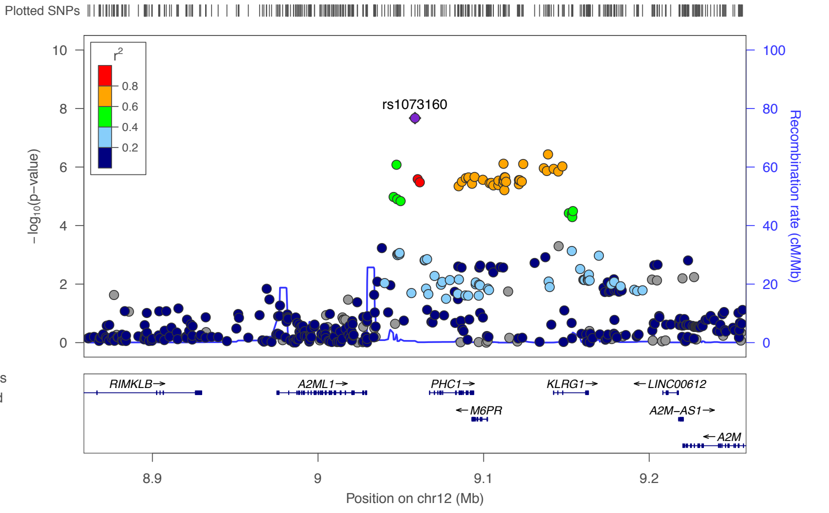

ma_adult_5

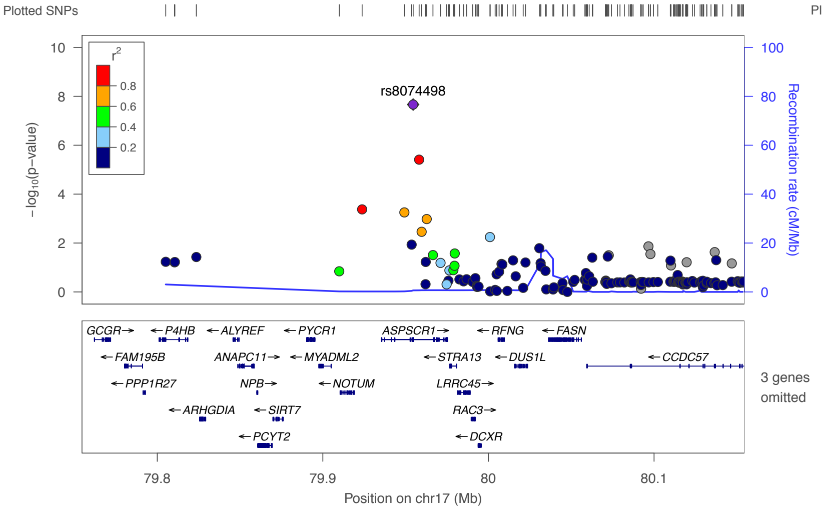

ma_adult_6

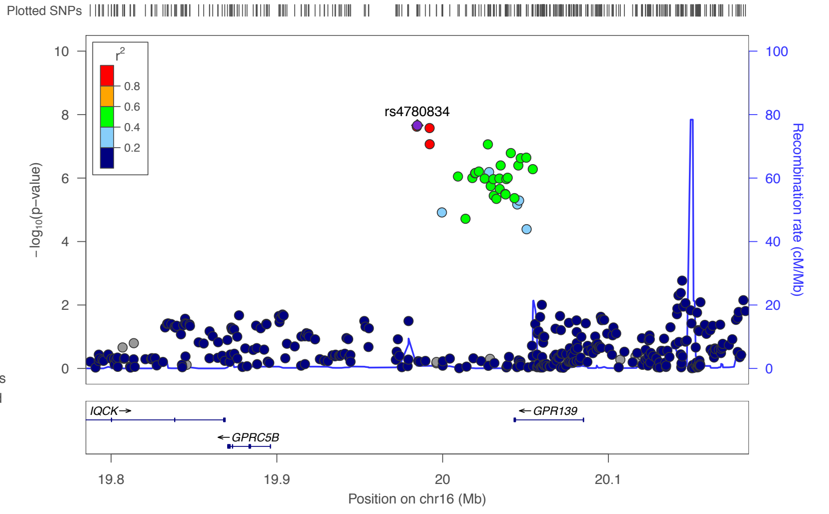

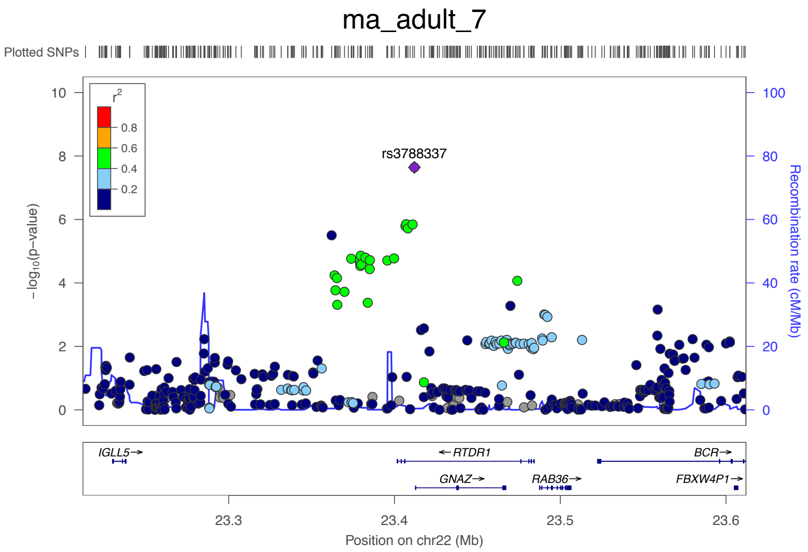
ma_adult_7
Plotted SNPs
rs3788337
IGLL5
RTDR1
BCR
GNAZ
RAB36
FBXW4P1
−log10(p−value)
Recombination rate (cM/Mb)
Position on chr22 (Mb)

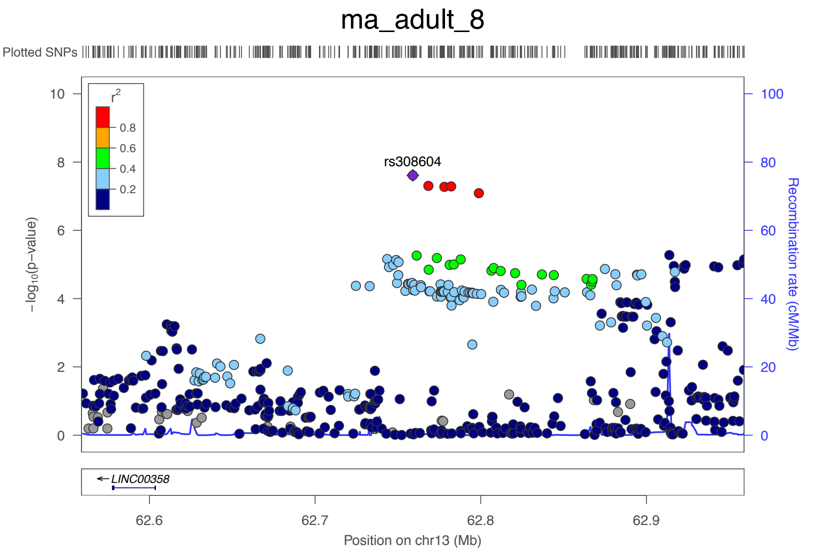
ma_adult_8
Plotted SNPs
rs308604
LINC00358
−log10(p−value)
Recombination rate (cM/Mb)
Position on chr13 (Mb)

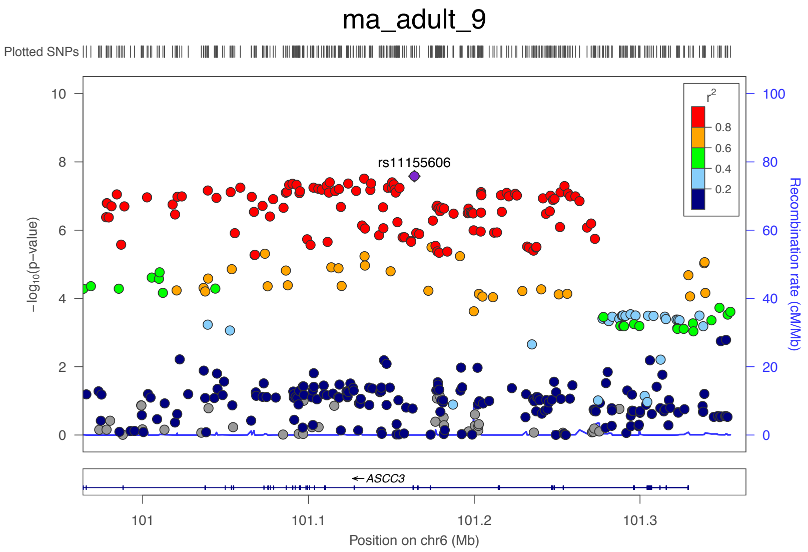
ma_adult_9
Plotted SNPs
rs11155606
ASCC3
−log10(p−value)
Recombination rate (cM/Mb)
Position on chr6 (Mb)

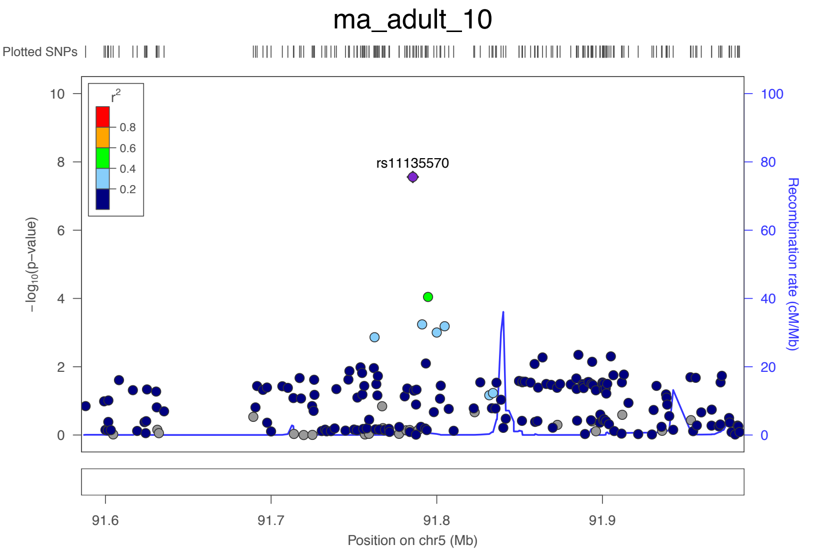
ma_adult_10
Plotted SNPs
rs11135570
−log10(p−value)
Recombination rate (cM/Mb)
Position on chr5 (Mb)

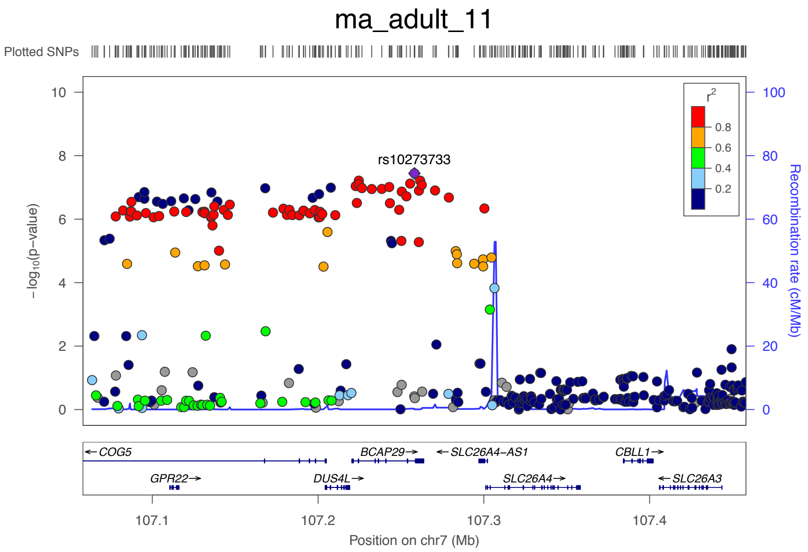
ma_adult_11
Plotted SNPs
rs10273733
COG5
BCAP29
SLC26A4−AS1
CBLL1
GPR22
DUS4L
SLC26A4
SLC26A3
−log10(p−value)
Recombination rate (cM/Mb)
Position on chr7 (Mb)

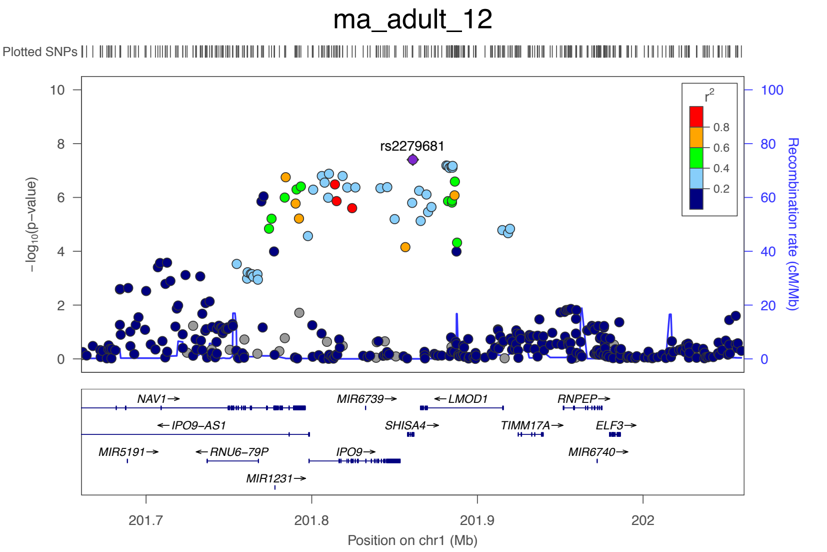
ma_adult_12
Plotted SNPs
rs2279681
NAV1
MIR6739
LMOD1
RNPEP
IPO9−AS1
SHISA4
TIMM17A
ELF3
MIR5191
RNU6−79P
IPO9
MIR6740
MIR1231
−log10(p−value)
Recombination rate (cM/Mb)
Position on chr1 (Mb)

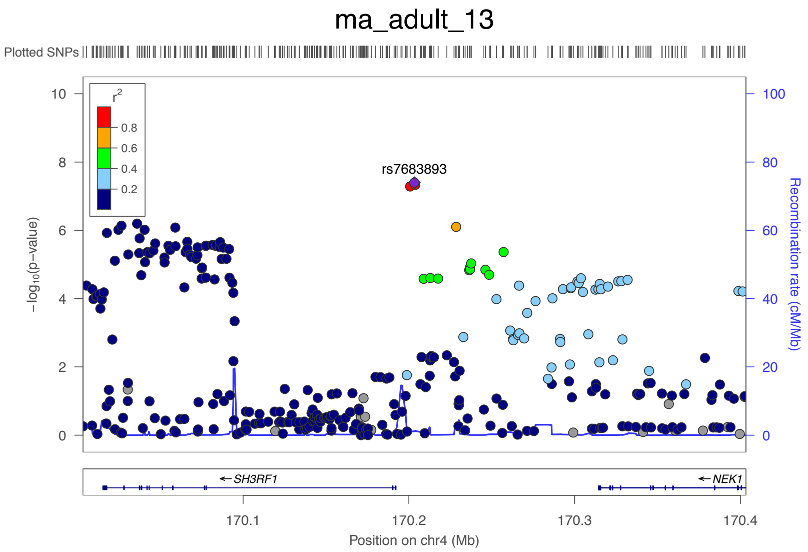

ma_adult_13
Plotted SNPs
rs7683893
r2
0.8
0.6
0.4
0.2
−log10(p−value)
Recombination rate (cM/Mb)
SH3RF1
NEK1
170.1
170.2
170.3
170.4
Position on chr4 (Mb)

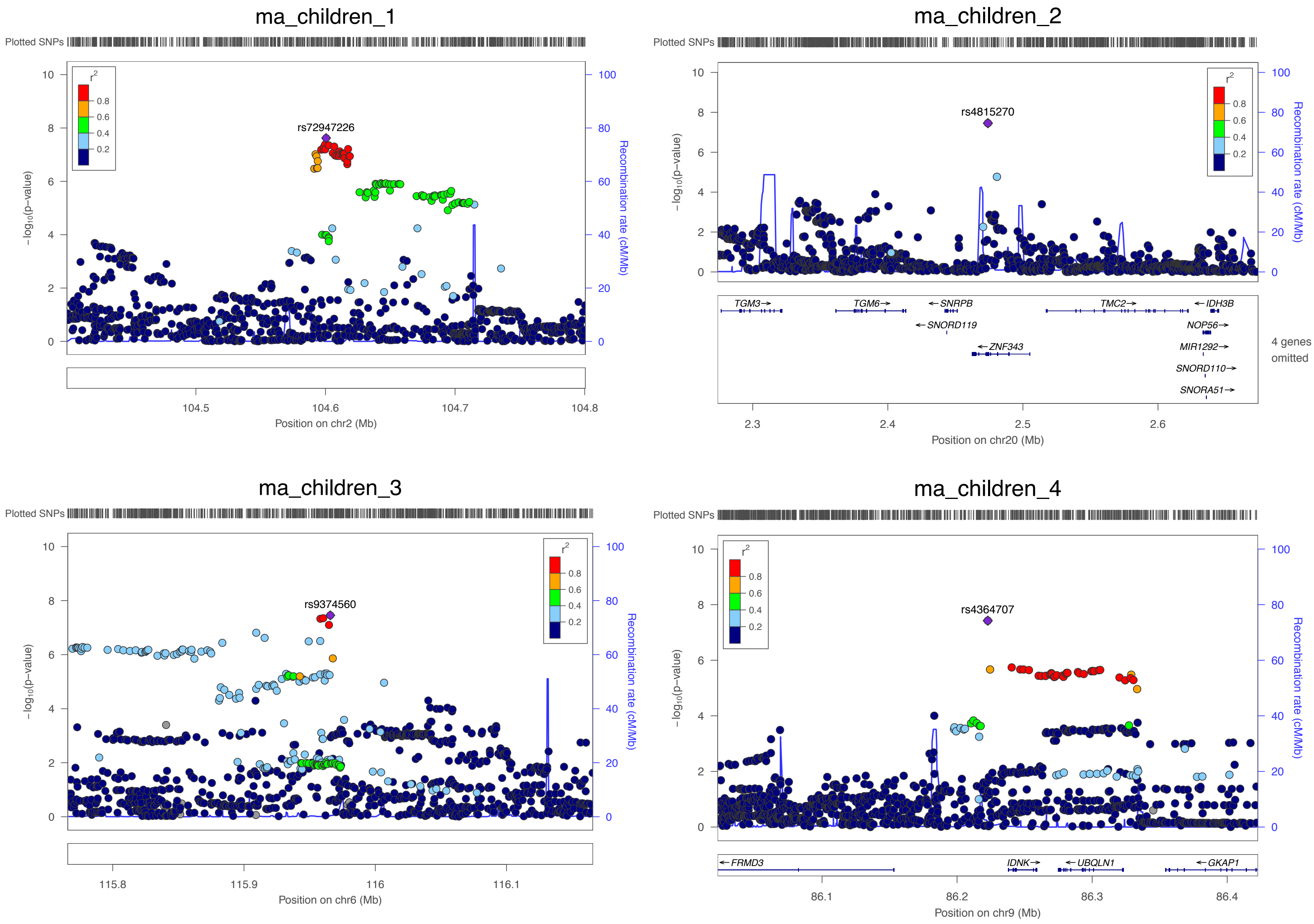
ma_children_1
Plotted SNPs
rs72947226
r²
0.8
0.6
0.4
0.2
−log10(p−value)
Recombination rate (cM/Mb)
104.5
104.6
104.7
104.8
Position on chr2 (Mb)
ma_children_2
Plotted SNPs
rs4815270
TGM3
TGM6
SNRPB
SNORD119
ZNF343
TMC2
IDH3B
NOP56
MIR1292
SNORD110
SNORA51
4 genes omitted
2.3
2.4
2.5
2.6
Position on chr20 (Mb)
ma_children_3
Plotted SNPs
rs9374560
115.8
115.9
116
116.1
Position on chr6 (Mb)
ma_children_4
Plotted SNPs
rs4364707
FRMD3
IDNK
UBQLN1
GKAP1
86.1
86.2
86.3
86.4
Position on chr9 (Mb)

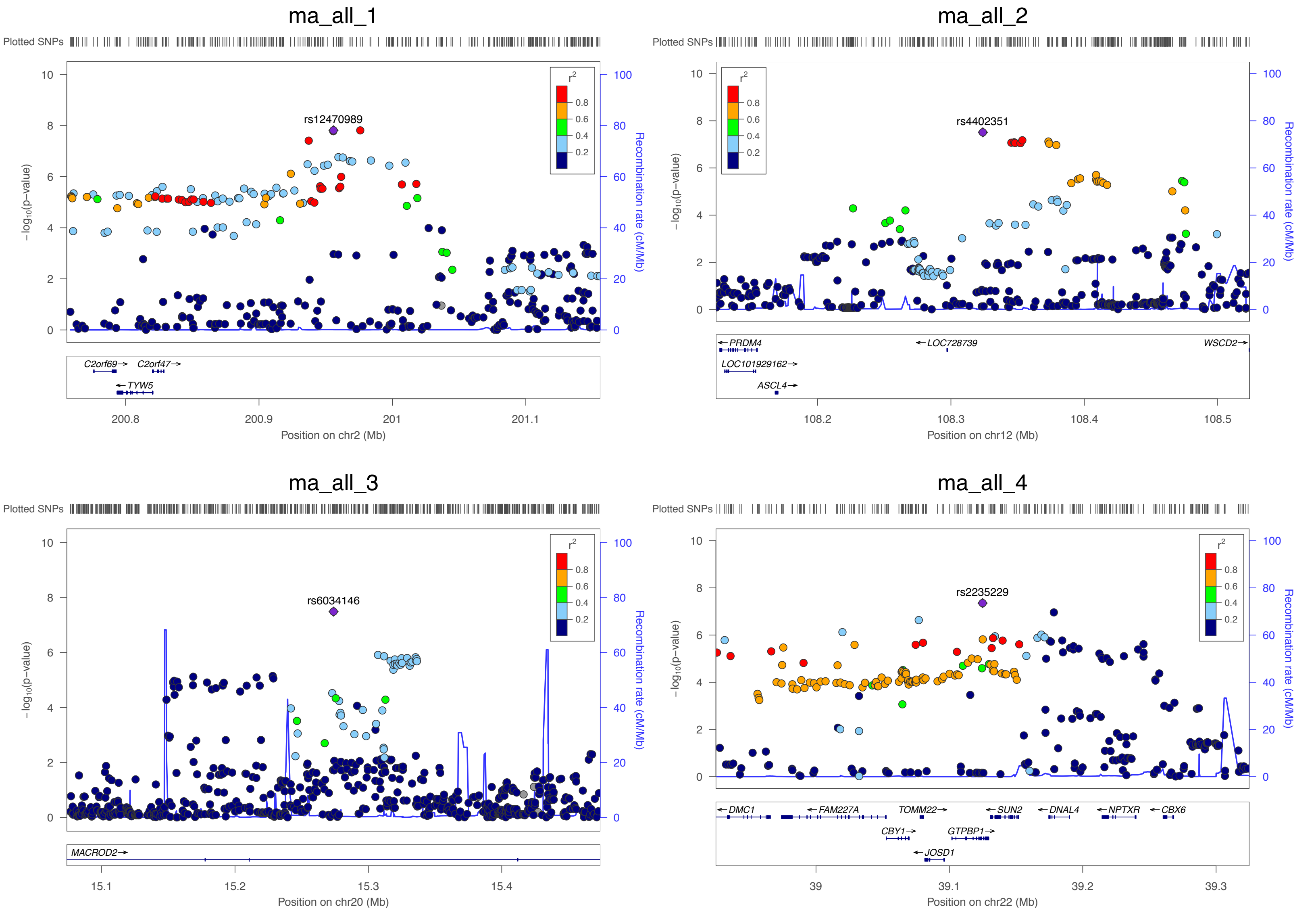
ma_all_1
Plotted SNPs
rs12470989
C2orf69
C2orf47
TYW5
Position on chr2 (Mb)
−log10(p−value)
Recombination rate (cM/Mb)
ma_all_2
Plotted SNPs
rs4402351
PRDM4
LOC728739
WSCD2
LOC101929162
ASCL4
Position on chr12 (Mb)
ma_all_3
Plotted SNPs
rs6034146
MACROD2
Position on chr20 (Mb)
ma_all_4
Plotted SNPs
rs2235229
DMC1
FAM227A
TOMM22
SUN2
DNAL4
NPTXR
CBX6
CBY1
GTPBP1
JOSD1
Position on chr22 (Mb)
